# Fully Reduced and Mixed-Valent Multi-Copper Aggregates Supported by Tetradentate Diamino Bis(thiolate) Ligands

**DOI:** 10.1021/acs.inorgchem.3c00784

**Published:** 2023-06-13

**Authors:** Bo Wang, Justin Barnes, Skylar J. Ferrara, Stephen Sproules, Xiaodong Zhang, Joel T. Mague, James P. Donahue

**Affiliations:** † Department of Chemistry, Tulane University, 6400 Freret Street, New Orleans, Louisiana 70118-5638, United States; ‡ WestCHEM, School of Chemistry, University of Glasgow, Glasgow G12 8QQ, U.K.

## Abstract

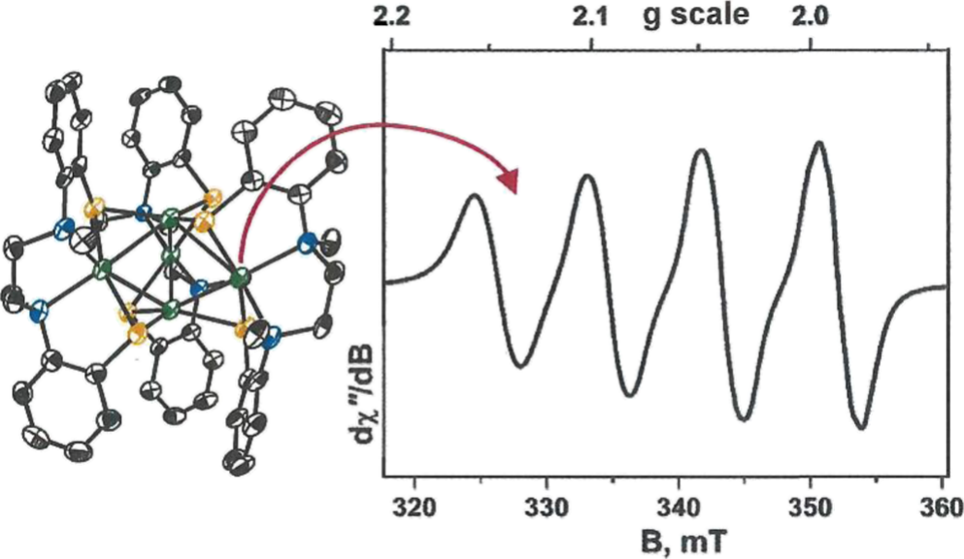

Tetradentate diamino bis(thiolate) ligands (l-N_2_S_2_(2−)) with saturated linkages between heteroatoms support fully reduced [(Cu(l-N_2_S_2_))_2_Cu_2_] complexes that bear relevance as an entry point toward molecules featuring the Cu_2_^I^Cu_2_^II^(μ_4_-S) core composition of nitrous oxide reductase (N_2_OR). Tetracopper [(Cu(l-N_2_(S^Me_2_^)_2_))_2_Cu_2_] (l-N_2_(S^Me_2_^H)_2_ = *N*^1^,*N*^2^-*bis*(2-methyl-2-mercaptopropane)-*N*^1^,*N*^2^-dimethylethane-1,2-diamine) does not support clean S atom oxidative addition but undergoes Cl atom transfer from PhICl_2_ or Ph_3_CCl to afford [(Cu(l-N_2_(S^Me_2_^)_2_))_3_(CuCl)_5_], **14**. When introduced to Cu(I) sources, the l-N_2_(S^Ar^H)_2_ ligand (l-N_2_(S^Ar^H)_2_ = *N*^1^,*N*^2^-*bis*(2-mercaptophenyl)-*N*^1^,*N*^2^-dimethylethane-1,2-diamine), made by a newly devised route from *N*^1^,*N*^2^-*bis*(2-fluorophenyl)-*N*^1^,*N*^2^-dimethylethane-1,2-diamine, ultimately yields the mixed-valent pentacopper [(Cu(l-N_2_S^Ar^_2_))_3_Cu_2_] (**19**), which has 3-fold rotational symmetry (*D*_3_) around a Cu_2_ axis. The single Cu^II^ ion of **19** is ensconced within an equatorial l-N_2_(S^Ar^)_2_(2−) ligand, as shown by ^14^N coupling in its EPR spectrum. Formation of **19** proceeds from an initial, fully reduced product, [(Cu(l-N_2_S^Ar^_2_))_3_Cu_2_(Cu(MeCN))] (**17**), which is *C*_2_ symmetric and exceedingly air-sensitive. While unreactive toward chalcogen donors, **19** supports reversible reduction to the all-cuprous state; generation of [**19**]^1–^ and treatment with S atom donors only return **19** because structural adjustments necessary for oxidative addition are noncompetitive with outer-sphere electron transfer. Oxidation of **19** is marked by intense darkening, consistent with greater mixed valency, and by dimerization in the crystalline state to a decacopper species ([**20**]^2+^) of *S*_4_ symmetry.

## Abstract 2

The diamino bis(thiolate) ligand *N*^1^,*N*^2^-*bis*(2-methyl-2-mercaptopropane)-*N*^1^,*N*^2^-dimethylethane-1,2-diamine (l-N_2_(S^Me_2_^)_2_(2−)) with saturated linkages between heteroatoms supports fully reduced [(Cu(l-N_2_(S^Me_2_^)_2_)_2_Cu_2_], which does not undergo S atom oxidative addition but undergoes Cl atom transfer from PhICl_2_ or Ph_3_CCl to afford [(Cu(l-N_2_(S^Me_2_^)_2_))_3_(CuCl)_5_]. The *N*^1^,*N*^2^-*bis*(2-mercaptophenyl)-*N*^1^,*N*^2^-dimethylethane-1,2-diamine ligand ((l-N_2_(S^Ar^)_2_(2−)) forms mixed-valent [(Cu(l-N_2_S^Ar^_2_))_3_Cu_2_] (**19**) from Cu(I) sources. Neither **19** nor fully reduced [**19**]^1−^ reacts with S atom donors to form sulfide-bridged species. Oxidation of **19** affords [**19**]^+^ in solution, which dimerizes to an *S*_4_-symmetric dication upon crystallization.

## Introduction

Nitrous oxide functions as a terminal electron acceptor in anaerobic respiration by soil-dwelling bacteria, which execute this two-electron reduction as the final step in the denitrifying pathway in the nitrogen cycle through the agency of nitrous oxide reductase (N_2_OR).^1^ Nitrous oxide reductase has elicited considerable interest because N_2_O is a potent greenhouse gas whose atmospheric concentration is steadily increasing^2^ with an atmospheric lifetime that is ∼300-fold greater than that of CO_2_^3^ and because it is projected to be the foremost ozone depleting substance in the 21st century.^4^ Further attention has been drawn to N_2_OR because, early in its characterization, it was identified as a multicopper enzyme with an active site composition that is unique in biology.

**Figure 1:**
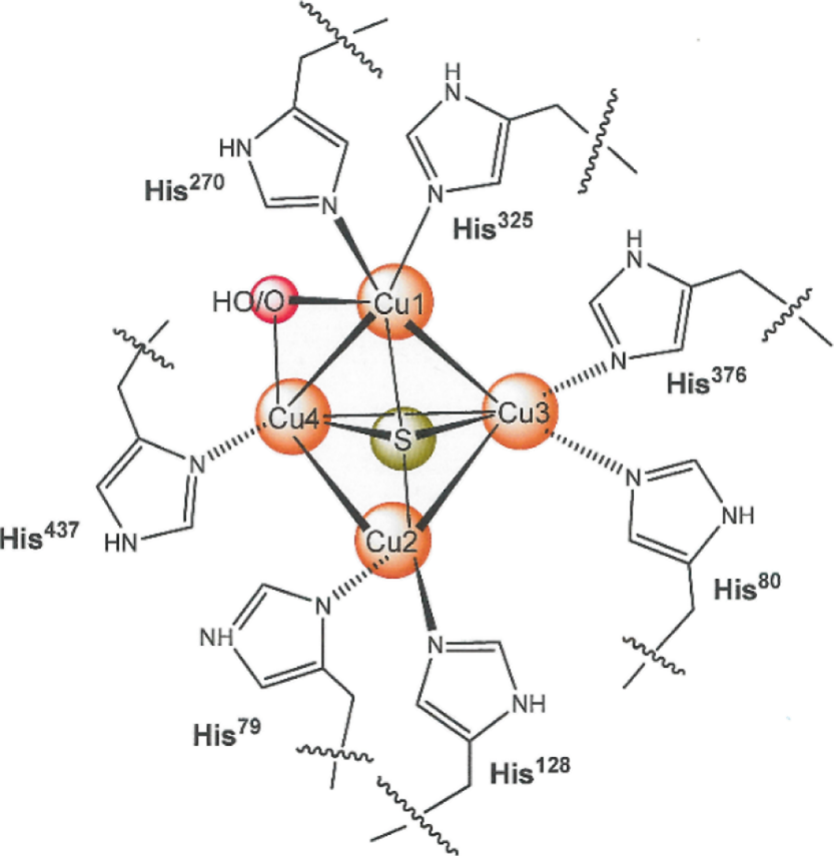
N_2_O reductase catalytic site, as revealed by X-ray crystallography. The numbering of histidine residues is from the *Pseudomonas nautica* (*Marinobacter hydrocarbonoclasticus*) structure.^5^

A consensus description of the N_2_O reductase active site, commonly designated as Cu_Z_/Cu_Z_*, emerged in the period 2000–2006 from the collective insights provided by a succession of independent crystal structures^5−9^ of the enzyme from several species of Gram-negative bacteria. Four copper ions arrayed in a butterfly fold with a μ_4_-S^2–^ ligand ensconced between them, albeit with a somewhat asymmetric placement, are its defining features (Figure 1). In its resting state, the catalytic site bears an oxygenous ligand—either H_2_O or OH—along one copper–copper edge, which is the presumed site of N_2_O substrate binding in the course of catalysis. One structural study of an N_2_O reductase preparation under strictly anaerobic conditions from *Pseudomonas stutzeri*, however, revealed a second S^2–^ at this position.^10^ Seven histidine ligands complete the coordination environment about this Cu_4_S core, five of them *via* the Nε of the imidazole ring (Figure 1). A redox titration of N_2_O reductase, as monitored by EPR spectroscopy, demonstrated that the onset of catalytic activity correlated with reduction to the all-cuprous Cu_4_^I^S state.^11^ Thus, the two-electron reduction of N_2_O is coupled to a cycling between Cu_4_^I^S and Cu_2_^I^Cu_2_^II^(μ_4_-S) redox levels.

**Figure 2:**
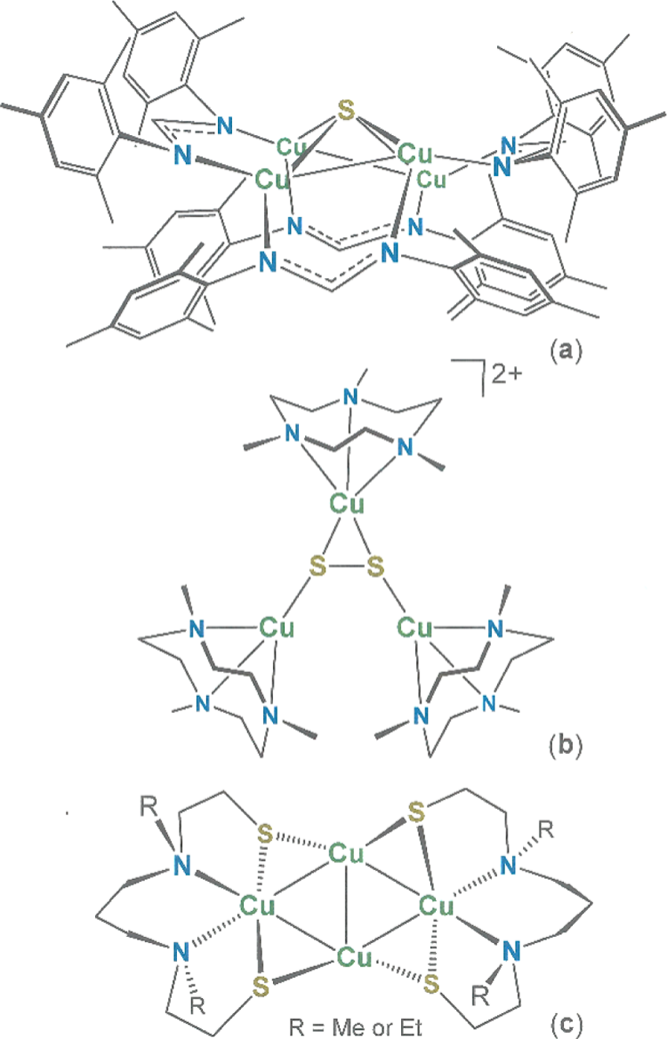
Model systems of Mankad (a) and Tolman (b). A tetracopper(I) compound reported by Henkel and co-workers (c).

The foregoing facts bring particular relevance to the synthesis and properties of small molecules that bear a Cu_4_S core or otherwise present compositional similarity to the Cu_Z_/Cu_Z_* site. A model system devised by Mankad and co-workers (Figure 2a) with mesityl-substituted formamidinate supporting ligands incorporates the Cu_4_(μ_4_-S) core and, despite not being reducible to the all-cuprous state, has been reported to support a N_2_O-to-N_2_ turnover.^12,13^ A topologically similar Cu_4_S compound with charge-neutral, amine-bridged *bis*(phosphine) ligands has also been described as supporting N_2_O reduction but without preservation of the Cu_4_S core composition.^14,15^ Somewhat earlier, the Tolman laboratory reported a mixed-valent tricopper compound featuring a disulfide ligand in a μ_3_-η^2^,η^1^-S,η^1^,S′ bridging motif that was also competent to reduce N_2_O to N_2_ (Figure 2b).^16^ Other small-molecule copper complexes that have been devised as N_2_O active site models have been reviewed by Mankad.^17^

In 2003, a brief report by Henkel and co-workers related the preparation and structure of a pair of charge-neutral tetracuprous compounds supported by tetradentate diamino *bis*(thiolate) (l-N^R^_2_S_2_(2−)) ligands (Figure 2c) but with no attending account of their reactivity.^18^ Considering their fully reduced state, these compounds appeared plausible as candidates to a Cu_2_^I^Cu_2_^II^(μ_4_-S) model compound by direct S atom addition. We initiated an investigation of this point using l-N_2_S_2_(2−) ligands that incorporate, for effect, either alkyl- or aryl-type thiolates, whose results are summarized here. In this context, a recent review by Denny and Darensbourg of the structural coordination chemistry of l-N_2_S_2_(2−) ligands usefully summarizes the general properties of their complexes with the first–row transition metals.^19^

## General Considerations

**Scheme 1:**
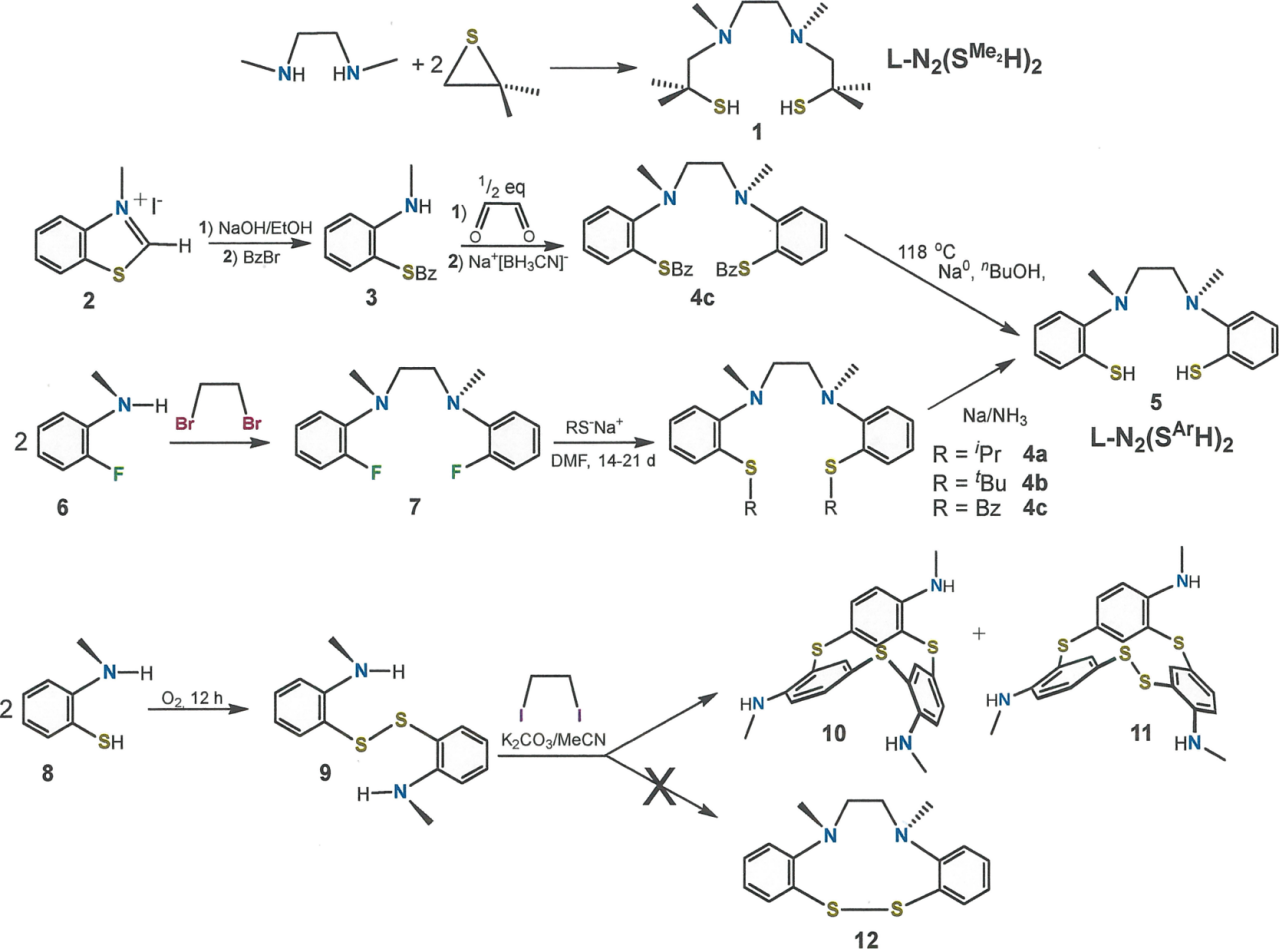
Summary of Ligand Syntheses Ligand **1** was prepared via the literature method.^21^ While also a known compound, **5** was synthesized by the newly devised route **6** → **7** → **4a**/**4b**/**4c** → **5** as an alternative to the previously disclosed sequence of **2** → **3** → **4c** → **5**.^26^

**Scheme 2:**
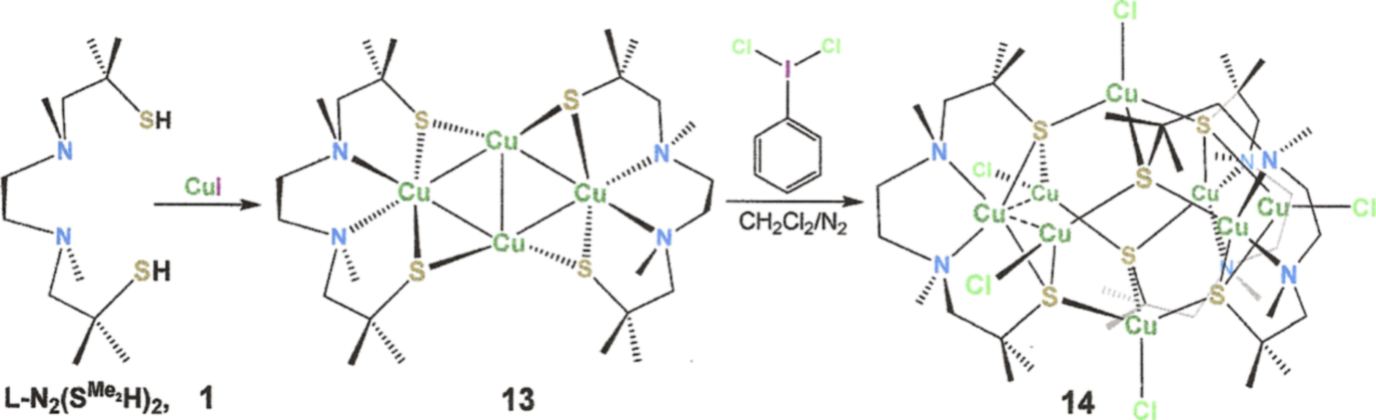
Synthesis of Copper Compounds with l-N_2_(S^Me_2_^H)_2_

**Scheme 3:**
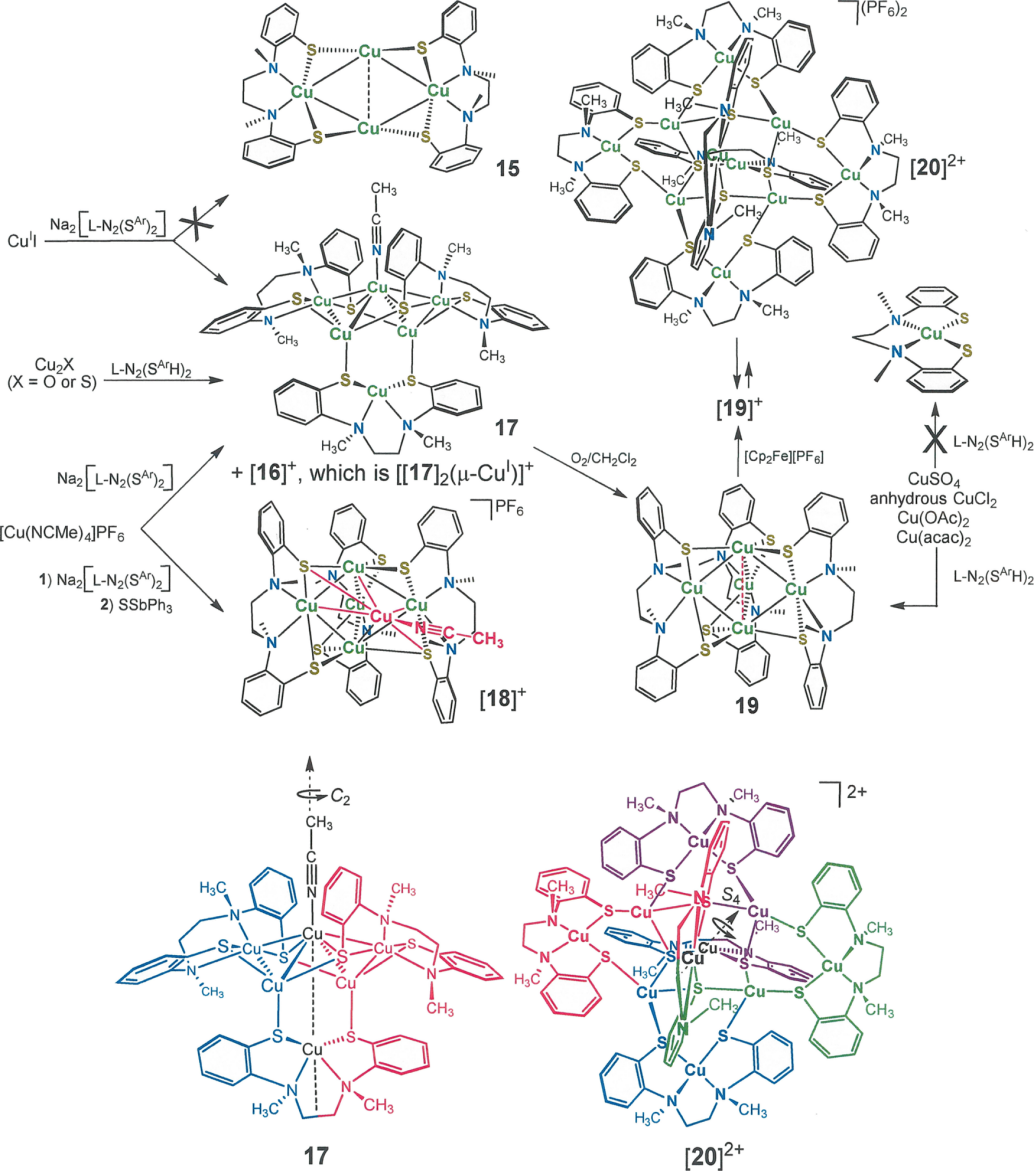
Synthesis of Copper Compounds with [l-N_2_(S^Ar^)_2_]^2–^ The colored image of **17** (bottom left) emphasizes its 2-fold rotational symmetry. The *C*_2_ axis coincides with the MeCN ligand and the Cu^I^ ions shown in black. The colored image of [**20**]^2+^ (bottom right) highlights the *S*_4_ axis that contains the Cu ions in black and bisects the [Cu(l-N_2_S^Ar^_2_)] fragments at front (vertically oriented) and back (horizontally arranged). Successive clockwise executions of the *S*_4_ operation moves the red fragment as follows: **red** → **violet** → **green** → **blue** → **red**.

**Chart 1:**
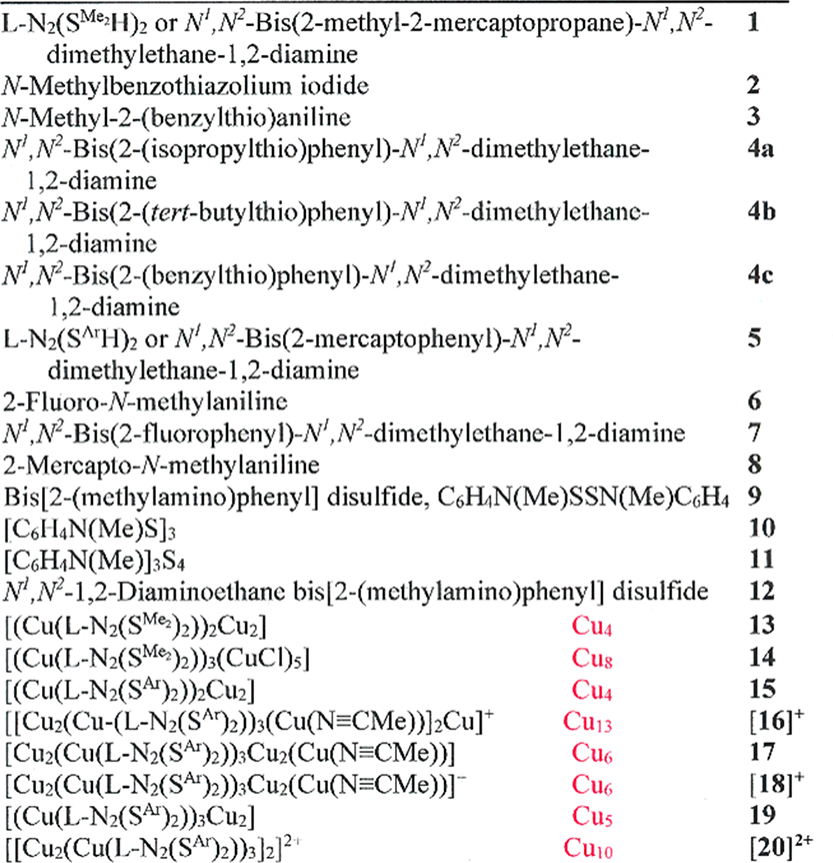
Numbering identification of compounds.

All reactions and manipulations were performed under a pure dinitrogen or argon atmosphere using either modified Schlenk techniques or an inert atmosphere box. Solvents used for syntheses and crystallizations were dried with a system of drying columns from the Glass Contour Company (CH_2_Cl_2_, Et_2_O, and THF), freshly distilled according to standard procedures (MeOH and CH_3_CN),^20^ or purchased in an anhydrous form suitable for immediate use (DMF). *N*,*N*′-*Bis*(2-methyl-2-mercaptopropane)-*N*,*N*′-dimethylethane-1,2-diamine (l-N_2_(S^Me_2_^H)_2_**1**) was prepared following a literature protocol.^21^ The identifying numbers for all compounds are defined in Chart 1 and pictorially in Schemes 1, 2, and 3. Other reagents, and all solvents used in column chromatography purifications, were used as received from commercial sources. Silica columns were run in the open air using 60–230 μm silica (Dynamic Adsorbents).

All NMR spectra were recorded at 25 °C with a Varian Unity Inova spectrometer operating at 400 or 100.5 MHz for ^1^H and ^13^C, respectively, or with a Bruker DSX 300 operating at 300 or 75 MHz for ^1^H and ^13^C, respectively, and were referenced to the partially labeled solvent residual. Mass spectra were obtained by either MALDI-TOF (Bruker Autoflex III instrument) or by electrospray ionization methods (Bruker microOTOF with Agilent Technologies 1200 Series LC). Progress in the formation of compounds **4a**–**4c** was monitored with a Varian Model 450 GC-MS operating with a Model 300 MS quadupole. The UV–vis spectra were acquired on a Hewlett-Packard 8752A diode array spectrometer, while the X-band EPR spectra were recorded on a Bruker ELEXSYS E500 spectrometer with simulations performed using *XSophe*.^22^ Elemental analyses were performed by Midwest Microlab, LLC of Indianapolis, IN, or by Kolbe Microanalytical Laboratory in Oberhausen, Germany. Electrochemical measurements were performed using a CHI 620C electrochemical analyzer workstation with a Ag/AgCl reference electrode, glassy carbon working electrode, Pt wire auxiliary electrode, and [^*n*^Bu_4_N][PF_6_] as the supporting electrolyte. Under these conditions, the Cp_2_Fe^+^/Cp_2_Fe couple consistently occurred at +0.50 V. A description of the methods of crystal growth and of the procedures for X-ray data collection, structure solution, and refinement is deferred to the Supporting Information. Selected unit cell and refinement statistics for all crystallographically characterized compounds are collected in Tables 1 and 2. A description of the computational methods, theory level, and basis sets implemented in the calculations involving **19** is also found in the Supporting Information.

**Table 1:** Crystal and Refinement Data for **4a**·2(HCl), **4b**, **4c**, **7**, and **9–11**

Compound	**4a**·2(HCl)	**4b**	**4c**
formula	C_22_H_34_Cl_2_N_2_S_2_	C_24_H_36_N_2_S_2_	C_30_H_32_N_2_S_2_
FW	461.53	416.67	484.69
xtl system	monoclinic	triclinic	monoclinic
space group	*P*2_1_/*c*	*P*-1	*P*2_1_/*n*
color, habit	colorless plate	thick white wedge	colorless block
size, mm	0.02 × 0.21 × 0.42	0.07 × 0.31 × 0.47	0.12 × 0.12 × 0.15
*a*, Å	13.0510(4)	7.0210(5)	9.7229(7)
*b*, Å	10.1657(3)	9.4375(6)	9.3312(7)
*c*, Å	9.1259(3)	10.0316(7)	14.2753(10)
α, deg.	90	88.089(1)	90
β, deg.	95.978(1)	76.270(1)	100.6420(11)
γ, deg.	90	69.802(1)	90
*V*, Å^3^	1204.17(6)	605.14(7)	1272.87(16)
*T*, K	150	150	150
*Z*	2	1	2
*d*, g/cm^3^	1.273	1.143	1.265
reflections collected	2308	10741	22040
independent data	2308	2804	3148
restraints	0	0	41
parameters refined	130	144	155
*R*1,^a^^,^^b^*wR*2^b^^,^^c^	0.0349, 0.0881	0.0395, 0.1102	0.0390, 0.0976
*R*1,^a^^,^^d^*wR*2^c^^,^^d^	0.0393, 0.0909	0.0463, 0.1140	0.0500, 0.1056
GooF^e^	1.066	1.116	1.045

compound	**7**	**9**	**10**	**11**·CH_2_Cl_2_
formula	C_16_H_18_F_2_N_2_	C_14_H_16_N_2_S_2_	C_21_H_21_N_3_S_3_	C_22_H_23_Cl_2_N_3_S_4_
FW	276.32	276.41	411.59	528.57
xtl system	monoclinic	monoclinic	monoclinic	triclinic
space group	*P*2_1_/*n*	*P*2_1_/*c*	*P*2_1_/*c*	*P*1̅
color, habit	colorless block	yellow column	yellow block	yellow column
size	0.11 × 0.11 × 0.21	0.11 × 0.14 × 0.28	0.09 × 0.13 × 0.19	0.03 × 0.05 × 0.17
*a*, Å	5.4275(1)	12.4663(3)	11.5996(9)	8.2111(2)
*b*, Å	8.3334(2)	7.9419(2)	10.5464(8)	10.6229(2)
*c*, Å	15.7929(3)	14.5714(3)	15.7796(12)	15.0778(3)
α, deg.	90	90	90	109.789(1)
β, deg.	98.996(1)	110.3330(9)	90.044(1)	91.824(1)
γ, deg.	90	90	90	101.8271)
*V*, Å^3^	705.52(3)	1352.76(6)	1930.4(3)	1203.90(4)
*T*, K	150	150	150	150
*Z*	2	4	4	2
*d*, g/cm^3^	1.301	1.357	1.416	1.458
reflections collected	5211	10484	34564	13015
independent data	1322	2438	4973	4648
restraints	0	0	0	0
parameters refined	92	165	247	283
*R*1,^a^^,^^b^*wR*2^b^^,^^c^	0.0368, 0.0883	0.0291, 0.0776	0.0520, 0.1368	0.0493, 0.1184
*R*1,^a^^,^^d^*wR*2^c^^,^^d^	0.0446, 0.930	0.0326, 0.0801	0.0659, 0.1495	0.0664, 0.1292
GooF^e^	1.103	1.044	1.047	1.043

a *R*1 = Σ||*F*_o_| – |*F*_c_||/Σ|*F*_o_|.

b *R* indices for data cut-off at *I* > 2σ(*I*).

c *wR*2 = {Σ[*w*(*F*_o_^2^ – *F*_c_^2^)^2^]/Σ*w*(*F*_o_^2^)^2^}^1/2^; *w* = 1/[σ^2^(*F*_o_^2^) + (*xP*)^2^ + *yP*], where *P* = (*F*_o_^2^ + 2*F*_c_^2^)/3.

d *R* indices for all data.

e GooF = {Σ[*w*(*F*_o_^2^ – *F*_c_^2^)^2^]/(*n* – *p*)}^1/2^, where *n* = number of reflections and *p* is the total number of parameters refined.

**Table 2:** Crystal and Refinement Data for **13**, **14**, [**16**][PF_6_]·^1^/_2_[**17**], [**18**][PF_6_], **19**·2THF, and [**20**][PF_6_]_2_

compound	**13**	**14**	[**16**][PF_6_]·^1^/_2_[**17**]
cocryst. solvent	^*t*^BuOMe·2MeCN	3.05C_6_H_6_·0.45C_6_H_4_Me_2_·0.5H_2_O	^*t*^BuOMe·2MeCN
formula	C_24_H_52_Cu_4_N_4_S_4_	C_58_H_100.75_Cl_5_Cu_8_N_6_O_0.5_S_6_	C_134_H_160.5_Cu_16_F_6_N_19.5_OPS_15_
FW	779.09	1768.12	3702.82
xtl system	monoclinic	tetragonal	monoclinic
space group	*P*2_1_/*c*	*P*4_2_/*n*	*C*2/*c*
color, habit	colorless block	dark blue plate	colorless plate
size, mm	0.16 × 0.27 × 0.31	0.16 × 0.27 × 0.31	0.06 × 0.13 × 0.16
*a*, Å	13.8940(11)	27.8548(9)	39.4732(12)
*b*, Å	12.0059(10)	27.8548(9)	28.2023(9)
*c*, Å	20.8983(17)	19.1577(9)	32.1982(1)
α, deg.	90	90	90
β, deg.	99.227(1)	90	109.113(1)
γ, deg.	90	90	90
*V*, Å^3^	3440.9(5)	14864.3(12)	33868.2(18))
*T*, K	150	170	100
Z	4	8	8
*d*, g/cm^3^	1.504	1.580	1.452
reflections collected	66379	672248	183138
independent data	9775	20103	14810
restraints	0	9	12
parameters refined	337	687	1742
*R*1,^a^^,^^b^*wR*2^b^^,^^c^	0.0235, 0.0632	0.0503, 0.1255	0.0346, 0.0956
*R*1,^a^^,^^d^*wR*2^c^^,^^d^	0.0296, 0.0651	0.0755, 0.1501	0.0366, 0.0971
GooF^e^	1.056	1.147	1.069

compound	[**18**][PF_6_]	**19**	[**20**][**PF**_**6**_]_**2**_
cocryst. solvent	C_5_H_12_	2THF	2MeCN
formula	C_55_H_69_Cu_6_F_6_N_7_PS_6_	C_56_H_70_Cu_5_N_6_O_2_S_6_	C_100_H_102_Cu_10_F_12_N_14_P_2_S_12_
FW	1546.84	1369.24	2810.01
xtl system	triclinic	monoclinic	tetragonal
space group	*P*-1	*C*2/*c*	*I*4_1_/*a*
color, habit	blue plate	green–yellow block	black wedge
size	0.09 × 0.11 × 0.24	0.03 × 0.05 × 0.17	0.12 × 0.40 × 0.41
*a*, Å	10.2198(18)	40.455(3)	27.910(3)
*b*, Å	17.198(3)	11.6468(9)	27.910(3)
*c*, Å	17.552(3)	27.274(2)	17.459(2)
α, deg.	82.128(3)	90	90
β, deg.	83.047(2)	114.855(1)	90
γ, deg.	81.000(3)	90	90
*V*, Å^3^	3002.5(9)	11660.4(16)	13600(4)
*T*, K	100	100	100
*Z*	2	8	4
*d*, g/cm^3^	1.711	1.560	1.372
reflections collected	14336	111732	79563
independent data	14336	15694	4796
restraints	0	0	73
parameters refined	707	682	410
*R*1,^a^^,^^b^*wR*2^b^^,^^c^	0.0841, 0.2054	0.0336, 0.0834	0.1147, 0.3281
*R*1,^a^^,^^d^*wR*2^c^^,^^d^	0.1297, 0.2312	0.0477, 0.0873	0.1255, 0.3381
GooF^e^	0.966	1.026	1.126

a *R*1 = Σ||*F*_o_| – |*F*_c_||/Σ|*F*_o_|.

b *R* indices for data cut-off at *I* > 2σ(*I*).

c *wR*2 = {Σ[*w*(*F*_o_^2^ – *F*_c_^2^)^2^]/Σ*w*(*F*_o_^2^)^2^}^1/2^; *w* = 1/[σ^2^(*F*_o_^2^) + (*xP*)^2^ + *yP*], where *P* = (*F*_o_^2^ + 2*F*_c_^2^)/3.

d *R* indices for all data.

e GooF = {Σ[*w*(*F*_o_^2^ – *F*_c_^2^)^2^]/(*n* – *p*)}^1/2^, where *n* = number of reflections and *p* is the total number of parameters refined.

### Syntheses

#### *N*^1^,*N*^2^-*Bis*(2-fluorophenyl)-*N*^1^,*N*^2^-dimethylethane-1,2-diamine, **7**

In a 100 mL Schlenk flask equipped with a reflux condenser, 2-fluoro-*N*-methylaniline (4.00 g, 32.0 mmol), 1,2-dibromoethane (3.01 g, 16.0 mmol), and diisopropylethylamine (4.14 g, 32.0 mmol) were heated overnight at 135 °C. After cooling to room temperature, the reaction mixture was extracted with H_2_O (50 mL) and CH_2_Cl_2_ (45 mL). A 44 mL portion of 0.5 M KOH solution was added to the aqueous layer, and an extraction with another 45 mL of CH_2_Cl_2_ was conducted. The combined organic phases were dried with MgSO_4_ and filtered through Celite. All volatiles were then removed *via* rotary evaporation, and the residual oil was recrystallized from EtOH three times to yield analytically pure **7** as colorless crystals. Yield: 1.83 g, 41.4%. MP: 32 °C. ^1^H NMR (δ, C_6_D_6_): 6.97–6.73 (m, 4 H, aromatic CH), 6.67–6.51 (m, 4 H, aromatic CH), 3.27 (s, 4 H, −CH_2_CH_2_−), 2.54 (s, 6 H, N(CH_3_)). ^13^C NMR (δ, C_6_D_6_): 155.13 (d, *J* = 242 Hz), 140.08 (d, *J* = 9 Hz), 124.40 (d, *J* = 4 Hz), 120.45 (d, *J* = 8 Hz), 118.60 (d, *J* = 4 Hz), 116.25 (d, *J* = 21 Hz), 53.07 (t, *J* = 3 Hz), 39.61 (s). Anal. Calcd for C_16_H_18_F_2_N_2_: C, 69.55; H, 6.57; N, 10.14. Found: C, 69.80; H, 6.80; N, 10.28.

#### *N*^1^,*N*^2^-*Bis*(2-(^*i*^propylthio)phenyl)-*N*^1^,*N*^2^-dimethylethane-1,2-diamine, **4a**

A portion of solid NaH (5.0 g, 0.209 mol) in a Schlenk flask was suspended in 200 mL of degassed, anhydrous DMF that had been transferred to it *via* a cannula. The flask was cooled in a dry ice–acetone bath, and 2-propanethiol (19.4 mL, 0.208 mol) was added in a dropwise fashion with vigorous stirring. Bubbles of H_2_(*g*) formed immediately. After being stirred for 5 h, the solution was warmed to ambient temperature. With an outward flow of N_2_, a solid portion of **7** (5.75 g, 20.8 mmol) was added to the flask, and the mixture was then heated to reflux for 21 days. The progress of the reaction was monitored by GC–MS. When the reaction was complete, the reaction mixture was diluted with H_2_O (2 L). This DMF–H_2_O mixture was then extracted with CH_2_Cl_2_ (3 × 200 mL), and the combined extracts were dried over Na_2_SO_4_. Following removal of Na_2_SO_4_ by filtration, the solvent was removed from the filtrate under reduced pressure to yield pure **4a** as an oil. Yield: 7.5 g, 19 mmol, 93% based on **7**. ^1^H NMR (δ, CD_2_Cl_2_): 7.30 (m, 2 H, aromatic CH), 7.21–7.09 (m, 4 H, aromatic CH), 7.08–6.99 (m, 2 H, aromatic CH), 3.55 (hept, *J* = 6.7 Hz, 2 H, −C*H*(CH_3_)_2_), 3.24 (s, 4 H, −CH_2_CH_2_−), 2.80 (s, 6 H, N(CH_3_)), 1.35 (d, *J* = 6.2 Hz, 12 H, −CH(C*H*_3_)_2_). ^13^C NMR (δ, CD_2_Cl_2_): 152.27, 132.94, 129.50, 126.20, 123.82, 121.00, 54.49, 42.03, 35.27, 23.24.

#### *N*^1^,*N*^2^-*Bis*(2-(^*t*^butylthio)phenyl)-*N*^1^,*N*^2^-dimethylethane-1,2-diamine, **4b**

The procedure and scale followed were analogous to those employed for the preparation of **4a** except that 8 equiv (0.1664 mol) of both NaH and *tert*-butylthiol was introduced to **7** rather than 10 equiv. The reaction was observed to be complete in 14 days, and a series of work-up steps identical to those used for **4a** produced **4b** as a white solid. Yield: 7.79 g, 90% based on **7**. ^1^H NMR (δ, CD_2_Cl_2_): 7.48 (d, 2 H, aromatic CH), 7.36–7.19 (m, 2 H, aromatic CH), 7.17–7.04 (m, 2 H, aromatic CH), 6.91 (m, 2 H, aromatic CH), 3.41 (s, 4 H, −CH_2_CH_2_−), 2.79 (s, 6 H, N(CH_3_)), 1.23 (s, 18 H, C(CH_3_)_3_). ^13^C NMR (δ, CD_2_Cl_2_): 157.70, 140.41, 129.70, 126.85, 121.62, 119.92, 55.60, 47.35, 41.09, 31.09. Anal. Calcd for C_24_H_36_N_2_S_2_: C, 69.18; H, 8.71; N, 6.72. Found, C, 69.42; H, 8.68; N, 6.85.

#### *N*^1^,*N*^2^-*Bis*(2-(benzylthio)phenyl)-*N*^1^,*N*^2^-dimethylethane-1,2-diamine, **4c**

The procedure followed was analogous to that employed for the preparation of **4a** except that 4 equiv of NaH and benzylthiol were introduced to **7** rather than 10 equiv. No further progress in the reaction was noted after 14 days. Crude **4c** was purified on a silica column eluted with 95:5 hexanes/CH_2_Cl_2_. Yield: 10% based on **7**. *R*_f_: 0.6 in 95:5 hexanes/CH_2_Cl_2_. ^1^H NMR (δ, CD_2_Cl_2_): 7.43–7.18 (m, 10 H, aromatic CH), 7.09–6.58 (m, 8 H, aromatic CH), 3.61 (s, 4 H, −SCH_2_Ph), 3.36 (s, 4 H, −CH_2_CH_2_−), 2.85 (s, 6 H, −N(CH_3_)).

#### *N*^1^,*N*^2^-*Bis*(2-(^*i*^propylthio)phenyl)-*N*^1^,*N*^2^-dimethylethane-1,2-diamine *Bis*(hydrochloride), **[4a·H**_**2**_**]Cl**_**2**_

A portion of *N*^1^,*N*^2^-*bis*(2-(^*i*^propylthio)phenyl)-*N*^1^,*N*^2^-dimethylethane-1,2-diamine (0.500 g, 1.29 mmol) was added to a Schlenk flask containing concentrated HCl (50 mL), and the mixture was refluxed overnight under a N_2_ atmosphere. After cooling to ambient temperature, the solution was reduced to dryness under vacuum to afford [**4a·H**_**2**_]**Cl**_**2**_ as a white solid. Crystals of X-ray diffraction quality were obtained by layered diffusion of *n*-pentane into an ethanol solution. ^1^H NMR (δ, CD_3_OD): 7.63–7.61 (m, 3 H, aromatic CH), 7.46–7.43 (m, 3 H, aromatic CH), 7.30–7.27 (m, 2 H, aromatic CH), 3.60 (hept, *J* = 4 Hz, 2 H, −C*H*(CH_3_)_2_), 3.51 (s, 4 H, −CH_2_CH_2_−), 3.04 (s, 6 H, −N(CH_3_)), 1.39 (d, *J* = 6.7 Hz, 12 H, −CH(C*H*_3_)_2_). The −NH protons were not observed due to exchange with CD_3_OD.

#### *N*^1^,*N*^2^-*Bis*(2-mercaptophenyl)-*N*^1^,*N*^2^-dimethylethane-1,2-diamine *Bis*(hydrochloride), [l-(NH)_2_(S^Ar^H)_2_]Cl_2_, **[5·H**_**2**_**]Cl**_**2**_

A solid portion of **4b** (0.200 g, 0.48 mmol) was added to a flask, followed by 15 mL of concentrated HCl. The mixture was refluxed overnight and, following cooling to ambient temperature, the solution was reduced to dryness under vacuum to afford a brown solid. Compound [**5**·H_2_]Cl_2_ is hygroscopic but otherwise stable in air. Yield: 0.174 g, 96%. ^1^H NMR (δ, CD_3_OD): 7.66 (d, *J* = 7.9 Hz, 2 H, aromatic CH), 7.55 (d, *J* = 6.7 Hz, 4 H, aromatic CH), 7.45–7.29 (m, 2 H, aromatic CH), 3.54 (s, 4 H, −CH_2_CH_2_−), 3.10 (s, 6 H, −N(CH_3_)). The −NH and −SH protons are not observed due to exchange with CD_3_OD.

#### *N*^1^,*N*^2^-*Bis*(2-mercaptophenyl)-*N*^1^,*N*^2^-dimethylethane-1,2-diamine, **5**

A three-necked Schlenk flask, equipped with a stir bar and a dry ice condenser, was charged with *N*^1^,*N*^2^-*bis*(2-(isopropylthio)phenyl)-*N*^1^,*N*^2^-dimethylethane-1,2-diamine (5.00 g, 12.9 mmol) and 40 mL of hexanes. A dry ice–acetone bath was used to cool the mixture to −78 °C. Gaseous ammonia was admitted via a flow control adapter fitted to one of the side necks of the flask until the total volume of condensed NH_3_/hexanes was approximately 100 mL. Sodium metal (3.45 g, 150 mmol) was slowly added in small portions with stirring, which yielded a dark blue solution. After being stirred for 10 h, the reaction mixture was quenched with NH_4_Cl (8.30 g, 155 mmol), and the cooling bath was allowed to slowly warm to ambient temperature over ∼12 h. Ammonia was allowed to evaporate and vent itself through the pressure release system of the Schlenk line. Aqueous HCl was then added, and the mixture was extracted with CHCl_3_ (3 × 100 mL). The combined extracts were dried over Na_2_SO_4_ and then filtered. The solvent was removed under reduced pressure to afford *N*^1^,*N*^2^-*bis*(2-(mercaptophenyl)-*N*^1^,*N*^2^-dimethylethane-1,2-diamine (**l**-**N**_**2**_**(S**^**Ar**^**H)**_**2**_, **5**) as a white solid with a pale blue tint. Compound **5** is mildly sensitive to air. X-ray diffraction quality crystals deposit from a hexane solution held at −20 °C for several days. Yield: 3.6 g, 11.8 mmol, 92%. ^1^H NMR (δ, CD_2_Cl_2_): 7.28 (d, 2 H, aromatic CH), 7.12 (m, 2 H, aromatic CH), 7.07 (m, 2 H, aromatic CH), 6.97 (m, 2 H, aromatic CH), 5.00 (s, 2 H, −SH), 3.07 (s, 4 H, −CH_2_CH_2_−), 2.66 (s, 6 H, −N(CH_3_)). ^13^C NMR (δ, CD_2_Cl_2_): 149.14, 132.20, 128.78, 125.76, 125.15, 122.11, 54.46, 42.59.

#### 2,8,14-Trithiatetracyclo[13.3.1.1^3,7^.1^9,13^]heneicosa-1(19),3,5,7(21),9,11,13(20),15,17-Nonaene, 6,12,18-Tris(*N*-methylamine), **10**, and 2,8,14,15-Tetrathiatetracyclo[14.3.1.1^3,7^.1^9,13^]docosa-1(20),3,5,7(22),9,11,13(21),16,18-nonaene, 6,12,19-Tri(*N*-methylamine)-, **11**

A portion of 2-(methylamino)benzenethiol was dissolved in MeCN and stirred overnight in the open air to produce 2,2′-disulfanediylbis(*N*-methylaniline), **9**. The solvent was removed under reduced pressure. A separate three-necked flask was charged with K_2_CO_3_ (0.492 g, 3.56 mmol) and 100 mL of dry MeCN under N_2_. To this flask, separate solutions of 2,2′-disulfanediylbis(*N*-methylaniline) and 1,2-diiodoethane, both in dry MeCN, were added in a dropwise fashion at the same time. The solution color changed from yellow to red-brown after overnight stirring, and formation of a precipitate was observed. After being stirred for 2 days, the mixture was filtered, and the filtrate was extracted with hexanes. The solvent was removed from the combined extracts under reduced pressure to afford a crude product mixture. This product mixture was separated on a silica column eluted with 1:1 hexanes/CH_2_Cl_2_. *R*_f_: 0.4 for **10** and 0.1 for **11**. Crystals for both **10** and **11** were obtained by diffusion of *n*-pentane vapor into THF solutions. ^1^H NMR for **10** (δ, CDCl_3_): 7.22 (dd, *J* = 8.3, 2.2 Hz, 3 H, aromatic CH), 7.16 (d, *J* = 2.2 Hz, 3 H, aromatic CH), 6.45 (d, *J* = 8.4 Hz, 3 H, aromatic CH), 4.73 (br, 3 H), 2.88 (d, *J* = 4.8 Hz, 9 H, N(CH_3_)). ^1^H NMR for **11** (δ, CD_2_Cl_2_): 7.48 (d, *J* = 8 Hz, 1 H, aromatic CH), 7.35 (d, *J* = 8 Hz, 1 H, aromatic CH), 7.10 (d, *J* = 8 Hz, 1 H, aromatic CH), 7.05 (s, 1 H, aromatic CH), 6.98 (s, 1 H, aromatic CH), 6.74 (s, 1 H, aromatic CH), 6.57 (d, *J* = 9 Hz, 1 H, aromatic CH), 6.52 (d, *J* = 9 Hz, 1 H, aromatic CH), 6.38 (d, *J* = 9 Hz, 1 H, aromatic CH), 5.37 (br s, 1 H, −NH), 4.28 (br s, 1 H, −NH), 2.91 (d, *J* = 7 Hz, 3 H, N(CH_3_)), 2.80 (s, 6 H, N(CH_3_)). The third −NH signal that should be present was not apparent but was possibly obscured by another signal.

#### [(Cu(l-N_2_(S^Me_2_^)_2_))_2_Cu_2_], **13**

In a N_2_ glovebox, portions of [Cu(MeCN)_4_]PF_6_ (0.991 g, 2.66 mmol) and **1** (0.360 g, 1.36 mmol) were added to separate Schlenk flasks, which were then affixed to a Schlenk line. Addition of dry THF (20 mL) to **1** formed a colorless solution; subsequent addition of NaHBEt_3_ (2.7 mL, 1.0 *M* in THF, 2.7 mmol) in a dropwise fashion at room temperature to the stirring solution of **1** was attended by bubbling of H_2_(*g*). After ∼2 h, a white suspension was observed. The [Cu(MeCN)_4_]PF_6_ was dissolved in dry MeCN (25 mL) and added *via* a cannula to the stirring Na_2_[**1**] solution, whereupon a yellow color was induced. The reaction mixture was stirred overnight, after which time the solvent was removed *in vacuo* to afford an orange-yellow residue. To this reside, dry, degassed toluene (∼40 mL) was added to form an orange-yellow suspension, which was stirred for ∼2 h. The suspension was filtered through a packed Celite pad, and the filtrate was then concentrated under reduced pressure to yield an orange-yellow solid. This solid was washed with copious amounts of *n*-pentane and then dried *in vacuo* to afford a yellow powder. Crystals of X-ray diffraction quality are formed by diffusion of Et_2_O vapor into a concentrated toluene solution. Yield: 0.337 g, 0.433 mmol, 65%. ^1^H NMR (δ, C_6_D_6_): 2.36 (s, 12 H, N(CH_3_)), 2.13–2.04 (multiple overlapped signals, 12 H, N(CH_2_−)), 1.91 (br s, 4 H, N(CH_2_−)), 1.82 (br s, 12 H, SC(CH_3_)_2_), 1.52 (br s, 12 H, SC(CH_3_)_2_). UV–vis [CH_2_Cl_2_, λ_max_, nm]: 320. MS (ESI^+^), calcd for [C_24_H_52_Cu_4_N_4_S_4_]^1+^, *m*/*z* 778.0195; observed: *m*/*z* 778.0240; error (δ): 5.88 ppm.

#### [(Cu(l-N_2_(S^Me_2_^)_2_))_3_(CuCl)_5_], **14**

To a 25 mL Schlenk flask charged with **13** (0.050 g, 0.064 mmol), dry and degassed CH_2_Cl_2_ (∼5 mL) was added via a syringe with stirring to yield a yellow solution. A solution of PhICl_2_ (6.4 mL, 0.01 M, 0.064 mmol) was added dropwise *via* a syringe over a period of ∼10 min, during which time the solution gradually assumed a dark blue/black color. This reaction mixture was stirred for ∼2 h, whereupon the solvent was removed under reduced pressure to afford a dark blue/black solid residue. This residue was washed with toluene, followed by Et_2_O and then was collected by filtration to yield a dark blue/black powder. Yield: 0.038 g, 91% based on PhICl_2_ as a limiting reagent. X-ray quality crystals were obtained from diffusing benzene into a concentrated *o*-dichlorobenzene solution. Compound **14** can be similarly obtained by implementation of Ph_3_CCl, instead of PhICl_2_, as the chlorinating agent. UV–vis [CH_2_Cl_2_, λ_max_, nm (ε)]: 236 (36000), ∼302 (sh, ∼10000), 452 (4500), 492 (4800). Anal. Calcd for C_36_H_78_N_6_S_6_Cl_5_Cu_8_: C, 29.35; H, 5.34; N, 5.71. Found: C, 28.95; H, 5.26; N, 5.61.

#### [[Cu_2_(Cu(l-N_2_(S^Ar^)_2_))_3_(Cu(N≡CMe))]_2_Cu][PF_6_]·1/2[Cu_2_(Cu(l-N_2_(S^Ar^)_2_))_3_(Cu(N≡CMe))], **[16][PF**_**6**_**]·^1^/_2_17**

A 100 mL Schlenk flask in an Ar box was charged with **5** (0.150 g, 0.493 mmol) and then affixed to a N_2_ Schlenk line. Dry THF (20 mL) was added via a syringe, followed by 2 equiv of NaEt_3_BH (1.0 M in THF, 0.985 mL, 0.985 mmol), which was attended by the liberation of bubbles of H_2_ gas. The reaction mixture was stirred overnight and then taken to dryness under reduced pressure to remove the BEt_3_ byproduct. A 15 mL portion of fresh, dry THF was then added to the flask. The solution was then transferred via a cannula to another Schlenk flask containing [Cu(MeCN)_4_]PF_6_ (0.367 g, 0.98 mmol). This mixture was stirred for 3 h, yielding an orange solution with a white precipitate. The white precipitate was removed by cannula filtration, and the filtrate was reduced to dryness under vacuum to produce an orange crude solid product. Diffusion of *n*-pentane into a THF solution of this crude product yielded small portions of colorless, square plate crystals suitable for interrogation by X-ray diffraction. This crystalline material, identified crystallographically as **17** co-crystallized with the Cu^1+^-bridged dimer [**16**][PF_6_], was extremely sensitive to water and oxygen and was not tractable to further characterization.

#### Synthesis of [Cu_2_(Cu(l-N_2_(S^Ar^_2_)))_3_(Cu(N≡CMe))][PF_6_], **[18][PF**_**6**_**]**

A 100 mL Schlenk flask in an Ar glovebox was charged with **5** (0.150 g, 0.493 mmol), and the flask was then affixed to a N_2_ Schlenk line. Dry THF (20 mL) was added *via* a syringe, followed by NaHBEt_3_ (1.0 M in THF, 0.985 mL, 0.985 mmol). The addition of NaHBEt_3_ was marked by the visible evolution of bubbles of H_2_ gas. The reaction mixture was stirred overnight at ambient temperature, whereupon all volatiles were removed under reduced pressure. A 15 mL portion of fresh, dry THF was added to the flask to redissolve the ligand dianion, and the solution was then transferred via a cannula to a second Schlenk flask containing [Cu(MeCN)_4_]PF_6_ (0.367 g, 0.985 mmol). This mixture was stirred for 3 h, yielding an orange solution with a white precipitate. A solid portion of Ph_3_SbS (0.0949 g, 0.246 mmol) was added, which immediately induced formation of a green color. Stirring was continued for 5 h. The solvent was removed under vacuum to afford a dark solid residue, which was then redissolved in CH_2_Cl_2_ and filtered. This crude product was further purified on a silica gel chromatography column eluted with 1:1 THF in hexanes. Yield: 0.040 g, 0.027 mmol, 17%. *R*_f_: 0.50 (1:1 THF/hexanes). ^1^H NMR (δ, CD_2_Cl_2_): 7.36 (d, *J* = 7.9 Hz, 4 H, aromatic CH), 7.17–7.07 (overlapping m, 14 H, aromatic CH), 7.00–6.83 (overlapping m, 6 H, aromatic CH), 3.38–3.09 (m, 12 H, −CH_2_CH_2_−), 2.92–2.75 (m, 18 H, N(CH_3_)), 2.41 (s, 3 H, NCCH_3_). ^13^C NMR (δ, CD_2_Cl_2_): 151.1, 150.9, 137.4, 133.9, 130.6, 126.8, 126.4, 126.0, 125.6, 124.8, 124.3, 123.9, 123.5, 123.3, 121.9, 121.1, 120.7, 55.7, 55.4, 54.8, 50.8, 44.7, 43.3, 42.3, 41.1, 30.1. ESI-MS: 1287.91 (M^+^–MeCN). UV–vis [CH_2_Cl_2_, λ_max_, nm (ε)]: 332 (14500), 453 (1200), 587 (1200).

#### [Cu_2_(Cu(l-N_2_(S^Ar^)_2_))_3_], **19**

A 100 mL Schlenk flask was charged with **5** (0.105 g, 0.345 mmol), followed by dry THF (20 mL) added via a syringe. After the ligand fully dissolved, 1 equiv of [Cu(MeCN)_4_][PF_6_] (0.129 g, 0.345 mmol) was added under an outward flow of N_2_. Upon being stirred for ∼5 min, the color of the reaction mixture became dark. Stirring was continued for 12 h, after which time all volatiles were removed under reduced pressure. The residual solid was dissolved in a minimal volume of THF and filtered. Slow diffusion of *n*-pentane into this THF filtrate afforded dark crystals of [Cu_2_(Cu(l-N_2_(S^Ar^)_2_))_3_]·2(THF). Yield: 0.0853 g, 78% based on the Cu starting material. The product can also be purified by column chromatography on silica gel (*R*_f_: 0.3, 1:1 THF/hexanes). ^1^H NMR (δ, CD_2_Cl_2_): 7.33 (d, *J* = 7.8 Hz, 6 H, aromatic CH), 7.16 (d, *J* = 7.7 Hz, 6 H, aromatic CH), 7.08 (t, 6 H, aromatic CH), 6.91 (t, *J* = 7.5 Hz, 6 H, aromatic CH), 3.22 (s, 12 H, −CH_2_CH_2_−), 2.78 (s, 18 H, N(CH_3_)). ^13^C NMR (δ, CD_2_Cl_2_): 151.0, 133.9, 127.0, 125.6, 125.5, 121.9, 55.7, 43.3. UV–vis [CH_2_Cl_2_, λ_max_, nm (ε)]: 342 (8300), 454 (1500), 580 (1600). ESI-MS: 1224.9760, M^+^. Anal. Calcd for C_48_H_54_Cu_5_N_6_S_6_: C, 47.06; H, 4.44; N, 6.86. Found: C, 46.96; H, 4.35; N, 6.78.

#### [Cu_2_(Cu(l-N_2_(S^Ar^_2_)))_3_][PF_6_]/[[Cu_2_(Cu(l-N_2_(S^Ar^_2_)))_3_]_2_][PF_6_]_2_, **[19][PF**_**6**_**]/[20][PF**_**6**_**]**_**2**_

In a N_2_ glovebox, a 50 mL Schlenk flask was charged with freshly prepared and crystallized [Cp_2_Fe][PF_6_] (0.0135 g, 0.0408 mmol), followed by 15 mL of dry THF. Solid **19** (0.050 g, 0.0408 mmol) was added to the flask under N_2_ flow, and the color of the solution became dark yellow-green as it dissolved and then further darkened after overnight stirring. The solvent was removed under reduced pressure to yield a gray solid as the product along with some orange crystals of ferrocene. The crude solid was extracted with dry hexanes (3 × 30 mL) to remove Cp_2_Fe and then was dried again under vacuum. Yield: 0.0553 g, 0.020 mmol, 99%. In solution, [**19**][PF_6_] appears to be the dominant species, as gauged by ^1^H NMR and cyclic voltammetry (*vide infra*), but preparation of this oxidation product in crystalline form yields [**20**][PF_6_]_2_. A crystal of [**20**][PF_6_]_2_ suitable for X-ray diffraction was obtained by diffusion of Et_2_O into a saturated MeCN solution of crude [**19**][PF_6_]. ^1^H NMR (δ, CD_3_CN): 7.60 (d, *J* = 7.2 Hz, 6 H, aromatic CH), 7.35 (t, *J* = 7.7 Hz, 6 H, aromatic CH), 7.20 (d, *J* = 7.9 Hz, 6 H, aromatic CH), 7.03 (t, *J* = 7.5 Hz, 6 H, aromatic CH), 3.12 (s, 12 H, −CH_2_CH_2_−), 2.37 (s, 18 H, N(CH_3_)). UV–vis [CH_2_Cl_2_, λ_max_, nm (ε)]: 372 (27000), 477 (16000), 587 nm (21000).

## Results and Discussion

### Syntheses and Structures

An efficient and general means of synthesis of diamino dithiolate(2−) ligands (l-N_2_S_2_(2−)) with saturated connections between heteroatoms is by a ring-opening S_N_2 reaction between the corresponding diamine and the appropriate thiirane.^21,23−25^ When implemented with *N*^1^,*N*^2^-dimethylethylene-1,2-diamine in neat isobutylene sulfide, this approach reproducibly provides *N*^1^,*N*^2^-*bis*(2-methyl-2-mercaptopropane)-*N*^1^,*N*^2^-dimethylethane-1,2-diamine, L-N_2_(S^Me_2_^H)_2_ (1 in Scheme 1), in essentially quantitative yields.^21^ Compared to its analogues without the geminal methyl substituents, **1** offers both greater solubility and convenient simplicity by ^1^H NMR spectroscopy. The intrinsic chirality arising from the two tertiary amine nitrogen atoms in this ligand type necessarily imposes symmetry constraints on the coordination complexes formed (*vide infra*). As described by Henkel and co-workers for the synthesis of [(Cu(l-N^Me^_2_S_2_))Cu_2_] and [(Cu(l-N^Et^_2_S_2_))Cu_2_] (l-N^Me^_2_S_2_ = *N*^1^,*N*^2^-*bis*(2-mercaptoethane)-*N*^1^,*N*^2^-dimethylpropylenediamine(2−); l-N^Et^_2_S_2_ = *N*^1^,*N*^2^-*bis*(2-mercaptoethane)-*N*^1^,*N*^2^-diethylpropylenediamine(2−)),^18^ diamino dithiol **1** reacts in a straightforward fashion with Cu^1+^ sources to produce tetracopper(I) complex **13** (Scheme 2), which is isolable as colorless block crystals in yields of ∼65%.

**Figure 3:**
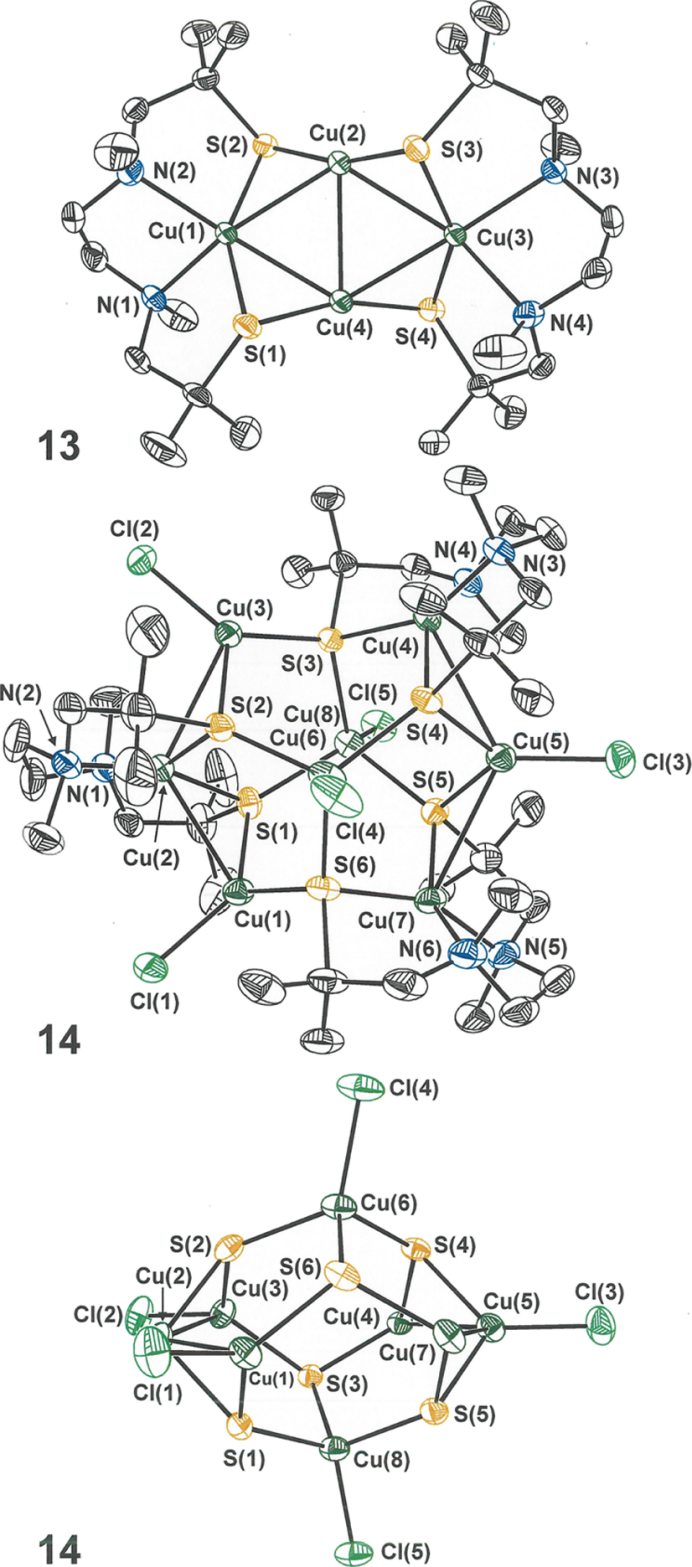
Thermal ellipsoid plots (50% probability) of **13** (top) and **14** (middle and bottom). The middle image shows the pseudo *C*_3_ axis of **14** orthogonal to the plane of the paper, while the bottom image shows the core of **14** with the pseudo *C*_3_ axis [along the Cu(6)···Cu(8) line] in the vertical direction. All H atoms are omitted for clarity.

Crystals of **13**, both with and without interstitial solvent, were examined by X-ray diffraction and reveal core structures that are essentially indistinguishable in their metrical details. The structure of **13** features a planar Cu_4_ core in which two cuprous ions (Cu(2) and Cu(4) in Figure 3, top) define the shared edge of two equilateral Cu_3_ triangles (Table 3). The complex is best described as a pair of [Cu(l-N_2_(S^Me_2_^)_2_)]^1–^ anions held in place and stabilized by a Cu_2_^2+^ axis. The triangular Cu_2_S “flaps” on the periphery of the Cu_4_ core [*e.g.*, the plane defined by Cu(1), Cu(2), and S(2)] alternate in an up-down fashion and meet the Cu_4_ mean plane at angles of 50–55°. The optical configuration at the amine nitrogen atoms within a given ligand, and for both ligands within the same Cu_4_ complex, is the same (*R*, in Figure 3), as this arrangement minimizes conformational strain and intraligand steric interactions. Consequently, the Cu_4_ complex as a whole is chiral with *D*_2_ point group symmetry. The three orthogonal *C*_2_ axes for **13** coincide with the Cu(2)–Cu(4) segment, the Cu(1)···Cu(3) line, and the perpendicular to the Cu_4_ plane at the Cu(2)–Cu(4) midpoint. Preservation of this structure in solution is indicated by ^1^H NMR data, showing only half of one l-N_2_S^Me_2_^_2_(2−) to be unique. Because **13** occurs in the centric space group *P*2_1_/*c*, the molecule with all *R* configuration at the nitrogen donor atoms necessarily has an inversion-related partner with the all *S* configuration. In all qualitative respects, the foregoing features enumerated for the structure of **13** are similar to the compounds reported from the Henkel laboratory. Possibly because ligand **1** presents more basic thiolate anions than does Henkel’s l-N^Me^_2_S_2_(2−), thereby providing for tighter binding to the Cu_2_^2+^ axial core, all intermetal distances in **13** are appreciably shorter than the corresponding values for [(Cu(l-N^Me^_2_S_2_))Cu_2_] (Table 3).

**Table 3:** Selected Interatomic Distances (Å), Angles (deg.) for Compounds **13**^b^, [(Cu(l-N^Me^_2_S_2_))Cu_2_]^c^ from Henkel *et al.*,^18^ and **14**^a^

	13	[(Cu(l-N^Me^_2_S_2_))Cu_2_]^c^		14
Cu–N	2.2269[6]	2.202[2]	Cu–N	2.046[2]
Cu_eq_–S^e^	2.2563[2]	2.2677[7]	Cu_N2S2_–S	2.2501[5]
Cu(2)···Cu(4)	2.6766(3)	2.726(1)	Cu_ax_–S^i^	2.3686[4]
Cu(1)···Cu(3)	4.4658(4)	4.6251(9)	Cu_ax_–Cl^i^	2.2735[8]
Cu_eq_–Cu_ax_^e^^,^^f^	2.6042[2]	2.6847[4]	Cu_eq_–Cl^j^	2.2178[7]
Cu_ax–S_	2.1910[2]	2.1666[8]	Cu_eq_···Cu_eq_, range^j^	2.7653(7)–3.7676(7)
δ_mean_^g^	0.0341	0.023	Cu(1)–Cu(2)–Cu(3)	119.79(2)^k^
δ_max_^g^	0.0348	0.023	Cu(4)–Cu(5)–Cu(7)	125.28(2)
Cu_eq_–Cu_ax_–Cu_ax_	50.079[3]	59.49[1]	S–Cu_ax_–S, range	94.67(4)–111.80(4)
Cu_ax_–Cu_eq_–Cu_ax_	61.842[6]	61.02[1]	S–Cu_ax_–S, ave	104.79[2]
θ^h^	52.531[6]	51.00	φ^k^	–12.2

a Averaged^d^ values are given where two or more chemically identical interatomic distances or angles are present.

b Data are from unsolvated crystal form (JPD812) only.

c (l-N^Me^_2_S_2_ = *N*^1^,*N*^2^-*bis*(2-mercaptoethane)-*N*^1^,*N*^2^-dimethylpropylenediamine(2−)).

d Uncertainties are propagated according to Taylor, J. R. *An Introduction to Error Analysis*; 2nd ed.; University Science Books: Sausalito, CA, 1997, pp 73–77; propagated uncertainties are designated with [ ].

e The equatorial Cu(I) ions in **13** are Cu(1) and Cu(3).

f The axial Cu(I) ions in **13** are Cu(2) and Cu(4).

g δ = atom deviation (Å) from the Cu_4_ plane.

h θ = average angle between the Cu_2_S planes (“flaps”) and the Cu_4_ plane.

i The axial Cu ions in **14** are Cu(6) and Cu(8).

j The equatorial Cu ions in **14** are Cu(1), Cu(2), Cu(3), Cu(4), Cu5), and Cu(7).

k Torsion angle defined by Cl(4)–Cu(6)–Cu(8)–Cl(5).

Upon treatment with typical S-atom donors such as S_8_, additional thiirane, or Ph_3_SbS, **13** undergoes no observable reaction. It is, however, susceptible to an ill-defined decomposition upon protracted stirring in the open air, which is manifested, *inter alia*, by the onset of a bluish color. Chlorine atom donors such as PhICl_2_ and trityl chloride react with **13** to afford a mixed-valent octacopper cage complex, **14** (Scheme 2 and Figure 3, middle), which is of a form that appears not to have been identified previously with diamino dithiolate ligands. This cage complex is not the result of simple dimerization following oxidation, as **14** contains three diamino *bis*(thiolate) ligands and an odd number of cationic charges (11+). With five Cl^–^ and six thiolate sulfur atoms, the offsetting metal composition necessarily is [(Cu^1+^)_5_(Cu^2+^)_3_]. Although deviating appreciably from a colinear disposition, the Cu(6)–Cl(4) and Cu(8)–Cl(5) bonds define a pseudo *C*_3_ axis. The remaining six copper ions form an equatorial belt between Cu(6) and Cu(8) around which {Cu(l-N_2_(S^Me_2_^)_2_)} and {Cu–Cl} fragments alternate but in an uneven fashion such that, for example, the Cu(1)···Cu(2) interatomic distance is 2.7653(7) Å, while the Cu(1)···Cu(7) separation is 3.6923(8) Å (Figure 3, bottom, and Table 3). This asymmetry further removes the structure from the idealized *C*_3_ point group. The significant shortening by ∼0.2 Å of the Cu–N bonds in **14** as compared to **13** indicates, as the known preference of Cu^2+^ for harder donor atoms would suggest, that the three Cu^2+^ ions are situated within the three l-N_2_(S^Me_2_^)_2_(2−) ligands.

The attenuated basicity of an aryl thiolate vs. alkyl thiolate, in conjunction with a lessened conformational fluidity for diamino dithiolate ligands with arene rings incorporated into the chelate architecture, suggests that *N*^1^,*N*^2^-*bis*(2-mercaptophenyl)-*N*^1^,*N*^2^-dimethylethane-1,2-diamine, **5**, could manifest both different structural chemistry and reactivity in its coordination complexes with copper. The original synthesis of l-N_2_(S^Ar^H)_2_ (**5** in Scheme 1), described by Enemark and co-workers,^26^ employs a sequence of steps beginning with a base-mediated ring-opening hydrolysis of 3-methylbenzothiazolium iodide (**2**, Scheme 1). Following *S*-benzylation, the secondary aniline **3** is subjected to a condensation with glyoxal that is templated with ZnCl_2_ and followed by reduction with sodium cyanoborohydride. While glyoxal is described as accessible in a monomeric, anhydrous form from its cyclic trimer by heating in the presence of P_4_O_10_,^27^ we have found that its generation in useful quantity, free from higher oligomers, is difficult to reproducibly execute. An alternate route to **5** that has been devised by Sellmann and co-workers still demands the use of glyoxal.^28^

**Figure 4:**
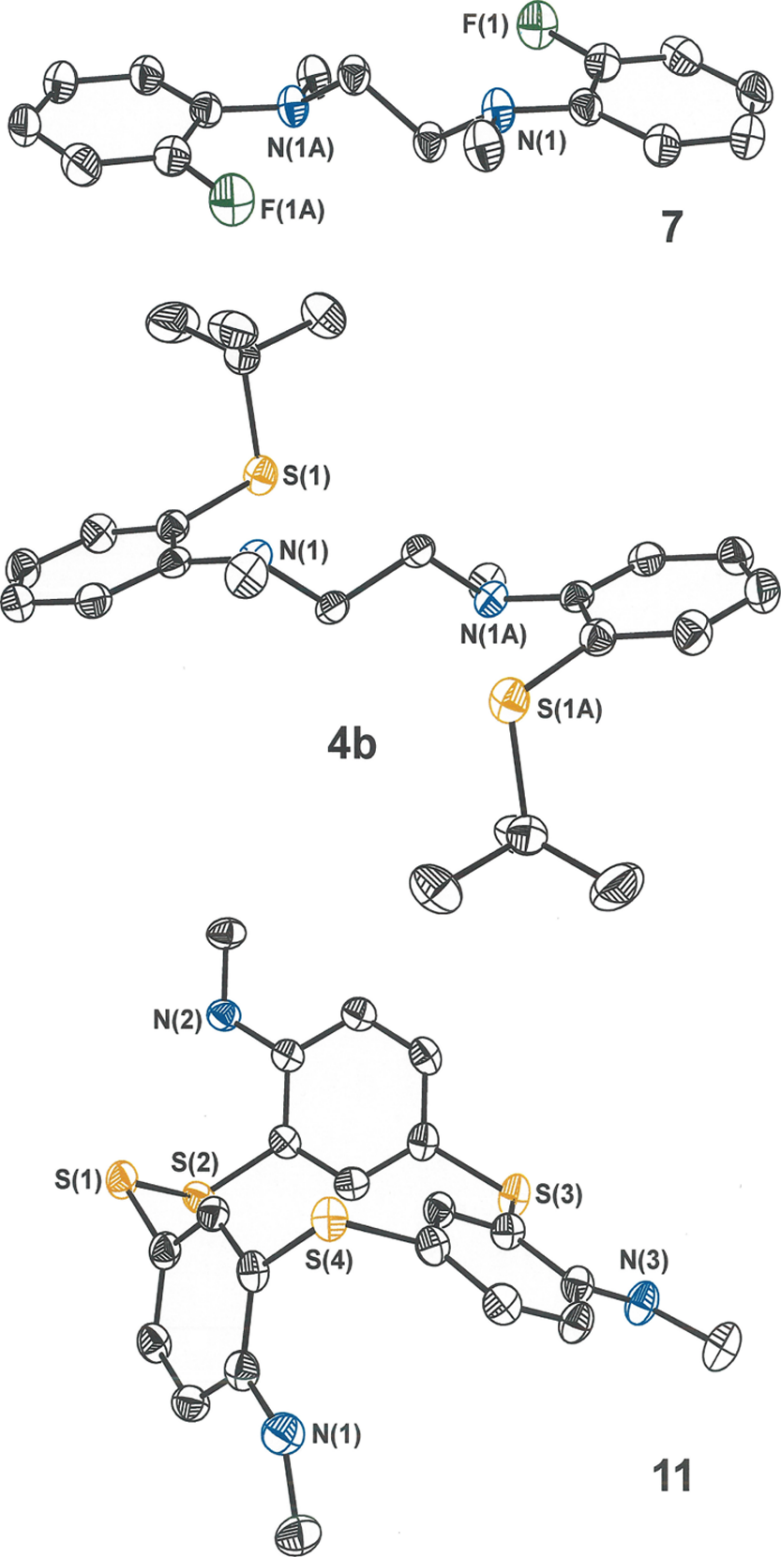
Thermal ellipsoid plots (50%) of ligand precursors **7** and **4b** and of **11** with partial atom labeling. All H atoms are omitted for clarity.

The challenges encountered in reliably producing anhydrous glyoxal prompted our consideration of alternative approaches to **5**. In one approach, disulfide **9** was treated with 1,2-diiodoethane in the presence of base in MeCN at room temperature. Instead of the intended *N*-alkylation and cyclization, a complex product mixture formed from which cyclic **10** and **11** were isolated in minor amounts and identified by X-ray crystallography. As depicted in Scheme 1 and as can be seen in Figure 4, the structures of **10** and **11** reveal a “two down, one up” disposition of arene rings with respect to the plane defined by the sulfur atoms. The structure of **10** is similar to that of **11**. These products likely arise through a sequence that begins as illustrated in Scheme S1 and is enabled by a capacity for the disulfide bond to fragment heterolytically and produce a thiolate leaving group. Being unintended and occurring as a mixture, **10** and **11** were not further characterized.

The commercial availability of 2-fluoro-*N*-methylaniline suggested the possibility that *N*-alkylation to install the ethane backbone might be done prior to functionalization with sulfur. This step is accomplished in moderate yield, affording **7**, but without the need for purification measures beyond several recrystallizations. The substitution of fluoride in **7** can be accomplished with any of several thiolates but demands the use of forcing conditions (refluxing DMF) and protracted reaction times (14–21 d). Yields with isopropylthiolate (**4a**) and *tert*-butylthiolate (**4b**) are comparable (∼90%), but that found with benzylthiolate is substantively lower (∼10%). The crystal structures of **7**, **4a**·2(HCl), **4a**, and **4b** (Figures 4, S1–S4) all show a *trans anti* configuration about the ethane linker between nitrogen atoms such that the carbon–carbon midpoint coincides with a crystallographic inversion center. Consequently, the two tertiary amine nitrogen atoms in **7**, **4a**·2(HCl), **4a**, and **4b** have opposite optical configurations. Deprotection of molecules **4** using Na/NH_3_ is uncomplicated and reproducibly produces diamino *bis*(thiol) **5** in gram quantities with yields of ∼90%.

**Figure 5:**
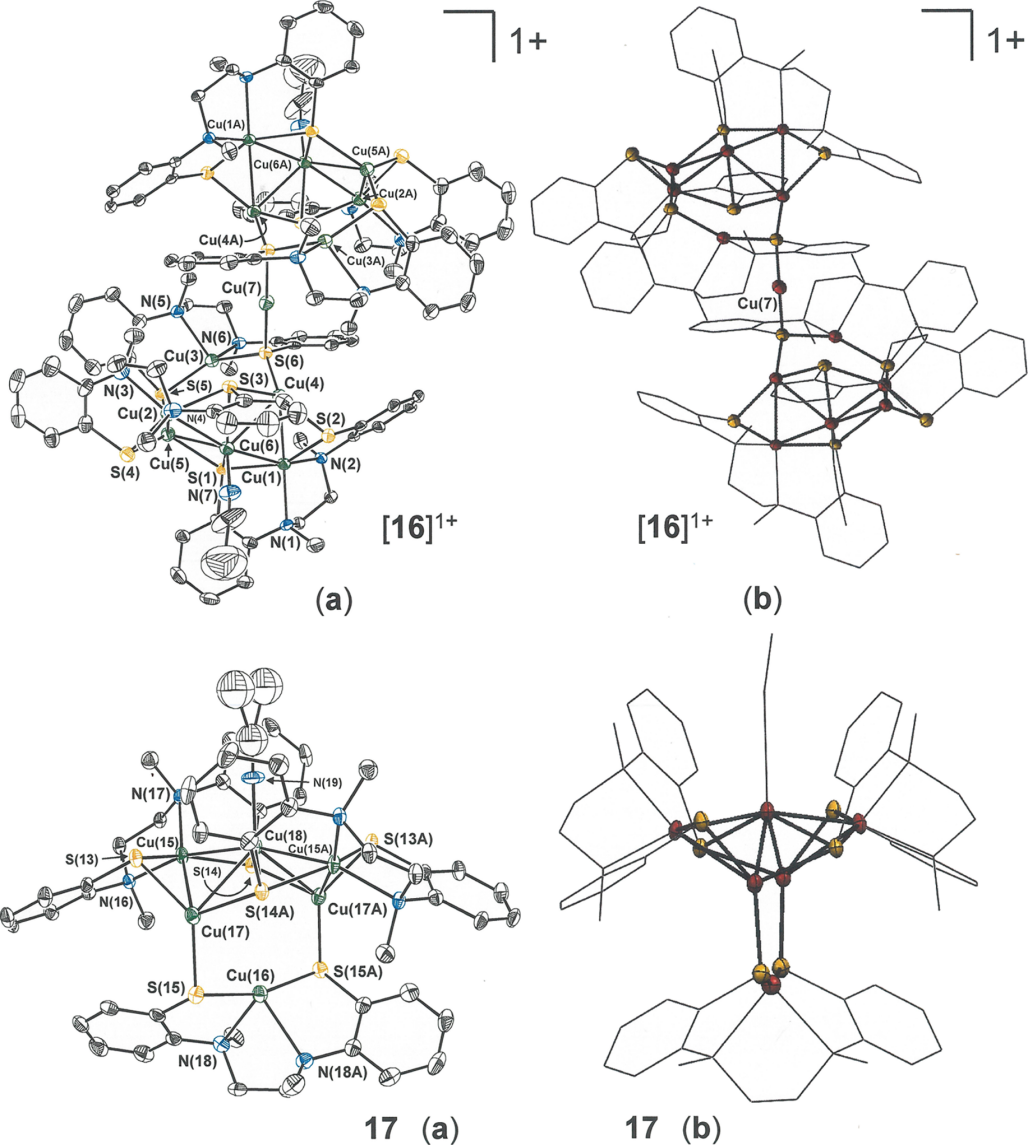
Thermal ellipsoid plots [(**a**), 50%] and mixed wireframe thermal ellipsoid plots [(**b**), 50%] of cation [**16**]^1+^ and charge-neutral cage **17** with partial atom labeling. All H atoms are omitted for clarity. Cation [**16**]^1+^ resides on an inversion center, coincident with Cu(7), while **17** resides upon a *C*_2_ axis passing through Cu(16) and Cu(18). The terminal Me group of the MeCN ligand of **17** is disordered over two positions, both of which are shown in the full thermal ellipsoid plot.

The same reaction conditions that lead to **13**, when applied with ligand **5**, do not produce the analogous tetracopper compound (**15** in Scheme 3) but rather an altogether different cuprous species of exceeding air sensitivity. The all cuprous nature of this compound is punctuated by its colorless appearance. Thin plate crystals of this product could only be characterized by X-ray crystallography, which identified it as a mixture of [Cu_13_]^+^ and Cu_6_ species. The former of these multicopper species, [**16**]^+^ (Figure 5), is a centrosymmetric assembly composed of two Cu_6_ fragment cages that are bridged in a linear fashion by an additional Cu^1+^ ion through S···Cu···S interactions from a [l-N_2_(S^Ar^)_2_]^2–^ ligand on each side. The lone Cu_6_ cage, **17**, is of the same composition and essentially the same structure as the two Cu_6_ moieties that constitute [**16**]^+^ such that [**16**]^+^ can be alternatively formulated as [**[17**]_2_(μ-Cu^I^)]^+^ (Scheme 3). Compound **17** is comprised of three [Cu(l-N_2_(S^Ar^)_2_]^1–^ groups, two Cu^1+^ ions, and a singular [Cu(MeCN)]^1+^ group with resulting overall charge neutrality. The Cu(MeCN) group is coincident with a *C*_2_ axis that bisects the [Cu(l-N_2_(S^Ar^)_2_]^1–^ group (containing Cu(16)) on the cage’s opposite side, thereby rendering only half of **17** structurally unique (Figure 5, bottom, and Scheme 3, bottom). Within this assembly, Cu–N, Cu–S, and Cu···Cu distances vary broadly in an irregular fashion (Table 4).

**Table 4:** Selected Interatomic Distances and Angles for **17** and [**16**]^+^

**17**		**17**	
Cu(15)–S(13)	2.2233(14)	S(15)–Cu(16)–S(15A)	161.80(8)
Cu(15)–S(14)	2.2792(13)	N(16)–Cu(15)–N(17)	85.95(14)
Cu(16)–S(15)	2.1968(12)	N(18)–Cu(16)–N(18A)	82.5(2)
Cu(15)–N(16)	2.138(4)	S(15)–Cu(17)–S(13)	136.00(5)
Cu(15)–N(17)	2.202(4)	S(15)–Cu(17)–S(14A)	108.39(5)
Cu(16)–N(18)	2.333(4)	S(13)–Cu(17)–S(14A)	113.63(5)
Cu(17)–S(13)	2.2549(14)	S(15)–Cu(17)–Cu(15)	123.62(5)
Cu(17)–S(14A)	2.3480(13)	Cu(15)–Cu(18)–Cu(15A)	162.65(5)
Cu(15)–Cu(18)	2.4872(7)	θ_1_^a^	84.8
Cu(15)–Cu(17)	2.6361(10)	θ_2_^b^	90.1
Cu(17)–Cu(18)	2.7080(9)	**[16]**^**+**^	
Cu(17)···Cu(17A)	4.209	S(6A)–Cu(7)–S(6)	180
S(13)–Cu(15)–S(14)	147.23(5)	Cu(7)–S(6)	2.1549(12)

a Angle between intraligand S(13)–C_6_–N(16) and S(14)–C_6_–N(17) mean planes.

b Angle between intraligand S(15)–C_6_–N(18) and S(15A)–C_6_–N(18A) mean planes.

**Figure 6:**
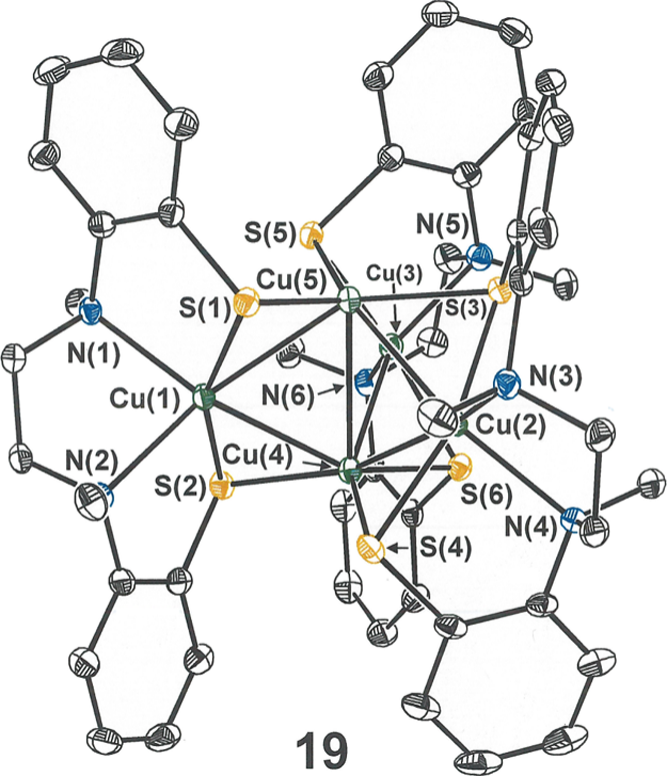
Thermal ellipsoid plot (50%) of the pentacopper compound **19** with partial atom labeling. All H atoms are omitted for clarity.

Upon even slight exposure to air or to handling in CH_2_Cl_2_, compound [**16**]^1+^/**17** undergoes immediate oxidation, first manifesting a blue color but ultimately transforming to a mixed-valent red-brown species of a notably robust characteristic. This new cage compound, **19**, is quite tractable to crystallization and yields a variety of polymorphs/pseudopolymorphs (Tables 2 and S4) that are the same in all essential respects. As revealed by X-ray crystallography (Figure 6), **19** has a charge-neutral formulation with a *penta*copper core displaying a distinctive 3-fold axial symmetry. The conversion of **17** to **19** requires only a one-electron oxidation and extrusion of the [Cu(MeCN)]^+^ fragment. Two of the copper ions [Cu(4) and Cu(5) in Figure 6] define a central axis around which three Cu[l-N_2_(S^Ar^)_2_] groups are arrayed with a modestly twisted disposition. Relative to the central Cu_2_ axis, the intrachelate S···S line segment of each [l-N_2_(S^Ar^)_2_]^2–^ ligand is inclined at a ∼−33° torsion angle. In contrast to the free l-N_2_S_2_ ligands and their precursors, which typically crystallize with opposing chiral configuration at each tertiary nitrogen atom, the handedness at each amine nitrogen in l-N_2_S_2_ coordination complexes is invariably the same because this arrangement better accommodates the lower energy puckered chelate ring configuration. Thus, each individual [Cu(l-N_2_S^Ar^_2_)] fragment in **19** conforms to *C*_2_ local symmetry with the rotational axis bisecting the S–Cu–S and N–Cu–N angles. Taken together as a set, three [Cu(l-N_2_S^Ar^_2_)] fragment complexes of the same optical configuration trigonally arranged around a central axis present idealized *D*_3_ point group symmetry. Structural integrity is maintained in solution, as demonstrated by ^1^H and ^13^C NMR spectra that reveal half of a l-N_2_S^Ar^_2_(2−) ligand to be unique and mass spectrometric data that show only the parent ion with no fragmentation. In one of the polymorphs identified (*cf*. Figure S22), **19** occurs on a crystallographic *C*_2_ that coincides with a molecular *C*_2_; in all other instances, **19** is found on a general position. If the handedness of **19** as a whole is defined by viewing down the *C*_3_ axis and considering whether each thiolate sulfur of the triangular S3 face has the connectivity leading to its intraligand partner sulfur atom emanating to the left (Λ) or right (Δ), Figure 6 shows the Δ isomer. Because all the polymorphs/pseudopolymorphs of **19** that have been found occur in centric space groups (*P*-1 or *C*2/*c*), they are necessarily racemic mixtures in these crystalline forms.

**Figure 7:**
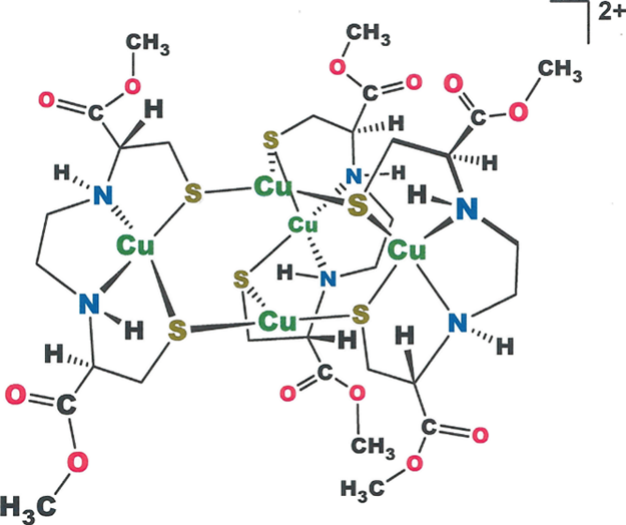
A 3-fold symmetric dicationic pentacopper compound reported by Schugar and co-workers.^29^

The core of **19** is quite compact with a Cu_ax_···Cu_ax_ separation of 2.7005(4) Å and an average Cu_ax_···Cu_eq_ distance of 2.6205[2] Å that define a trigonal bipyramidal Cu_5_ core (Table 5). The Cu(17)···C(17A) separation of 4.209 Å in **17** (Figure 5), which condenses to define the *C*_3_ axis in **19**, accentuates the close packing within the latter. Although the observation of [**16**]^1+^ and **17** as initial products, at least when MeCN is implemented as a solvent, suggests that the intended **15** (Scheme 3) is not formed, **19** could be created, at least as a formalism, from the addition of [Cu^II^(l-N_2_S^Ar^_2_)] to the Cu_2_ axis of **15**. The Cu–N bond lengths in **19** are modestly shorter than in **13** (Tables 5*vs*3), in contrast to the significant Cu–N contraction in **14***vs***13**, which is consistent with the single cupric ion being disordered among the three equatorial [Cu(l-N_2_S^Ar^_2_)] sites. An intriguing point regarding **19** is its relationship to a trigonally symmetric Cu_5_ compound reported by Schugar and co-workers (Figure 7),^29^ which was structurally characterized as a *dication* featuring an expanded core that is quantified most simply by its Cu_ax_···Cu_ax_ separation of 3.016(3) Å. The two-electron difference between **19** and Schugar’s compound suggests that both cage species might support 2e^–^ redox chemistry under the appropriate conditions.

**Table 5:** Selected Interatomic Distances (Å) and Angles (deg.) for **19**^a^^,^^b^

Cu_ax_–Cu_ax_	2.7005(4)	C–C_arene_	1.403[1]
Cu_ax_–Cu_eq_, ave	2.6205[2]	S–Cu_N2S2_–S	158.42[1]
Cu_ax_–Cu_eq_, range	2.5292(4)–2.6725(4)	S–Cu_ax_–S	116.57[1]
Cu_eq_–Cu_eq_, ave	3.8890[2]	N–Cu_N2S2_–N	85.67[3]
Cu_eq_–Cu_eq_, range	3.8336(4)–3.9389(4)	θ_1_^c^	89.7
Cu_N2S2_–S	2.2346[2]	θ_2_^d^	83.1
Cu_N2S2_–N	2.2143[7]	θ_3_^e^	83.4
S–C	1.780[1]		

a Data are for the *C*2/*c* polymorph with **19** on general position (JPD722).

b Averaged values are given where two or more chemically identical interatomic distances or angles are present. Uncertainties are propagated according to Taylor, J. R. *An Introduction to Error Analysis*; 2nd ed.; University Science Books: Sausalito, CA, 1997, pp 73–77; propagated uncertainties are designated with [ ].

c Angle between intraligand S(1)–C_6_–N(1) and S(2)–C_6_–N(2) mean planes.

d Angle between intraligand S(3)–C_6_–N(3) and S(4)–C_6_–N(4) mean planes.

e Angle between intraligand S(5)–C_6_–N(5) and S(6)–C_6_–N(6) mean planes.

Ligand **5**, when introduced to any of a variety of Cu^II^ sources, again yielded **19** as the only identifiable product rather than mononuclear [Cu^II^(l-N_2_S^Ar^_2_)] (Scheme 3), the ligand probably serving as the source of reducing equivalents that enables this result.^30^ When treated with [Cp_2_Fe][PF_6_] in MeCN, **19** underwent a striking change in color from red-brown to intense violet. As with **19** itself, only half of each l-N_2_(S^Ar^)_2_(2−) ligand is spectroscopically distinct by ^1^H NMR (Figure S57), suggesting that, in solution, this oxidized product is [**19**]^+^ with preservation of the *D*_3_ symmetry. The cyclic voltammetry behavior of **19** (*vide infra*) is also consistent with retention of its pentacopper formulation upon a full, stoichiometric one-electron oxidation.

**Figure 8:**
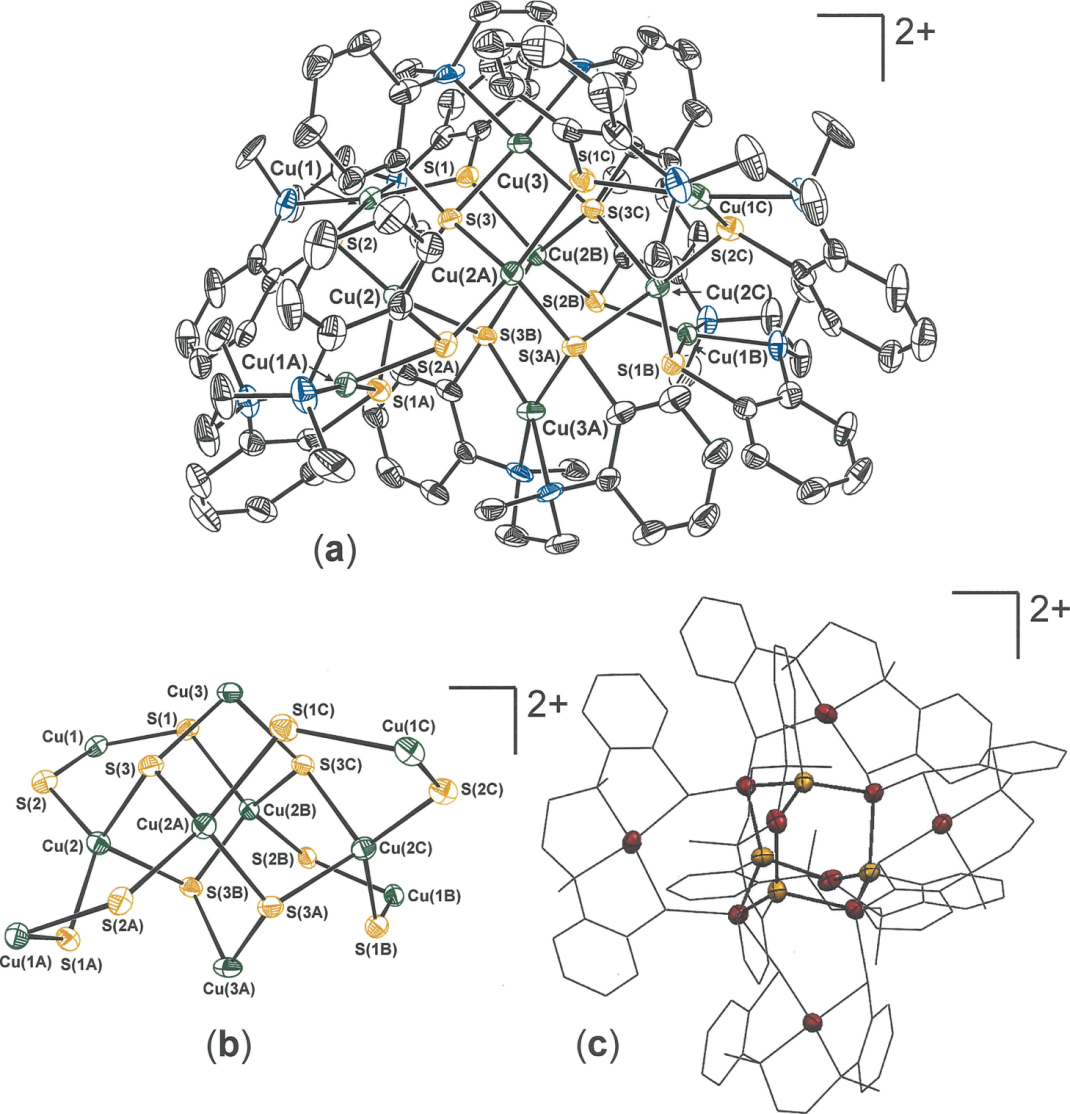
(a) Thermal ellipsoid plot (50%) of dication [**20**]^2+^ with partial atom labeling. All H atoms are omitted for clarity. (b) Core topology of [**20**]^2+^ defined by the Cu and S atoms and (c) mixed wireframe/thermal ellipsoid (50%) image of [**20**]^2+^ revealing the central Cu_6_S_4_ adamantanoid cage defined by Cu(3) and Cu(3A), with the sulfur atoms of their l-N_2_S^Ar^_2_(2−) ligands, and Cu(2A)–Cu(2D).

Dark wedge-shaped crystals grown by the diffusion of Et_2_O into a MeCN solution of [**19**]^+^ were interrogated by X-ray diffraction and identified as the decacopper dication shown as [**20**]^2+^ (Scheme 3 and Figure 8). Considered as a simple matter of composition, [**20**]^2+^ is a dimer of [**19**]^1+^. However, the structure of [**20**]^2+^ does not admit of simple description in relation to **19**, suggesting that, while the redox chemistry may be rapid, there is an attending structural reorganization on a slower timescale. The trigonal symmetry of **19** is altogether lost and is not observed even as localized symmetry in fragments of [**20**]^2+^. Rather, [**20**]^2+^ occurs in tetragonal *I*4_1_/*a* (no. 88) on an *S*_4_ axis that is defined by ions Cu(3) and Cu(3A) (vertical direction, Figure 8, top, and Scheme 3, bottom right). The remaining copper ions comprise two sets of four ions [Cu(2)–Cu(2c) and Cu(1)–Cu(1c); *cf*., Figure 8b], the members of each set being related by successive executions of the *S*_4_ operation. The *S*_4_ point group demands that the [Cu(l-N_2_S^Ar^_2_)] fragments containing Cu(3) and Cu(3A) at the top and bottom of the cage assembly, as presented in Figure 8 (top), have opposite *C*_2_ handedness. Similarly, the [Cu(l-N_2_S^Ar^_2_)] groups holding Cu(1)–Cu(1C) alternate in their isolated *C*_2_ chirality. The central cavity of [**20**]^2+^ features an Cu_6_S_4_ adamantanoid-like environment created by the four Cu ions not encapsulated by l-N_2_S^Ar^_2_(2−) ligands [Cu(2A)–Cu(2D)], the two Cu ions that coincide with the *S*_4_ axis [Cu(3) and Cu(3A)], and the four sulfur atoms of the two ligands chelating Cu(3) and Cu(3A) [S(3)–S(3C)]. A related decacopper cage compound bearing the formulation [(Cu^II^(l-N_2_S_2_))_4_(μ_2_-Cu^I^(MeCN)_2_)_2_(μ_3_-Cu^I^(MeCN))_4_]^6+^, where l-N_2_S_2_ = *N*,*N*′-dimethyl-*N*,*N*′-*bis*(2-mercaptoethyl)ethylenediamine(2−), is similarly of S_4_ symmetry but differs in having four [Cu(l-N_2_S_2_)] fragments rather than six as in [**20**]^2+^.^31^ A striking contrast between **19** and [**20**]^2+^ is the near orthogonality of the intraligand aminothiolate rings in the former (θ, Table 5) and their near planarity in the latter (θ, Table 6).

**Table 6:** Selected Interatomic Distances (Å) and Angles (deg.) for [**20**]^2+^^a^

Cu_N2S2_–N	2.042[6]	Cu(2)···Cu(2A)	3.898[2]
Cu_N2S2_–S	2.260[2]	Cu(1)···Cu(1C)	9.749(2)
Cu_S4_–S	2.327[2]	θ_1_^c^	8.9
S–C	1.788[8]	θ_2_^d^	8.4
Cu(3)···Cu(3A)	5.541	τ_1_^e^	22.1°
		τ_2_^f^	31.5°

a Averaged^b^ values are given where two or more chemically identical interatomic distances or angles are present.

b Uncertainties are propagated according to Taylor, J. R. *An Introduction to Error Analysis*; 2nd ed.; University Science Books: Sausalito, CA, 1997, pp 73–77; propagated uncertainties are designated with [ ].

c Angle between intraligand S(1)–C_6_–N(1) and S(2)–C_6_–N(2) mean planes.

d Angle between intraligand S(3)–C_6_–N(3) and S(3C)–C_6_–N(3C) mean planes.

e Angle between “cis”-disposed CuN_2_S_2_ mean planes for [Cu(l-N_2_S^Ar^_2_)] fragments not bisected by the *S*_4_ axis.

f Angle between “trans” disposed CuN_2_S_2_ mean planes for [Cu(l-N_2_S^Ar^_2_)] fragments not bisected by the *S*_4_ axis.

**Figure 9:**
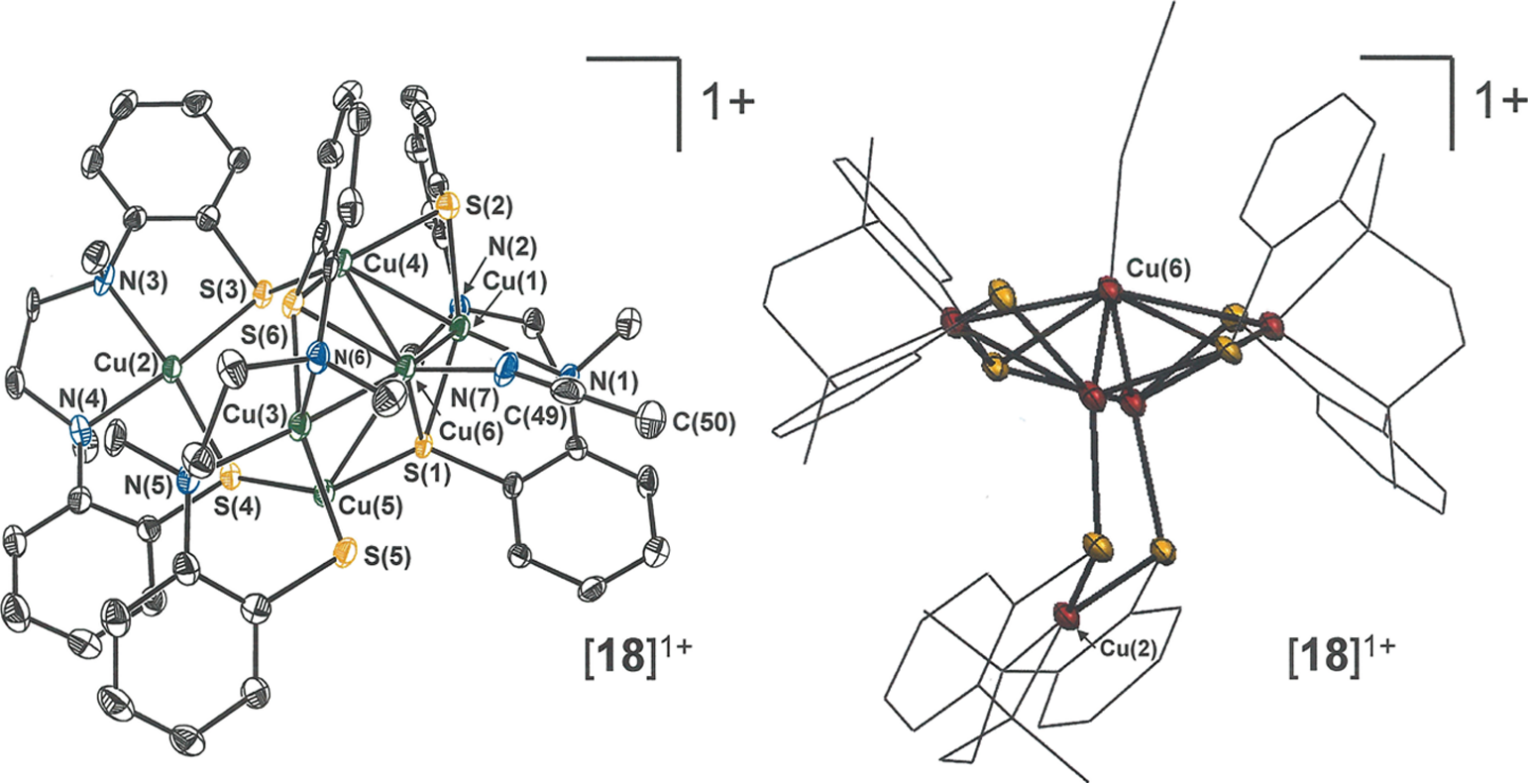
Thermal ellipsoid plot (50%) of cation [**18**]^+^ with all H atoms removed for clarity (left). Mixed wireframe thermal ellipsoid (50%) image of [**18**]^+^ with the Cu(2)···C(6) axis in the vertical direction rather than horizontal (right).

**Scheme 4:**
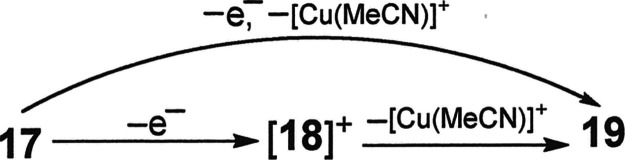
Interconversions Relating **17**, [**18**]^+^, and **19**

When [Cu(MeCN)_4_][PF_6_] is treated first with [**5**]^2–^ in THF and then, in a subsequent step, 1/4 equiv of Ph_3_SbS is administered, the intended oxidative addition of sulfur does not occur but rather a presumed one-electron oxidation of *in situ* generated **17** to afford [**18**]^+^ (Scheme 3). The mixed-valence constitution of [**18**]^+^ confers upon it a marked stability that contrasts sharply with the air sensitivity of fully reduced **17**. While, as a matter of composition, [**18**]^+^ is a one-electron oxidized form of **17**, its structure departs from the *C*_2_ symmetry of the latter by a moderate twisting of one [Cu(l-N_2_(S^Ar^)_2_)] fragment with respect to the others. Consequently, the overall appearance of [**18**]^+^ bears a closer relationship to **19** than to **17**, as it in principle could arise by the addition of [Cu(MeCN)]^+^ to an equatorial edge of the Cu_5_ trigonal bipyramid of **19**, the *C*_3_ axis of which is defined by the Cu(4)···Cu(5) segment (*cf*. Figures 6 and 9 and Scheme 3). In Figure 9, atoms Cu(6)–N(7)–C(49)–C(50) define this added group, while the Cu(3)···Cu(1) segment marks the edge of the Cu_3_ equatorial belt to which it has been joined. Selected interatomic distances and angles for [**18**]^+^ are collected in Table 7. Scheme 4 summarizes in abbreviated form the formal stoichiometric relationships between **17**, [**18**]^+^, and **19**, but the interconversions involving the latter two have not been proven by deliberate synthesis starting from pure, isolated samples.

**Table 7:** Selected Interatomic Distances and Angles for **[18]**^+^

Cu(1)–S(1)	2.2419(17)	Cu(3)–N(5)	2.201(5)
Cu(1)–S(2)	2.2238(18)	Cu(3)–N(6)	2.193(5)
Cu(2)–S(3)	2.2248(17)	Cu(1)–Cu(6)	2.6093(11)
Cu(2)–S(4)	2.2433(18)	Cu(3)–Cu(6)	2.5896(11)
Cu(3)–S(5)	2.2440(18)	Cu(4)–Cu(6)	2.5670(11)
Cu(3)–S(6)	2.2612(17)	Cu(5)–Cu(6)	2.6434(12)
Cu(1)–N(1)	2.202(5)	Cu(4)···Cu(5)	4.1002(13)
Cu(1)–N(2)	2.174(5)	S(2)–Cu(1)–S(1)	148.20(7)
Cu(2)–N(3)	2.036(5)	S(3)–Cu(2)–S(4)	102.46(6)
Cu(2)–N(4)	2.033(5)	S(5)–Cu(3)–S(6)	144.20(7)

### Spectra and Electrochemistry

**Figure 10:**
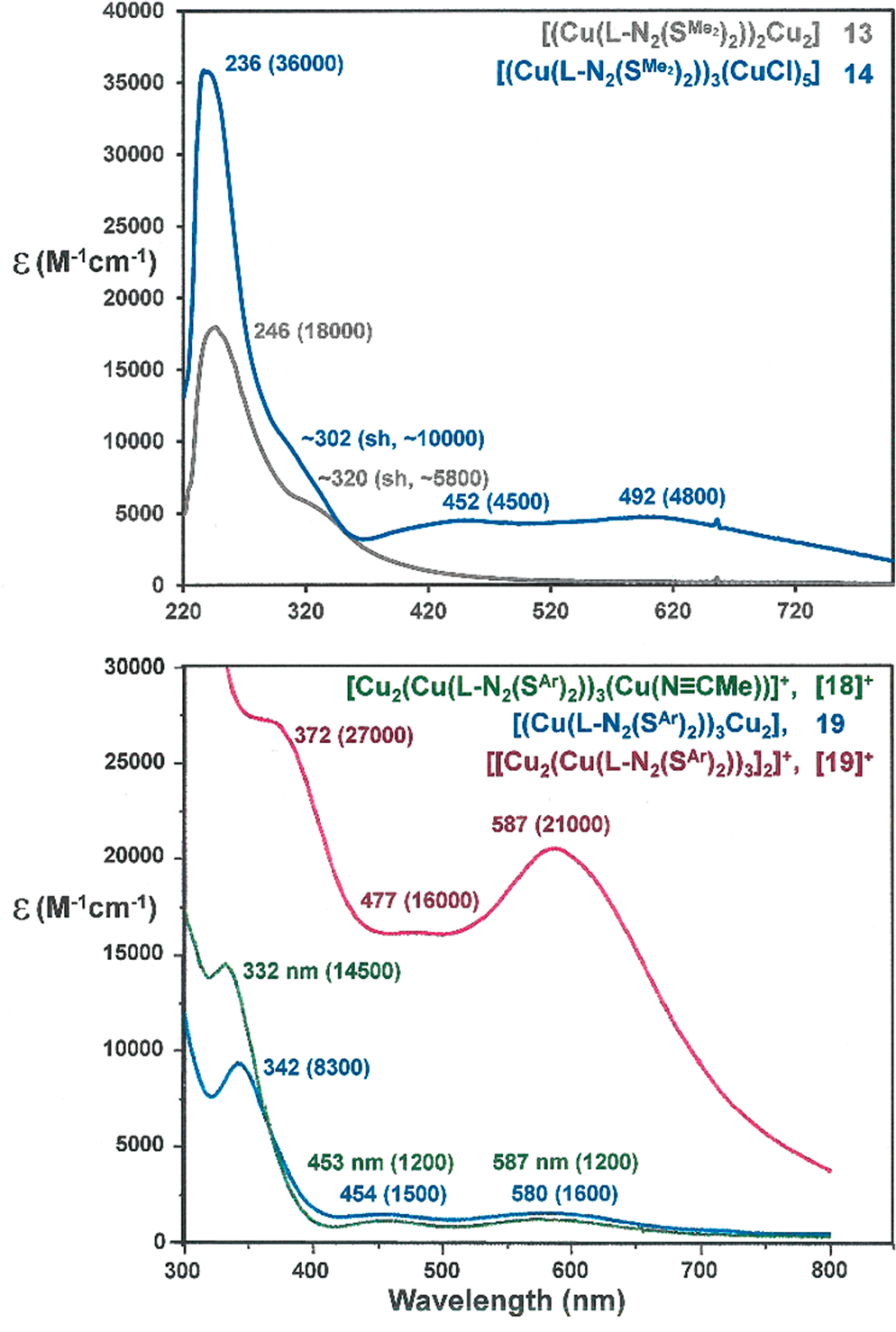
Overlaid UV–vis spectra for **13** and **14**, with [l-N_2_(S^Me_2_^)_2_]^2–^ (top), and for [**18**]^+^, **19**, and [**19**]^+^, with [l-N_2_(S^Ar^)_2_]^2–^ (bottom). All spectra have been acquired using solutions in CH_2_Cl_2_.

Compounds **13** and [**16**]^+^/**17**, being of an all cuprous formulation, are diamagnetic and without any informative features in the electronic absorption spectrum. Compounds **19**, **14**, and [**19**]^+^, however, are distinctively colored (red-brown, blue, and violet, respectively) with higher intensities and lower energies that correlate with increasing ratio of Cu^II^/Cu^I^ (Figure 10). The electronic absorption spectra of the pentacopper cation in Figure 7 and related multicopper complexes with l-N_2_S_2_(2−) ligands have been analyzed in some detail by Schugar, Potenza, and co-workers and attributed to a complex overlay of Cu^I^ → Cu^II^ MMCT, Sπ/Sσ → Cu^II^ LMCT, and Cu^I^ → S MLCT transitions.^32^ Considering the compositional similarities that **14**, **19**, and [**19**]^+^ share with these compounds, analogous assignments are undoubtedly pertinent to them, although transitions involving Sπ charge transfer are likely energy-shifted due to the influence of the arene ring. Rigorous spectral deconvolutions and computationally assisted assignments have not been attempted here.

**Figure 11:**
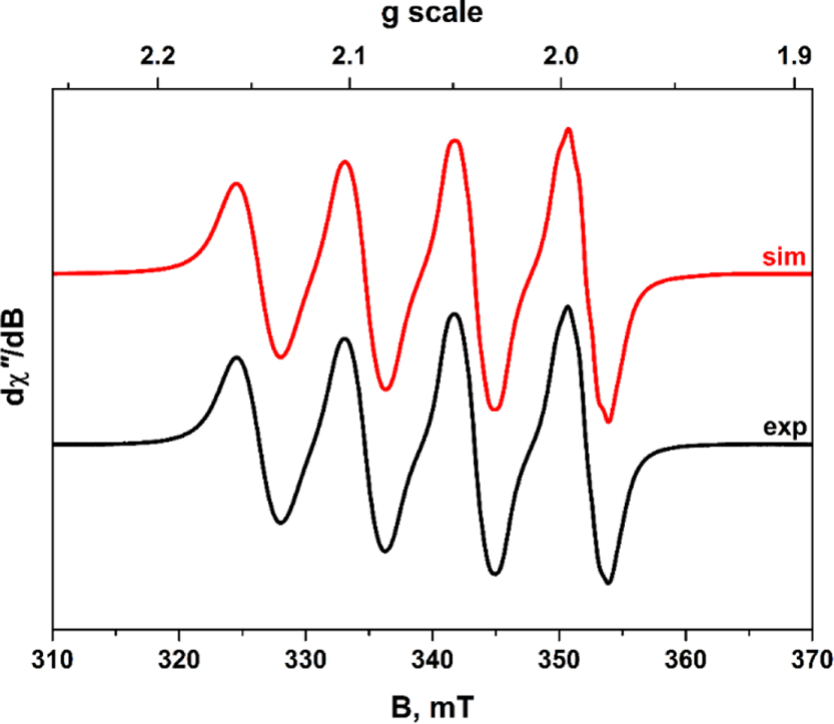
X-band EPR spectrum of **19** recorded in CH_2_Cl_2_ solution at 293 K (experimental conditions: frequency, 9.8017 GHz; power, 63 mW; and modulation, 0.1 mT). Experimental data are represented by the black line; simulation is depicted by the red trace. Simulation parameters: *g*_iso_ = 2.0633; *A*_iso_{^63,65^Cu} = 81.4 × 10^–4^ cm^–1^ (1); and *A*_iso_{^14^N} = 8.3 × 10^–4^ cm^–1^ (2).

The room-temperature X-band EPR spectrum of **19** in CH_2_Cl_2_ reveals the quartet signal of a single spin at ^63^Cu/^65^Cu with hyperfine coupling to ^14^N (Figure 11), thus indicating that the Cu^II^ ion is situated within a l-N_2_(S^Ar^)_2_(2−) chelate and is not, considering the close 2.7005(4) Å Cu_ax_···Cu_ax_ contact, delocalized along the central Cu_2_ core axis. Because the ESI mass spectrum of **19** reveals no fragmentation peaks between 330 amu and the parent mass at ∼1225 amu (Figure S60), attribution of this signal to intact **19**, as opposed to mononuclear [Cu^II^(l-N_2_(S^Ar^)_2_] arising by facile disassembly, stands as a secure interpretation. Although **19** was not tractable to a straightforward gas-phase geometry optimization, possibly because its energy surface is comprised of multiple shallow minima without a decisive global minimum, a single-point calculation performed using the crystallographic coordinates and a subsequent spin density plot affirm that the unpaired spin is largely localized at a single copper site (Figure S63).

**Figure 12:**
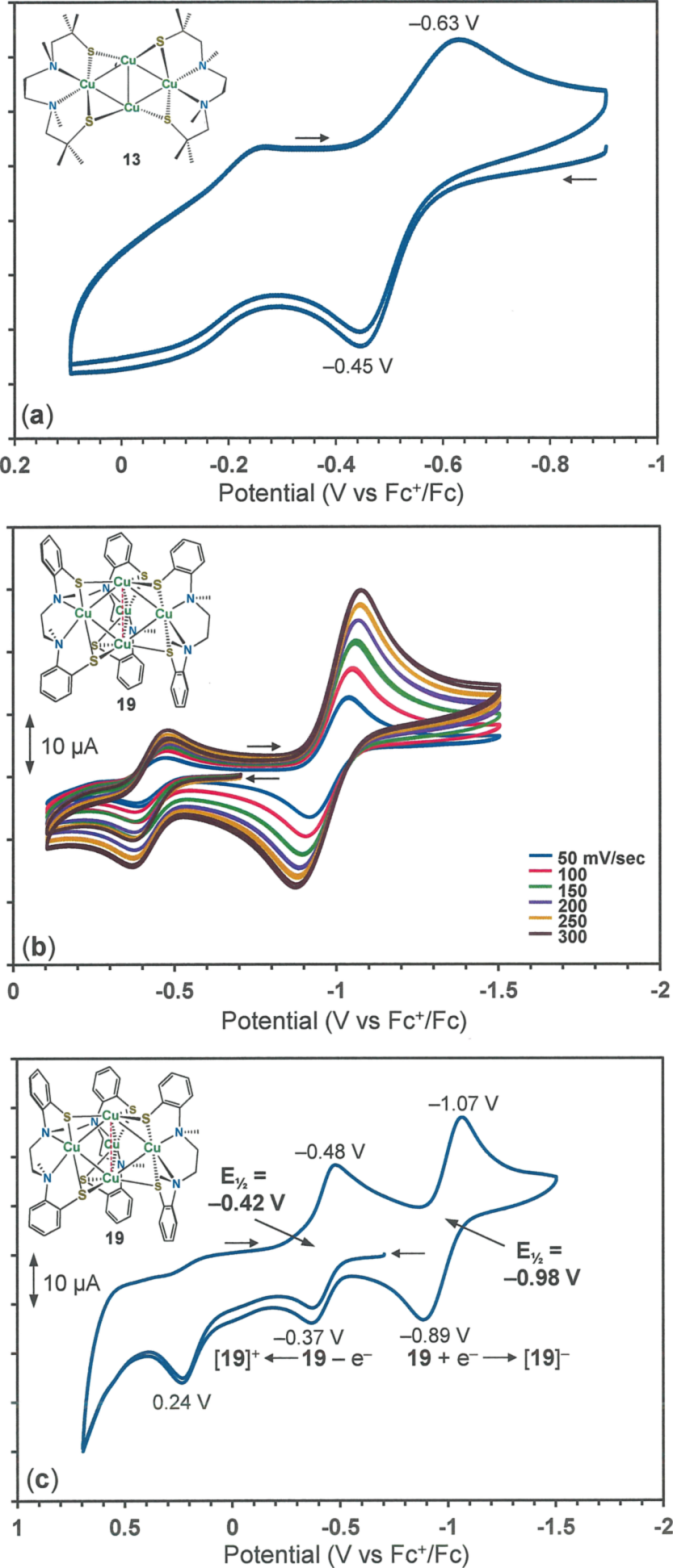
Cyclic voltammograms of **13** (a), **19** at 300 mV/sec (b), and **19** at variable scan speed (c) in CH_2_Cl_2_ with [^*n*^Bu_4_N][PF_6_] supporting electrolyte.

Owing to its fully reduced formulation, **13** reveals only a quasi-reversible anodic wave at ∼−0.54 V Fc^+^/Fc (Figure 12a), followed by an irreversible process at ∼−0.15 V. The lack of reversibility possibly has its basis in facile, out-of-plane movement of one of the [Cu(N_2_S^Me_2_^_2_)] fragments from the remaining cuprous core. Compound **14** shows only irreversible behavior in its voltammogram, which is unsurprising in view of the irregularity of its core structure and the structural fluidity that it implies. This observation contrasts sharply with that of the hexacopper complex [(bme*daco)Cu]_2_(μ-CuCl)_4_] (bme*daco = *bis*(*N*,*N*′-2-mercapto-2-methylpropyl)-1,5-diazocyclooctane) described by Darensbourg, which undergoes two reversible Cu^II^ + e^–^ → Cu^I^ reductions and two reversible Cu^II^ – e^–^ → Cu^III^ oxidations.^33^ The greater structural stability of this compound’s adamantanoid-like topology undoubtedly underpins this markedly different behavior.

**Scheme 5:**
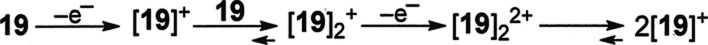
Proposed Oxidation Processes and Solution Equilibria for **19**

The cyclic voltammogram of **19** reveals a reversible reduction at −0.98 V *vs* Fc^+^/Fc and an oxidation wave of smaller current amplitude at −0.42 V (Figure 12b). When scanning within the same parameters is initiated in the cathodic direction, rather than the anodic direction as in Figure 12b, these two features maintain their reversible appearance and relative intensity (Figure S64). Both the reduction and oxidation processes are metal-based, corresponding to Cu^II^ + e^–^ → Cu^I^ and Cu^I^ – e^–^ → Cu^II^, respectively, as confirmed by a single-point calculation using the crystal coordinates of **19** and an inspection of its frontier MOs. The current amplitude for the reduction is approximately twice that of the oxidation process, provided that the anodic scanning does not go beyond ∼−0.1 V *vs* Fc^+^/Fc. Continued oxidative scanning beyond this threshold is marked by an irreversible wave with current maximum at +0.24 V and the restoration of current intensity to the initial oxidation wave such that it becomes comparable in amplitude to the reduction process (Figure 12c). As hinted by the identification of [**20**]^2+^ (a dimerized oxidized form of **19**) in the crystalline state, the smaller current for the oxidation wave in Figure 12b may have its basis in a rapid dimerization between [**19**]^+^ and neutral **19**, which depletes the concentration of **19** at the diffusion layer and diminishes the current in comparison to the cathodic wave (Scheme 5). If continued anodic scanning generates [**19**]_2_^2+^, which undergoes facile fragmentation to an equilibrium favoring 2[**19**]^+^ because of charge repulsion (Scheme 5), then the concentration of [**19**]^+^ at the diffusion layer is returned to a value similar to that which it would have had in the absence of a competing dimerization, and the current amplitude of the first oxidation is reconstituted to a scale similar to the reduction wave. The electrochemistry of [**20**]^2+^ was not investigated but likely does not relate simply to that of **19** because of the appreciable structural reorganization that separates them.

Reaction of *in situ* generated [**19**]^1-^ with S_8_ was examined as a means toward a Cu_3_^I^Cu_2_^II^(μ_5_-S) species that would have relevance to the oxidized Cu_Z_* site. However, rapid reoxidation of [**19**]^1–^ back to **19** was the observed outcome, indicating that a fast, outer-sphere electron transfer is kinetically much more competitive than sulfur atom addition regardless of what thermodynamic favorability an expanded Cu_3_^I^Cu_2_^II^(μ_5_-S) cage might enjoy.

## Summary and Conclusions

The principal findings of this report are as follows:

(1) A newly designed, reproducible synthesis of *N*^1^,*N*^2^-*bis*(2-mercaptophenyl)-*N*^1^,*N*^2^-dimethylethane-1,2-diamine (l-N_2_(S^Ar^H)_2_) is described, which proceeds through the key intermediate *N*^1^,*N*^2^-*bis*(2-fluorophenyl)-*N*^1^,*N*^2^-dimethylethane-1,2-diamine and avoids the use of anhydrous glyoxal.
(2) The diamino *bis*(thiolate) ligand *N*^1^,*N*^2^-*bis*(2-methyl-2-mercaptopropane)-*N*^1^,*N*^2^-dimethylethane-1,2-diamine) (l-N_2_(S^Me_2_^)_2_(2−)) supports the formation of the all-cuprous tetracopper compound [(Cu(l-N_2_(S^Me_2_^)_2_))_2_(μ-Cu_2_)], which was targeted as a synthon toward a Cu_2_^I^Cu_2_^II^(μ-S) species relevant to the Cu_Z_/Cu_Z_* site of nitrous oxide reductase.
(3) Tetracopper [(Cu(l-N_2_(S^Me_2_^)_2_))_2_(μ-Cu_2_)] does not undergo well-defined sulfur atom addition but rather chlorine atom addition from PhICl_2_ or Ph_3_CCl to a new, mixed-valent octacopper species, [(Cu(l-N_2_(S^Me_2_^)_2_))_3_(CuCl)_5_].
(4) Introduction of Cu(I) sources to l-N_2_(S^Ar^H)_2_ or its deprotonated form in MeCN leads initially to a highly air-sensitive *C*_2_-symmetric hexacuprous species, identified crystallographically as being composed of 3 [Cu^I^(l-N_2_(S^Ar^)_2_] fragments and three additional Cu(I) ions, one of which is bound to a MeCN ligand. Upon exposure to air, this hexacuprous compound undergoes rapid transformation to a mixed-valent *penta*copper [(Cu(l-N_2_(S^Ar^)_2_))_3_(μ-Cu_2_)], where the single cupric ion is ensconced within one of the l-N_2_(S^Ar^)_2_(2−) ligands, as demonstrated by EPR.
(5) Pentacopper [(Cu(l-N_2_(S^Ar^)_2_))_3_(μ-Cu_2_)] shows both reversible reduction and reversible oxidation by cyclic voltammetry. Chemical generation of [(Cu(l-N_2_(S^Ar^)_2_))_3_(μ-Cu_2_)]^1–^ followed by reaction with S_8_ returns only the neutral starting compound; chemical oxidation with [Cp_2_Fe][PF_6_] results in [(Cu(l-N_2_(S^Ar^)_2_))_3_(μ-Cu_2_)]^+^ in solution, which features intense absorptions in its electronic spectrum, but a dimerized dicationic decacopper aggregate is identified in the crystalline state.

We conclude from this work that diamino dithiolate tetradentate complexes of copper are not well suited to accommodate bridging sulfide by oxidative addition and that, if this ligand platform is compatible with a sulfide-bridged multicopper core, the μ_4_-S ligand must be present first in an appropriate precursor. In multicopper aggregates of the form [[Cu(l-N_2_S_2_)]_*x*_Cu_*y*_^I^]^*n*^, such redox chemistry as they can support generally appears to be restricted to Cu^II^ + e^–^ ↔ Cu^I^ and Cu^II^ – e^–^ ↔ Cu^III^ processes by the ions installed within the [l-N_2_S_2_]^2–^ ligands, while the Cu^I^ ions exogenous to these ligands adjust structurally with their accommodating coordination sphere numbers and geometries as dictated by the exigencies of sterics, charge accumulation, *etc*. We suspect that our observations reflect kinetic effects rather than a thermodynamic impossibility of forming a Cu_4_(μ_4_-S) compound with diamino dithiolate ligands. In continuing work, we are evaluating the coordination chemistry of Cu(I) with new polyimidazole ligands, the aim of which is generation of compounds with compositional and reactivity relevance to Cu_Z_/Cu_Z_*.

## Supporting Information Available

Procedures for crystal growth, X-ray diffraction data collection, and structure solution and refinement; description of computational procedures; unit cell and refinement data; thermal ellipsoid plots with complete atom labeling; and analytical, spectroscopic, and electrochemical data for compounds reported (PDF)
