# Supplementary material for: Fully Reduced and Mixed-Valent Multi-Copper Aggregates Supported by Tetradentate Diamino Bis(thiolate) Ligands
Source: Inorg Chem. 2023 Jun 13;62(25):9854–71. doi: 10.1021/acs.inorgchem.3c00784 (PMC10302893; doi:10.1021/acs.inorgchem.3c00784)
Supplement: Supplementary file 1 — ic3c00784_si_001.pdf [file ic3c00784_si_001.pdf]

Supporting Information

for

Fully Reduced and Mixed-Valent Multi-Copper Aggregates Supported by Tetradentate Diamino Bis(thiolate) Ligands

Bo Wang,† Justin Barnes,† Skylar J. Ferrara,† Stephen Sproules,‡ Xiaodong Zhang,† Joel T. Mague,† and James P. Donahue†,*

†Department of Chemistry, Tulane University, 6400 Freret Street, New Orleans, LA 70118-5638.
‡WestCHEM, School of Chemistry, University of Glasgow, Glasgow, G12 8QQ, United Kingdom.
*Author to whom correspondence may be addressed.

Table of Contents

**Procedures for Crystal Growth, Collection and Processing of Diffraction Data, and Solving and Refining of Structures**.		S4-S6
**Computational Procedures**.		S7
**Table S1**.	Unit cell and refinement data compounds **4b**, **4c**, [**4a**·H_2]Cl_2, and **7**.	S8
**Table S2**.	Unit cell and refinement data for compounds **9**, **10**, and **11**.	S9
**Table S3**.	Unit cell and refinement data for compounds **13** and **14**.	S10
**Table S4**.	Unit cell and refinement data for polymorphs and pseudopolymorphs of **19**.	S11
**Table S5**.	Unit cell and refinement data for [**20**][PF_6], [**16**][PF_6]·½[**17**], and [**18**][PF_6].	S12
**Figure S1**.	Thermal ellipsoid plot (50%) with atom labeling for compound **7**.	S13
**Figure S2**.	Thermal ellipsoid plot (50%) with atom labeling for compound **4b**.	S13
**Figure S3**.	Thermal ellipsoid plot (50%) with atom labeling for compound **4c**.	S14
**Figure S4**.	Thermal ellipsoid plot (50%) with atom labeling for compound [**4a**·H_2]Cl_2.	S14
**Figure S5**.	Thermal ellipsoid plot (50%) with atom labeling for disulfide compound **9**.	S15
**Figure S6**.	Thermal ellipsoid plot (50%) with atom labeling for compound **10**.	S15
**Figure S7**.	Thermal ellipsoid plot (50%) with atom labeling for compound **11**.	S16
**Figure S8**.	Thermal ellipsoid plot (50%) with atom labeling for compound **13** (JPD572).	S16
**Figure S9**.	Thermal ellipsoid plot (50%) for compound **13**, side view.	S17
**Figure S10**.	Thermal ellipsoid plot (50%) for interstitial C_5H_{12} in **13**·C_5H_{12}.	S17
**Figure S11**.	Thermal ellipsoid plot (50%) with atom labeling for compound **13** (JPD812).	S18
**Figure S12**.	Thermal ellipsoid plot (50%) with atom labeling for **14**.	S19
**Figure S13**.	Thermal ellipsoid plot (50%) for **14** with Me carbons omitted for clarity.	S20
**Figure S14**.	Core structure of **14** with all hydrogen, carbon, nitrogen toms omitted.	S21
**Figure S15**.	Core structure of **14** with view orthogonal to that in **Figure S14**.	S21
**Figure S16**.	Atom labeling for interstitial C_6H_6 molecule 1 in **14**·3.05(C_6H_6)·0.45($C_6H_4Me_2$)·0.5(H_2O).	S22
**Figure S17**.	Atom labeling for interstitial H_2O and interstitial molecule modeled as 0.45:0.55 *o*-xylene:benzene in **14**·3.05(C_6H_6)·0.45($C_6H_4Me_2$)·0.5(H_2O).	S22
**Figure S18**.	Atom labeling for interstitial C_6H_6 molecule 3 in **14**·3.05(C_6H_6)·0.45($C_6H_4Me_2$)·0.5(H_2O).	S23
**Figure S19**.	Atom labeling for interstitial half-molecule 4 of C_6H_6 in **14**·3.05(C_6H_6)·0.45($C_6H_4Me_2$)·0.5(H_2O).	S23
**Figure S20**.	Thermal ellipsoid plot (50%) with atom labeling for **19**, molecule 1 of 2, *P*-1 polymorph (JPD513).	S24
**Figure S21**.	Thermal ellipsoid plot (50%) with atom labeling for **19**, molecule 2 of 2, *P*-1 polymorph (JPD513).	S25
**Figure S22**.	Thermal ellipsoid plot (50%) with atom labeling for **19**, *C*2/*c* polymorph, (JPD718).	S26
**Figure S23**.	Thermal ellipsoid plot (50%) with atom labeling for **19** in **19**·2THF, *C*2/*c* pseudopolymorph (JPD722).	S27
**Figure S24**.	Atom labeling for interstitial THF in **19**·2THF (JPD722).	S27
**Figure S25**.	Thermal ellipsoid plot (50%) with atom labeling for **19**, molecule 1 of 2, in *P*-1 pseudopolymorph (JPD838).	S28
**Figure S26**.	Thermal ellipsoid plot (50%) with atom labeling for **19**, molecule 2 of 2, in *P*-1 pseudopolymorph (JPD838).	S29

Table of Contents, Continued

**Figure S27**.	Atom labeling for interstitial Et_2O in 2(**19**)·½Et_2O (JPD838)	S30
**Figure S28**.	Relative disposition of molecules in the asymmetric unit for 2(**19**)·½Et_2O (JPD838).	S30
**Figure S29**.	Thermal ellipsoid plot (50%) for [**16**]$^{1+}$, cation 1 of 2, with H atoms omitted for clarity. Only half the cation is unique.	S31
**Figure S30**.	Thermal ellipsoid plot (50%) for [**16**]$^{1+}$, cation 2 of 2, with H atoms omitted for clarity. Only half the cation is unique.	S32
**Figure S31**.	Thermal ellipsoid plot (50%) for hexacopper cage compound **17**, with H atoms omitted for clarity. Only half the cage is unique.	S33
**Figure S32**.	Atom labeling for counteranions and interstitial solvent in [**16**][PF_6]·½[**17**]·^{t}BuOMe·2MeCN (JPD776).	S33
**Figure S33**.	Thermal ellipsoid plot (50%) with atom labeling for [**20**]$^{2+}$.	S34
**Figure S34**.	Atom labeling for anions and solvent in [**20**][PF_6]$_2$·2MeCN	S34
**Figure S35**.	Thermal ellipsoid plot (50%) with atom labeling for [**18**]$^{1+}$.	S35
**Figure S36**.	Atom labeling for anions and solvent in [**18**][PF_6]·C_5H_{12}.	S35
**Figure S37**.	Elemental analysis results for **7**.	S36
**Figure S38**.	Elemental analysis results for **4b**.	S37
**Figure S39**.	Elemental analysis results for **19**.	S38
**Figure S40**.	Elemental analysis results for **14**.	S39
**Figure S41**.	^{1}H NMR spectrum (CD_2Cl_2) for **4a**.	S40
**Figure S42**.	^{13}C NMR spectrum (CD_2Cl_2) for **4a**.	S41
**Figure S43**.	^{1}H NMR spectrum (CD_2Cl_2) for **4b**.	S42
**Figure S44**.	^{13}C NMR spectrum (CD_2Cl_2) for **4b**.	S43
**Figure S45**.	^{1}H NMR spectrum (CD_2Cl_2) for **4c**.	S44
**Figure S46**.	^{1}H NMR spectrum (CD_3OD) for [**4a**·2H]Cl_2.	S45
**Figure S47**.	^{1}H NMR spectrum (C_6D_6) for **7**.	S46
**Figure S48**.	^{13}C NMR spectrum (C_6D_6) for **7**.	S47
**Figure S49**.	^{1}H NMR spectrum (CD_2Cl_2) for L-N_2(S^{Ar}H)$_2$, **5**.	S48
**Figure S50**.	^{13}C NMR spectrum (CD_2Cl_2) for L-N_2(S^{Ar}H)$_2$, **5**.	S49
**Figure S51**.	^{1}H NMR spectrum ($CDCl_3$) for **10**.	S50
**Figure S52**.	^{1}H NMR spectrum (CD_2Cl_2) for **11**.	S51
**Figure S53**.	^{1}H NMR spectrum (C_6D_6) of [(Cu(L-N_2(S^{Me_2})$_2$))$_2$$Cu_2$], **13**.	S52
**Figure S54**.	^{1}H NMR spectrum (C_6D_6) of [(Cu(L-N_2(S^{Me_2})$_2$))$_2$$Cu_2$], **13**, 4.0-0.0 ppm.	S53
**Figure S55**.	^{1}H NMR spectrum (CD_2Cl_2) for [**18**][PF_6].	S54
**Figure S56**.	^{1}H NMR spectrum (CD_2Cl_2) for **19**.	S55
**Figure S57**.	^{1}H NMR spectrum (CD_3CN) for [**19**][PF_6]/[**20**][PF_6]$_2$.	S56
**Figure S58**.	UV-vis spectrum (CH_2Cl_2) of **13**.	S57
**Figure S59**.	Mass spectrum (ESI^+) of **13**.	S58
**Figure S60**.	Mass spectrum (ESI^+) of [**18**]$^+$ and **19**.	S59
**Figure S61**.	Close up mass spectrum (ESI^+) of [**18**]$^+$ and **19**.	S60
**Figure S62**.	Second-derivative X-band EPR spectrum of **19** in CH_2Cl_2.	S61
**Figure S63**.	Mulliken spin density plot for **19**.	S62
**Figure S64**.	CV for **19** with scanning initiated in the cathodic direction.	S63
**Scheme S1**.	Possible mechanism for formation of cyclic **10** from disulfide **9**.	S64
**References**.		S65-S66

Procedures for Crystal Growth, Collection and Processing of Diffraction Data, and Solving and Refining of Structures

Diffraction-quality crystals of of N^1,N^2-bis(2-(^{i}propylthio)phenyl)-N^1,N^2-dimethylethane-1,2-diamine bis(hydrochloride), [**4a**·H_2]Cl_2, were obtained as colorless plates by the layered diffusion of hexanes into an EtOH solution. Thick, colorless plate crystals of N^1,N^2-bis(2-(^{t}butylthio)phenyl)-N^1,N^2-dimethylethane-1,2-diamine, **4b**, were grown by evaporation of a hexanes solution. Slow diffusion of *n*-pentane vapor into a THF solution of of N^1,N^2-bis(2-(benzylthio)phenyl)-N^1,N^2-dimethylethane-1,2-diamine, **4c**, produced colorless block crystals. Slow cooling of an EtOH solution of N^1,N^2-bis(2-fluorophenyl)-N^1,N^2-dimethylethane-1,2-diamine, **6**, was effective in growing colorless block-shaped crystals. The same method but with MeOH produced yellow, column-shaped crystals of **9**. Yellow, block-shaped crystals of 2,8,14-Trithiatetracyclo[13.3.1.1^{3,7}.1^{9,13}]heneicosa-1(19),3,5, 7(21),9,11,13(20),15,17-nonaene, 6,12,18-tris(*N*-methylamine), **10**, and yellow, column-shaped crystals of 2,8,14,15-Tetrathiatetracyclo[14.3.1.1^{3,7}.1^{9,13}]docosa-1(20),3,5,7(22),9,11,13(21),16,18-nonaene, 6,12,19-tri(*N*-methylamine)-, **11**, were grown as a mixture by evaporation of a solution in CH_2Cl_2/hexanes.

Most of the crystals of the copper complexes used in X-ray diffraction data collections were obtained by the vial-in-a-vial vapor diffusion technique. The following solvent pairs provide specific combinations successfully employed as solvent/diffusing vapor for crystal growth: [Cu-(L-$N_2S^{Me_2}{}_2$)Cu_2] (**13**) (colorless blocks), THF/hexanes; [Cu-(L-$N_2S^{Me_2}{}_2$)Cu_2]·C_5H_{12}, **13**·C_5H_{12} (colorless blocks), [(Cu(L-N_2(S^{Me_2})$_2$)$_3$($CuCl)_5$)]·3.05(C_6H_6)·0.45($C_6H_4Me_2$)·0.5(H_2O), **14**·3.05(C_6H_6)·0.45($C_6H_4Me_2$)·0.5(H_2O) (dark blue plates), 1,2-dichlorobenzene/*o*-xylene-C_6H_6; [[Cu_2(Cu-L-$N_2S^{Ar}{}_2$)$_3$(Cu(N≡CMe))]$_2$Cu][PF_6]·½[Cu_2(Cu-L-$N_2S^{Ar}{}_2$)$_3$(Cu(N≡CMe))]·^{t}BuOMe·2MeCN, [**16**][PF_6]·½**17**·^{t}BuOMe·2MeCN (small, thin colorless plates) MeCN/^{t}BuOMe; [(Cu_2(Cu-L-$N_2S^{Ar}{}_2$)$_3$)(Cu(N≡CMe))][PF_6]·C_5H_{12}, **18**[PF_6]·C_5H_{12} (blue-tinted plates) THF/*n*-pentane. [Cu_2(Cu($N_2S^{Ar}{}_2$))$_3$], **19**, monoclinic polymorph in *C*2/*c* (intense green-yellow thick plates), THF/*n*-pentane; [Cu_2(Cu($N_2S^{Ar}{}_2$))$_3$]·2THF, **19**·2THF, (dark green-yellow blocks), THF/*n*-pentane; [Cu_2(Cu($N_2S^{Ar}{}_2$))$_3$]·½Et_2O, **19**·½Et_2O, (brown plates), THF/Et_2O; [Cu_4((Cu($N_2S^{Ar}{}_2$))$_6$][PF_6]$_2$·2MeCN, [**20**][PF_6]·2MeCN, (black wedges), MeCN/Et_2O; A triclinic polymorph of [Cu_2(Cu($N_2S^{Ar}{}_2$))$_3$] in *P*-1, **19** (brown plates), was obtained, upon standing, from the pentane/Et_2O washings of the crude product resulting from reaction between [MoOCl(L-N_2SAr_2)], [CuCl(IMes)] and $Me_3SiSSiMe_3$ (IMes = 1,3-bis(2,4,6-trimethylphenyl)imidazol-2-ylidene).

All crystals were coated with paratone oil and mounted on the end of a nylon loop attached to the end of the goniometer. Data were collected at either 100 K, 150 K or 170 K under a dry N_2 stream supplied under the control of an Oxford Cryostream 800 attachment. The data collection instrument was either a Bruker Smart APEX II CCD or Bruker D8 Quest Photon 3 diffractometer equipped with a Mo fine-focus sealed tube providing radiation at λ = 0.71073 nm or a Bruker D8 Venture diffractometer operating with a Photon 100 CMOS detector and a Cu Incoatec I microfocus source generating X-rays at λ = 1.54178 nm.

The data sets collected with the Smart APEX diffractometer implemented one of the following programmed routines: (1) Three sets of 363 frames in *ω* (0.5º/scan) with *φ* held constant at 0, 120, and then 240º; (2) Three sets of 400 frames in *ω* (0.5º/scan), collected at *φ* = 0.00, 90.00 and 180.00° and two sets of 800 frames in *φ* (0.45°/scan) collected with *ω* constant at –30.00 and 210.00°. The data collection program and frame time used for these data sets were as follows: **4b** (JPD664), Routine 2, 20 sec; **4c** (JPD574), Routine 2, 15 sec; **10** (JPD623), Routine 2, 20 sec; **13**·Et_2O (JPD572), Routine 2, 20 sec; **13** (JPD812), Routine 2, 30 sec; **19** (JPD513), Routine 2, 120 sec; **19**·2THF (JPD722), Routine 2, 20 sec; **19**·Et_2O (JPD838), Routine 1, 60 sec; [**20**][PF_6]$_2$·2MeCN (JPD745), Routine 2, 60 sec; [**18**][PF_6]·*n*-C_5H_{12} (JPD778), Routine 1, 30 sec. The data for **14**·3.05(C_6H_6)·0.45($C_6H_4Me_2$)·0.5(H_2O) (JPD1189a) were obtained on the D8 Quest Photon 3 from 8 x 373 and 1 x 720 frame sets at 15 sec/frame. All remaining data sets were collected with the D8 Venture and were hemispheres of data comprised from the following assemblies of ω-scan frames and frame times: **9** (JPD616), 7 sets of 326 frames at 20 sec/frame; **11** (JPD626), 9 sets of 326 or 330 frames at 30 or 60 sec/frame; **6** (JPD640), 6 sets of 326 or 330 frames at 10 sec/frame; [**4a**·H_2]Cl_2 (JPD651), 6 sets of 326 or 330 frames at 20 sec/frame; **19** (JPD718), 14 sets of 326 or 330 frames at 10/sec/frame; [**16**][PF_6]·½**17**·^{t}BuOMe·2MeCN (JPD776), 14 sets of 326 or 330 frames at 60/sec/frame.

All data were collected under control of either the Bruker APEX2[1a-1d] or APEX3[1e-1f] software packages. Raw data were reduced to F^2 values using SAINT,[2] and a global refinement of unit cell parameters was performed using ~8000-9950 selected reflections from the full data set, except for **4b** (5224 reflections) and **7** (3412 reflections), which were comparatively smaller data sets. For [**4a**·H_2]Cl_2 (JPD651), analysis of 2940 reflections having I/σ(I) > 12 and chosen from the full data set with *CELL_NOW*[3] showed the crystal to belong to the monoclinic system and to be twinned by a 180° rotation about the *c** axis. The raw data were processed using the multi-component

version of *SAINT* under control of the two-component orientation file generated by *CELL_NOW* with an absorption correction was applied using the *TWINABS* routine.[4] In similar fashion, [**18**][PF_6]·C_5H_{12} (JPD778) was treated as a three-component twin. All other data sets were corrected for absorption on the basis of multiple measurements of symmetry equivalent reflections or by numerical methods with the use of *SADABS*,[5] as described by Krause *et al*.[6] All structure solutions were obtained by direct methods using *SHELXS*[7] or *SHELXT*,[8] while refinements were accomplished by full-matrix least-squares procedures using *SHELXL*.[9] Both the *SHELXS* and *SHELXL* programs are incorporated into the *SHELXTL*[10] and *APEX2*/*APEX3*[1] software suites.

In all the structures, hydrogen atoms were added in calculated positions and included as riding contributions with isotropic displacement parameters 1.2-1.5 times those of the carbon atoms to which they were attached. In a few instances, minor parts of the molecule/coordination complex were disordered over two positions (*e.g.,* the *tert*-butyl groups in N^1,N^2-bis(2-(^{t}butylthio)phenyl)-N^1,N^2-dimethylethane-1,2-diamine, **4b** (JPD664), and the terminal methyl group of the MeCN ligand in **17** (JPD776)), and were treated using the usual split-atom model with a site-occupancy distribution determined as a best-fit by the refinement software. Where present, the PF_6^{1-} counteranions were typically disordered, which demanded implementation of interatomic distance restraints in order to achieve stable refinement. Interstitial solvent molecules that were similarly afflicted with disorder were generally handled with what minimal interatomic distance restraints were necessary to accomplish good refinement behavior. In [**16**][PF_6]·½**17**·^{t}BuOMe·2MeCN small amounts of density remote from the main coordination complex and attributable to partially occupied/disordered solvent sites were removed with the *SQUEEZE* routine in *PLATON*.[11] Thermal ellipsoid images have been created with the use of XP, which also is part of the SHELXTL package. All structures were checked for overlooked symmetry and other errors by the checkCIF service provided by the International Union of Crystallography.[12]

Computational Procedures

All calculations were performed with the electronic structure program ORCA.[13] The single point DFT calculation used the PBE0[14] hybrid on the crystallographic coordinates of **19** (without solvent molecules). The scalar relativistically recontracted def2-TZVP were used for all atoms.[15] Core electrons were kept frozen and described by single Slater functions (core shells: C and N, 1s; S and Cu, 1s2s2p). Calculations with hybrid functionals used the RIJCOSX algorithm to speed the calculation of Hartree–Fock Exchange.[16] Calculations included the zeroth-order regular approximation (ZORA) for relativistic effects[17] as implemented by van Wüllen.[18] Auxiliary basis sets for all complexes used to expand the electron density in the calculations were chosen to match the orbital basis. The conductor like screening model (COSMO) was used for all calculations.[19] The self-consistent field (SCF) calculations were tightly converged (1×10^{-8} E_h in energy, 1×10^{-7} E_h in the density charge, and 1×10^{-7} in the maximum element of the DIIS[20] error vector). The geometry search for all complexes was carried out in redundant internal coordinates without imposing geometry constraints. Charge and spin density maps were visualized in the programme *Molekel*.[21]

Table S1. Crystal and refinement data for *N*[1],*N*[2]-bis(2-(^{t}butylthio)phenyl)-*N*[1],*N*[2]-dimethylethane-1,2-diamine, **4b**; *N*[1],*N*[2]-bis(2-(benzylthio)phenyl)-*N*[1],*N*[2]-dimethylethane-1,2-diamine, **4c**; *N*[1],*N*[2]-bis(2-(^{i}propylthio)phenyl)-*N*[1],*N*[2]-dimethylethane-1,2-diamine bis(hydrochloride), [**4a**·H_2]Cl_2; *N*[1],*N*[2]-bis(2-fluorophenyl)-*N*[1],*N*[2]-dimethylethane-1,2-diamine, **7**.

compound #	**4b**	**4c**	[**4a**·H_2]Cl_2	**7**
structure code	JPD664_0m_a	JPD574_0m_a	JPD651_0m_a	JPD640_0m_a
solvent	none	none	none	none
formula	$C_{24}H_{36}N_2S_2$	$C_{30}H_{32}N_2S_2$	$C_{22}H_{34}Cl_2N_2S_2$	$C_{16}H_{18}F_2N_2$
fw	416.67	484.69	461.53	276.32
temperature, K	150	150	150	150
wavelength, Å	0.71073	0.71073	1.54178	1.54178
2θ range, deg.	4.186 - 55.344	4.694 – 56.55	6.810 – 144.792	11.344 – 139.828
crystal system	triclinic	monoclinic	monoclinic	monoclinic
space group	$P\overline{1}$	$P2_1/n$	$P2_1/c$	$P2_1/n$
a, Å	7.0210(5)	9.7229(7)	13.0510(4)	5.4275(1)
b, Å	9.4375(6)	9.3312(7)	10.1657(3)	8.3334(2)
c, Å	10.0316(7)	14.2753(10)	9.1259(3)	15.7929(3)
α, deg.	88.089(1)	90	90	90
β, deg.	76.270(1)	100.6420(11)	95.978(1)	98.996(1)
γ, deg.	69.802(1)	90	90	90
volume, Å^3	605.14(7)	1272.87(16)	1204.17(6)	705.52(3)
Z	1	2	2	2
density, g/cm^3	1.143	1.265	1.273	1.301
μ, mm^{-1}	0.232	0.231	4.113	0.792
F(000)	226	516	492	292
crystal size	0.070 x 0.312 x 0.470	0.120 x 0.120 x 0.150	0.022 x 0.207 x 0.424	0.105 x 0.114 x 0.206
color, habit	colorless wedge	colorless block	colorless plate	
limiting indices, *h*	$-9 \le h \le 9$	$-12 \le h \le 12$	$-11 \le h \le 11$	$-6 \le h \le 6$
limiting indices, *k*	$-12 \le k \le 12$	$-12 \le k \le 12$	$-11 \le k \le 11$	$-9 \le k \le 10$
limiting indices, *l*	$-13 \le l \le 13$	$-18 \le l \le 19$	$-12 \le l \le 14$	$-19 \le l \le 18$
reflections collected	10741	22040	2308	5211
independent data	2804	3148	2308	1322
restraints	0	41	0	0
parameters refined	144	155	130	92
GooF^a	1.116	1.045	1.066	1.103
R1,^{b,c} wR2^{d,c}	0.0395, 0.1102	0.0390, 0.0976	0.0349, 0.0881	0.0368, 0.0883
R1,^{b,e} wR2^{d,e}	0.0463, 0.1140	0.0500, 0.1056	0.0393, 0.0909	0.0446, 0.0930
largest diff. peak, e·Å^{-3}	0.424	0.380	0.328	0.184
largest diff. hole, e·Å^{-3}	–0.187	–0.281	–0.259	–0.174

. ^{a}GooF = $\{\Sigma[w(F_o^2 - F_c^2)^2]/(n-p)\}^{½}$, where *n*= number of reflections and *p* is the total number of parameters refined; ^{b}R1 = $\Sigma||F_o| - |F_c||/\Sigma|F_o|$; ^{c}R indices for data cut off at I > 2σ(I); ^{d}wR2 = $\{[\Sigma w(F_o^2 - F_c^2)^2/\Sigma w(F_o^2)^2\}^{1/2}$; $w = 1/[\sigma^2(F_o^2) + (xP)^2 + yP]$, where $P = (F_o^2 + 2F_c^2)/3$; ^{e}R indices for all data.

Table S2. Crystal and refinement data for **9**, **10**, and **11**.

compound #	**9**	**10**	**11**	
structure code	JPD616_0m_a	JPD623_a	JPD626_0m_a	
solvent	none	none	CH_2Cl_2	
formula	$C_{14}H_{16}N_2S_2$	$C_{21}H_{21}N_3S_3$	$C_2H_2N_2S_4 \cdot CH_2Cl_2$	
fw	276.41	411.59	CH_2Cl_2	
temperature, K	150	150	150	
wavelength, Å	1.54178	0.71073	1.54178	
2θ range, deg.	7.562 - 136.358	3.512 – 57.432	6.268 – 144.924	
crystal system	monoclinic	monoclinic	triclinic	
space group	$P2_1/c$	$P2_1/c$	$P\bar{1}$	
a, Å	12.4663(3)	11.5996(9)	8.2111(2)	
b, Å	7.9419(2)	10.5464(8)	10.6229(2)	
c, Å	14.5714(3)	15.7796(12)	15.0778(3)	
α, deg.	90	90	109.789(1)	
β, deg.	110.3330(9)	90.044(1)	91.824(1)	
γ, deg.	90	90	101.827(1)	
volume, $Å^3$	605.14(7)	1930.4(3)	1203.90(4)	
Z	4	4	2	
density, g/cm^3	1.357	1.416	1.458	
μ, mm^{-1}	3.417	0.396	5.793	
F(000)	584	864	548	
crystal size	0.112 x 0.139 x 0.280	0.092 x 0.132 x 0.194	0.028 x 0.050 x 0.167	
color, habit	yellow column	yellow block	yellow column	
limiting indices, *h*	$-15 \le h \le 14$	$-15 \le h \le 15$	$-10 \le h \le 10$	
limiting indices, *k*	$-9 \le k \le 9$	$-14 \le k \le 14$	$-13 \le k \le 12$	
limiting indices, *l*	$-16 \le l \le 17$	$-21 \le l \le 21$	$-18 \le l \le 18$	
reflections collected	10484	34564	13015	
independent data	2438	4973	4648	
restraints	0	0	0	
parameters refined	165	247	283	
GooF^a	1.044	1.047	1.043	
R1,^{b,c} wR2^{d,c}	0.0291, 0.0776	0.0520, 0.1368	0.0493, 0.1184	
R1,^{b,e} wR2^{d,e}	0.0326, 0.0801	0.0659, 0.1495	0.0664, 0.1292	
largest diff. peak, e·$Å^{-3}$	0.209	1.119	0.544	
largest diff. hole, e·$Å^{-3}$	–0.325	–0.345	–0.376	

. ^{a}GooF = $\{\Sigma[w(F_o^2 - F_c^2)^2]/(n - p)\}^{½}$, where n= number of reflections and p is the total number of parameters refined; ^{b}R1 = $\Sigma||F_o| - |F_c||/\Sigma|F_o|$; ^{c}R indices for data cut off at I > 2σ(I); ^{d}wR2 = $\{[\Sigma w(F_o^2 - F_c^2)^2/\Sigma w(F_o^2)^2\}^{1/2}$; $w = 1/[\sigma^2(F_o^2) + (xP)^2 + yP]$, where $P = (F_o^2 + 2F_c^2)/3$; ^{e}R indices for all data.

Table S3. Crystal and Refinement Data for $[[Cu(L\text{-}N_2S^{Me_2}{}_2)]_2Cu_2]$ and $[[Cu(L\text{-}N_2S^{Me_2}{}_2)]_2Cu_2]\cdot½C_5H_{12}$, and $[(Cu(L\text{-}N_2S^{Me_2}{}_2))_3(CuCl)_5]\cdot 3.05C_6H_6\cdot 0.45C_6H_4Me_2\cdot ½H_2O$.

compound	$[[Cu(L\text{-}N_2S^{Me_2}{}_2)]_2Cu_2]$	$[[Cu(L\text{-}N_2S^{Me_2}{}_2)]_2Cu_2]$	$[(Cu(L\text{-}N_2S^{Me_2}{}_2))_3(CuCl)_5]$
compound #	**13**	**13**	**14**
structure code	JPD812_0m_a	JPD572_0m_a	JPD1189a_0m_a
solvent	none	$½C_5H_{12}$	$3.05C_6H_6\cdot 0.45C_6H_4Me_2\cdot ½H_2O$
formula	$C_{24}H_{52}Cu_4N_4S_4$	$C_{26.50}H_{58}Cu_4N_4S_4$	$C_{58}H_{100.75}Cl_5Cu_8N_6O_{0.50}S_6$
fw	779.09	815.17	1768.12
temperature, K	150	150	170
wavelength, Å	0.71073	0.71073	0.71073
2θ range, deg.	2.970 - 60.380	3.864 - 57.542	4.496 – 58.442
crystal system	monoclinic	monoclinic	tetragonal
space group	$P2_1/c$	$P2_1/c$	$P4_2/n$
a, Å	13.8940(11)	13.1795(15)	27.8548(9)
b, Å	12.0059(10)	13.1678(15)	27.8548(9)
c, Å	20.8983(17)	21.683(3)	19.1577(9)
α, deg.	90	90	90
β, deg.	99.227(1)	103.5610(16)	90
γ, deg.	90	90	90
volume, $Å^3$	3440.9(5)	3658.0(7)	14864.3(12)
Z	4	4	8
density, g/cm^3	1.504	1.480	1.580
μ, mm^{-1}	2.701	2.544	2.631
F(000)	1616	1700	7262
crystal size	0.163 x 0.270 x 0.310	0.100 x 0.140 x 0.140	0.157 x 0.272 x 0.307
color, habit	colorless block	colorless block	dark blue plate
limiting indices, *h*	$-19 \leq h \leq 19$	$-17 \leq h \leq 17$	$-38 \leq h \leq 38$
limiting indices, *k*	$-16 \leq k \leq 16$	$-17 \leq k \leq 17$	$-38 \leq h \leq 38$
limiting indices, *l*	$-29 \leq l \leq 29$	$-28 \leq l \leq 29$	$-26 \leq l \leq 26$
reflections collected	66379	63078	672248
independent data	9775	9431	20103
restraints	0	179	9
parameters refined	337	382	687
GooF[a]	1.056	1.045	1.147
R1,[b,c] wR2[d,c]	0.0235, 0.0632	0.0359, 0.0837	0.0503, 0.1255
R1,[b,e] wR2[d,e]	0.0296, 0.0651	0.0517, 0.0928	0.0755, 0.1501
largest diff. peak, $e\cdot Å^{-3}$	0.721	0.909	2.353
largest diff. hole, $e\cdot Å^{-3}$	–0.243	–0.513	–0.767

[a]GooF = $\{\Sigma[w(F_o^2 - F_c^2)^2]/(n-p)\}^{½}$, where *n*= number of reflections and *p* is the total number of parameters refined; [b]R1 = $\Sigma||F_o| - |F_c||/\Sigma|F_o|$; [c]R indices for data cut off at I > 2σ(I); [d]wR2 = $\{[\Sigma w(F_o^2 - F_c^2)^2/\Sigma w(F_o^2)^2\}^{1/2}$; $w = 1/[\sigma^2(F_o^2) + (xP)^2 + yP]$, where $P = (F_o^2 + 2F_c^2)/3$; [e]R indices for all data.

Table S4. Crystal and Refinement Data for [[Cu(L-$N_2S^{Ar}{}_2$)]$_3Cu_2$] (triclinic polymorph), [[Cu(L-$N_2S^{Ar}{}_2$)]$_3Cu_2$] (monoclinic polymorph), [[Cu(L-$N_2S^{Ar}{}_2$)]$_3Cu_2$]·2(THF), and [[Cu(L-$N_2S^{Ar}{}_2$)]$_3Cu_2$]·½Et_2O.

compound	[[Cu(L-$N_2S^{Ar}{}_2$)]$_3Cu_2$]	[[Cu(L-$N_2S^{Ar}{}_2$)]$_3Cu_2$]	[[Cu(L-$N_2S^{Ar}{}_2$)]$_3Cu_2$]	[[Cu(L-$N_2S^{Ar}{}_2$)]$_3Cu_2$]
compound #	**19**	**19**	**19**	**19**
structure code	JPD513_0m_a	JPD718_0m	JPD722_0m_a	JPD838_0m_a
solvent	none	none	2(THF)	½Et_2O
formula	$C_{48}H_{54}Cu_5N_6S_6$	$C_{48}H_{54}Cu_5N_6S_6$	$C_{56}H_{70}Cu_5N_6O_2S_6$	$C_{50}H_{59}Cu_5N_6O_{0.5}S_6$
fw	1225.03	1225.03	1369.24	1262.18
temperature, K	150	150	100	100
wavelength, Å	0.71073	1.54178	0.71073	0.71073
2θ range, deg.	3.820 - 45.220	7.768 – 145.026	3.292 - 58.478	2.582 to 57.876
crystal system	triclinic	monoclinic	monoclinic	triclinic
space group	$P\bar{1}$	*C*2/*c*	*C*2/*c*	$P\bar{1}$
a, Å	11.840(2)	14.1957(3)	40.455(3)	11.977(2)
b, Å	20.650(4)	19.5106(4)	11.6468(9)	20.149(3)
c, Å	20.801(4)	18.3275(3)	27.274(2)	22.241(4)
α, deg.	85.228(2)	90	90	81.920(3)
β, deg.	89.839(2)	99.193(1)	114.855(1)	80.215(3)
γ, deg.	84.436(2)	90	90	86.994(2)
volume, $Å^3$	5044.0(16)	5010.91(17)	11660.4(16)	5234.6(15)
Z	4	4	8	4
density, g/cm^3	1.613	1.624	1.560	1.601
μ, mm^{-1}	2.363	4.994	2.056	2.280
F(000)	2500	2500	5640	2584
crystal size	0.03 x 0.12 x 0.24	0.160 x 0.150 x 0.090	0.140 x 0.210 x 0.240	0.020 x 0.120 x 0.415
color, habit	brown plate	green-yellow plate	green-yellow block	brown plate
limiting indices, *h*	$-12 \le h \le 12$	$-17 \le h \le 17$	$-55 \le \mathrm{h} \le 55$	$-15 \le h \le 15$
limiting indices, *k*	$-22 \le k \le 22$	$-24 \le k \le 24$	$-15 \le \mathrm{k} \le 15$	$-26 \le k \le 27$
limiting indices, *l*	$-22 \le l \le 22$	$-22 \le l \le 22$	$-37 \le \mathrm{l} \le 37$	$-30 \le l \le 30$
reflections collected	53618	43912	111732	50672
independent data	13343	4920	15694	25877
restraints	590	0	0	30
parameters refined	1183	297	682	1230
GooF[a]	1.035	1.058	1.026	1.001
R1,[b,c] wR2[d,c]	0.0483, 0.1041	0.0280, 0.0704	0.0336, 0.0834	0.0822, 0.1822
R1,[b,e] wR2[d,e]	0.0761, 0.1134	0.0313, 0.0722	0.0477, 0.0873	0.1590, 0.2215
largest diff. peak, e·$Å^{-3}$	0.705	0.440	1.242	2.319
largest diff. hole, e·$Å^{-3}$	–0.571	–0.453	–0.426	–1.760

[a]GooF = $\{\Sigma[w(F_o^2 - F_c^2)^2]/(n-p)\}^{1/2}$, where *n*= number of reflections and *p* is the total number of parameters refined; [b]R1 = $\Sigma||F_o| - |F_c||/\Sigma|F_o|$; [c]R indices for data cut off at I > 2σ(I); [d]wR2 = $\{[\Sigma w(F_o^2 - F_c^2)^2/\Sigma w(F_o^2)^2\}^{1/2}$; $w = 1/[\sigma^2(F_o^2) + (xP)^2 + yP]$, where $P = (F_o^2 + 2F_c^2)/3$; [e]R indices for all data.

Table S5. Unit Cell and Refinement Data for $[[[Cu(L\text{-}N_2S^{Ar}{}_2)]_3Cu_2]_2][PF_6]_2\cdot 2MeCN$ ([**20**]$[PF_6]_2\cdot 2MeCN$), $[[Cu_2(Cu(L\text{-}N_2S^{Ar}{}_2))_3(Cu(N{\equiv}CMe))]_2Cu][PF_6]\cdot ½[Cu_2(Cu(L\text{-}N_2S^{Ar}{}_2))_3(Cu(N{\equiv}CMe))]\cdot {}^tBuOMe\cdot 2MeCN$, ([**16**]$[PF_6]\cdot ½$**17**$\cdot {}^tBuOMe\cdot 2MeCN$) and $[[Cu(L\text{-}N_2S^{Ar}{}_2)]_3Cu_2(Cu(N{\equiv}CMe))][PF_6]\cdot n\text{-}C_5H_{12}$ ([**18**]$[PF_6]\cdot n\text{-}C_5H_{12}$).

compound	$[[[Cu(L\text{-}N_2S^{Ar}{}_2)]_3Cu_2]_2][PF_6]_2\cdot 2MeCN$	$[[Cu_2(Cu(L\text{-}N_2S^{Ar}{}_2))_3(Cu(N{\equiv}CMe))]_2Cu][PF_6]\cdot$ $½[Cu_2(Cu(L\text{-}N_2S^{Ar}{}_2))_3(Cu(N{\equiv}CMe))]$	$[[Cu(L\text{-}N_2S^{Ar}{}_2)]_3Cu_2(Cu(N{\equiv}CMe))][PF_6]$
compound #	[**20**]$^{2+}$	[**16**]$[PF_6]\cdot ½$[**17**]	[**18**]$[PF_6]$
structure code	JPD745_0m_a	JPD776_0m_a_sq	JPD778
solvent	2MeCN	${}^tBuOMe\cdot 2MeCN$	C_5H_{12}
formula	$C_{100}H_{102}Cu_{10}F_{12}N_{14}P_2S_{12}$	$C_{134}H_{160.50}Cu_{16}F_6N_{19.50}OPS_{15}$	$C_{55}H_{69}Cu_6F_6N_7P_1S_6$
fw	2810.01	3702.82	1546.84
temperature, K	150	100	100
wavelength, Å	0.71073	1.54178	0.71073
2θ range, deg.	4.012 - 46.236	4.400 – 93.114	2.354 – 56.948
crystal system	tetragonal	monoclinic	triclinic
space group	$I4_1/a$	$C2/c$	$P\bar{1}$
a, Å	27.910(3)	39.4732(12)	10.2198(18)
b, Å	27.910(3)	28.2023(9)	17.198(3)
c, Å	17.459(2)	32.1982(10)	17.552(3)
α, deg.	90	90	82.128(3)
β, deg.	90	109.113(1)	83.047(2)
γ, deg.	90	90	81.000(3)
volume, $Å^3$	13600(4)	33868.2(18)	3002.5(9)
Z	4	8	2
density, g/cm^3	1.378	1.452	1.711
μ, mm^{-1}	1.798	4.364	2.385
F(000)	5728	15056	1574
crystal size	0.117 x 0. 395 x 0.409	0.059 x 0.130 x 0.160	0.090 x 0.110 x 0.240
color, habit	black wedge	colorless plate	blue plate
limiting ind., *h*	$-30 \le h \le 30$	$-37 \le h \le 37$	$-13 \le h \le 13$
limiting ind., *k*	$-30 \le k \le 30$	$-26 \le k \le 26$	$-22 \le k \le 22$
limiting ind., *l*	$-19 \le l \le 19$	$-30 \le l \le 30$	$0 \le l \le 23$
refl. collected	79508	183126	14336
indep. data	4792	14806	14336
restraints	73	12	0
parameters ref'd	411	1742	707
GooF^a	1.117	1.048	0.966
R1,^{b,c} wR2^{d,c}	0.1116, 0.3072	0.0343, 0.0936	0.0841, 0.2054
R1,^{b,e} wR2^{d,e}	0.1226, 0.3173	0.0363, 0.0952	0.1297, 0.2312
diff. peak, $e\cdot Å^{-3}$	2.294	1.004	3.217,
diff. hole, $e\cdot Å^{-3}$	–0.635	–0.609	–1.431

^{a}GooF = $\{\Sigma[w(F_o^2 - F_c^2)^2]/(n - p)\}^{½}$, where n= number of reflections and p is the total number of parameters refined; ^{b}R1 = $\Sigma||F_o| - |F_c||/\Sigma|F_o|$; ^{c}R indices for data cut off at I > 2σ(I); ^{d}wR2 = $\{[\Sigma w(F_o^2 - F_c^2)^2/\Sigma w(F_o^2)^2\}^{1/2}$; $w = 1/[\sigma^2(F_o^2) + (xP)^2 + yP]$, where $P = (F_o^2 + 2F_c^2)/3$; ^{e}R indices for all data.

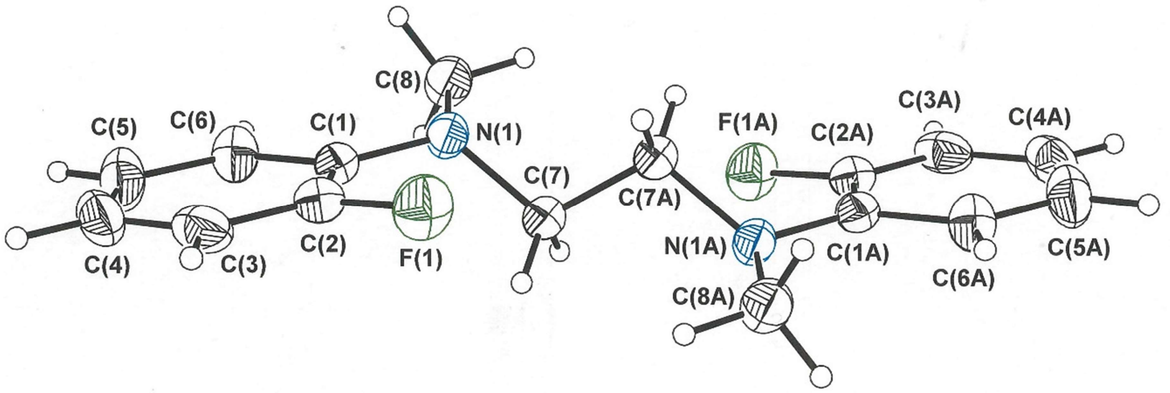

Figure S1. Thermal ellipsoid plot at the 50% probability level for N^1,N^2-bis(2-fluorophenyl)-N^1,N^2-dimethylethane-1,2-diamine, **7**.

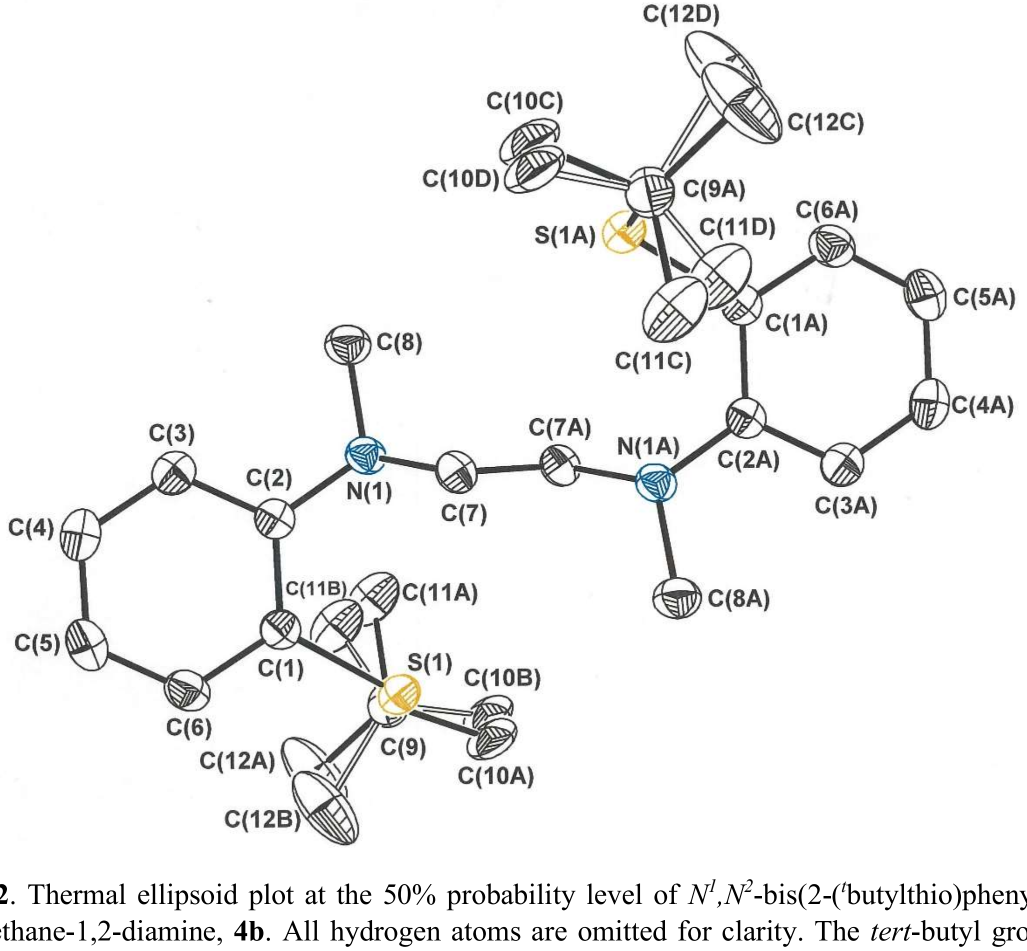

Figure S2. Thermal ellipsoid plot at the 50% probability level of N^1,N^2-bis(2-(^{t}butylthio)phenyl)-N^1,N^2-dimethylethane-1,2-diamine, **4b**. All hydrogen atoms are omitted for clarity. The *tert*-butyl groups have been treated as a best-fit distribution between two rotational orientations.

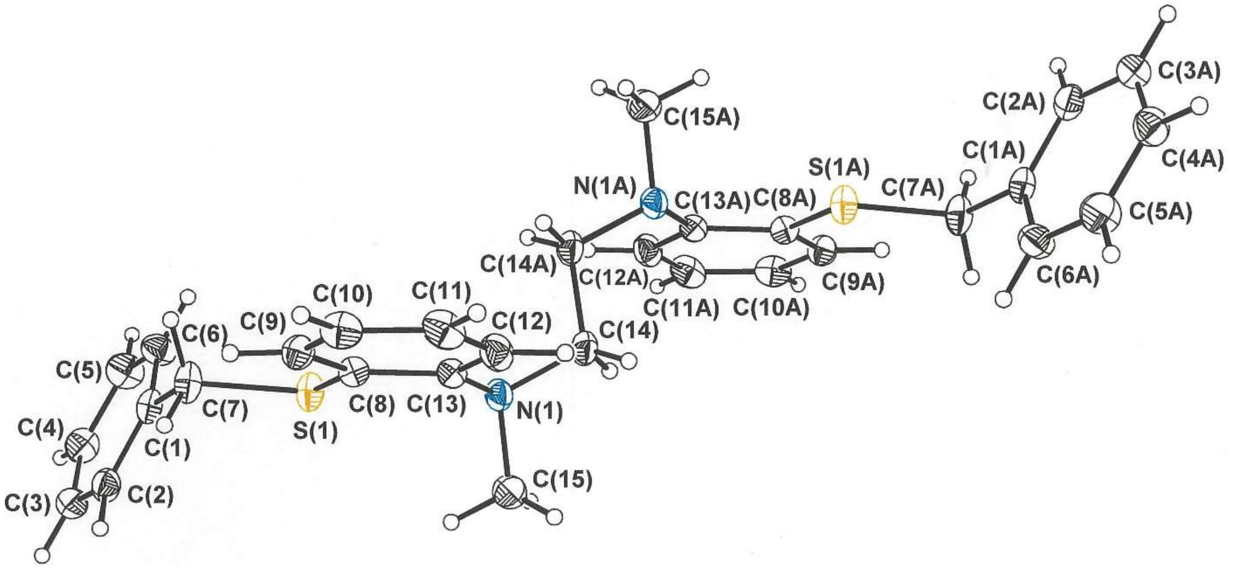

Figure S3. Thermal ellipsoid plot at the 50% probability level of N^1,N^2-bis(2-(benzylthio)phenyl)-N^1,N^2-dimethylethane-1,2-diamine, **4c**.

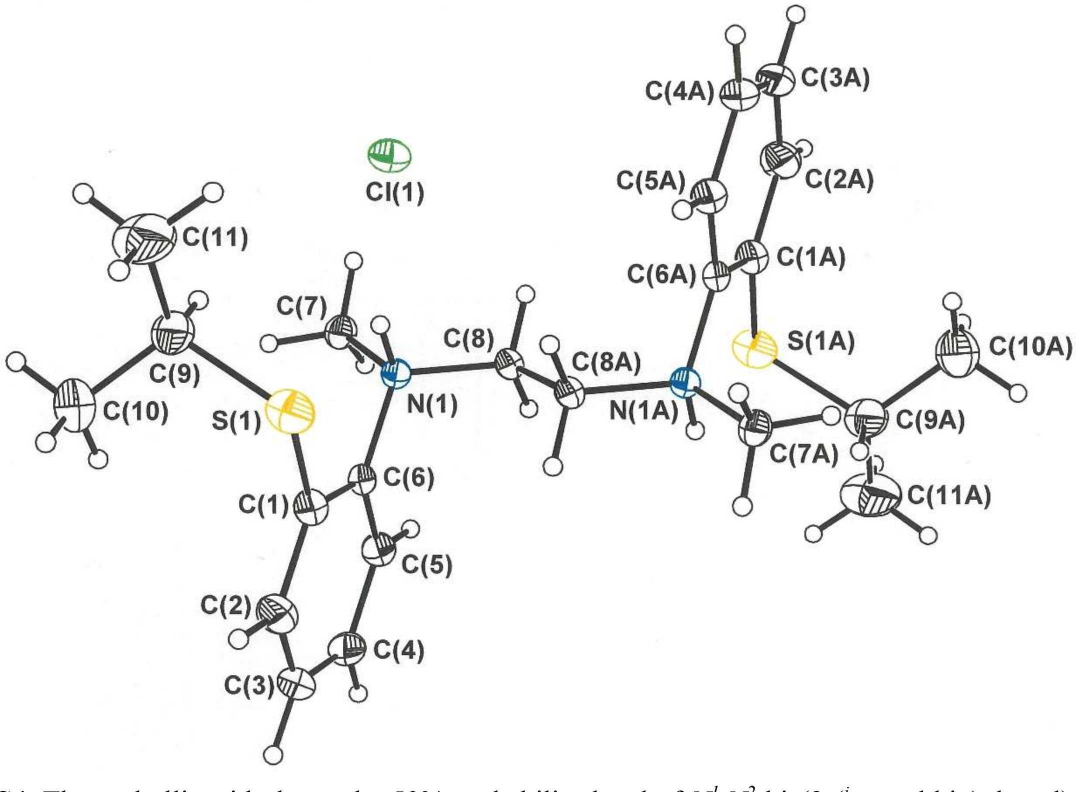

Figure S4. Thermal ellipsoid plot at the 50% probability level of N^1,N^2-bis(2-(^{i}propylthio)phenyl)-N^1,N^2-dimethylethane-1,2-diamine bis(hydrochloride), [**4a**·H_2]Cl_2.

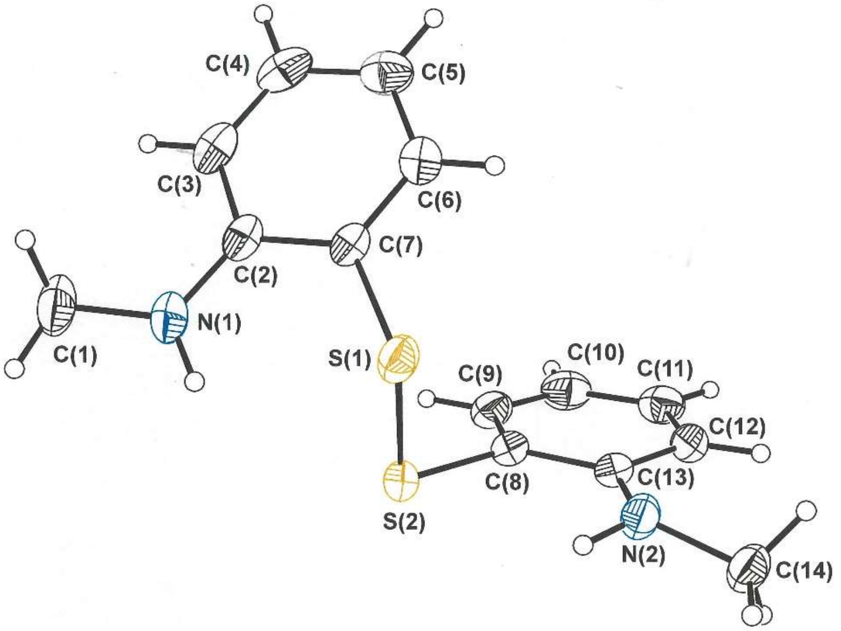

Figure S5. Thermal ellipsoid plot at the 50% probability level of bis[2-(methylamino)phenyl] disulfide, **9**.

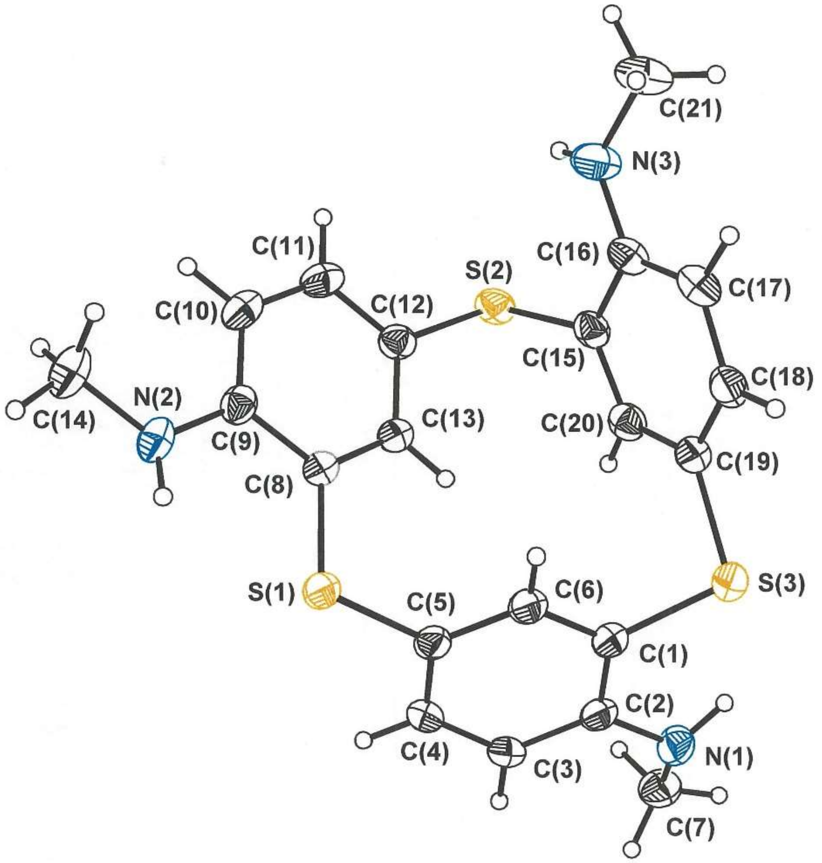

Figure S6. Thermal ellipsoid plot at the 50% probability level of 2,8,14-trithiatetracyclo[13.3.1.1^{3,7}.1^{9,13}]heneicosa-1(19),3,5,7(21),9,11,13(20),15,17-nonaene, 6,12,18-tris(*N*-methylamine), **10**.

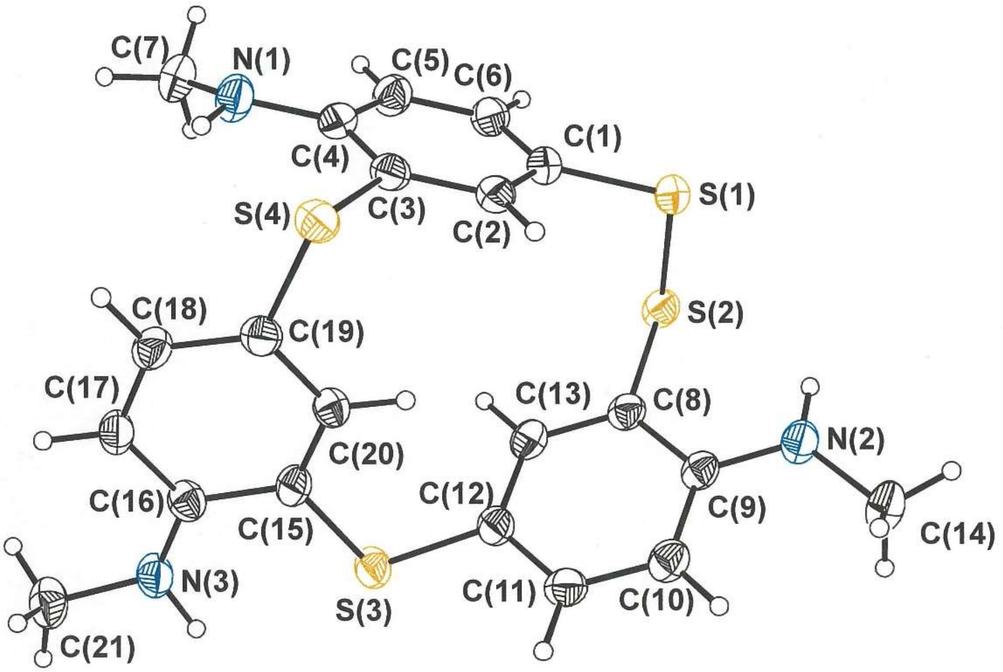

Figure S7. Thermal ellipsoid plot at the 50% probability level of 2,8,14-trithiatetracyclo[13.3.1.$1^{3,7}$.$1^{9,13}$]heneicosa-1(19),3,5,7(21),9,11,13(20),15,17-nonaene, 6,12,18-tris(*N*-methylamine), **11**.

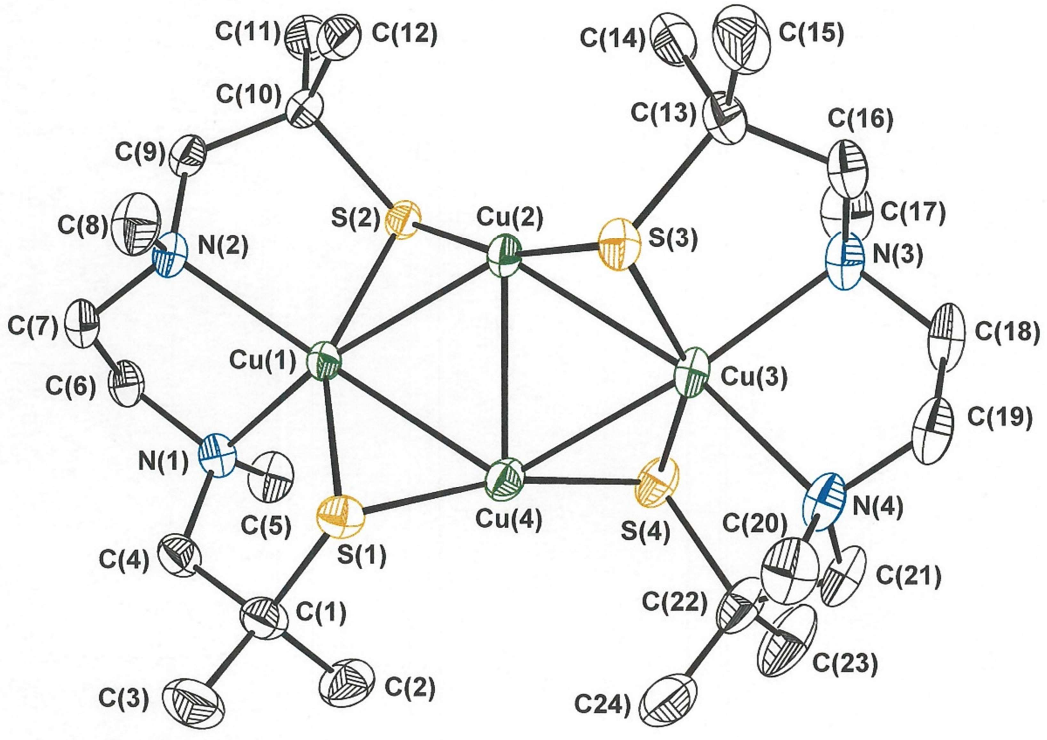

Figure S8. Thermal ellipsoid plot at the 50% probability level of [Cu_2(Cu(L-N_2(S^{Me_2})$_2$))$_2$], **13**, in [Cu_2(Cu(L-N_2(S^{Me_2})$_2$))$_2$]·½C_5H_{12} (data set JPD572). All hydrogen atoms are omitted for clarity.

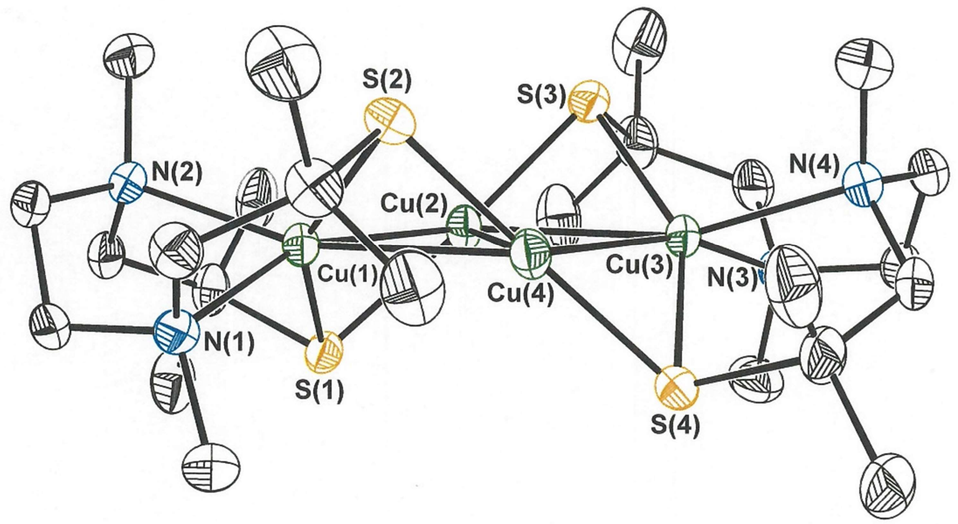

Figure S9. Thermal ellipsoid plot (side view) at the 50% probability level of $[Cu_2(Cu(L\text{-}N_2(S^{Me_2})_2))_2]$, **13**, in $[Cu_2(Cu(L\text{-}N_2(S^{Me_2})_2))_2]\cdot½C_5H_{12}$ (data set JPD572). All hydrogen atoms are omitted for clarity.

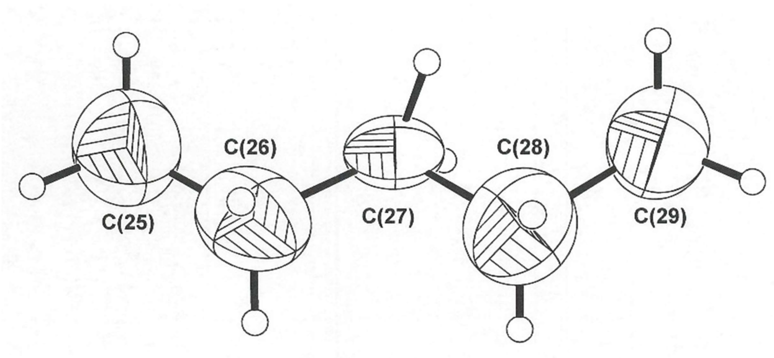

Figure S10. Thermal ellipsoid plot at the 50% probability level of interstitial C_5H_{12} in $[Cu_2(Cu(L\text{-}N_2(S^{Me_2})_2))_2]\cdot½C_5H_{12}$ (Data set JPD572).

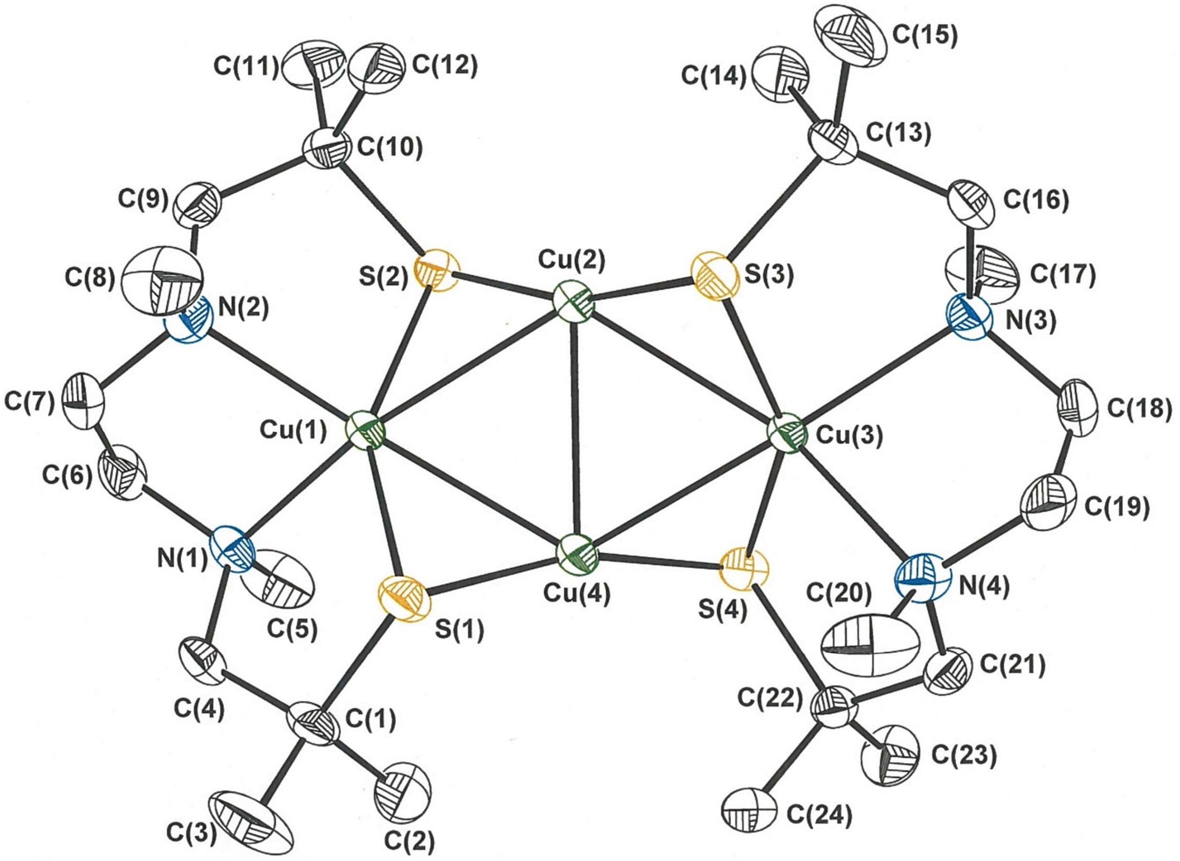

Figure S11. Thermal ellipsoid plot at the 50% probability level of $[Cu_2(Cu(L\text{-}N_2(S^{Me_2})_2))_2]$, **13**, in $[Cu_2(Cu(L\text{-}N_2(S^{Me_2})_2))_2]$ (Data set JPD812). All hydrogen atoms are omitted for clarity.

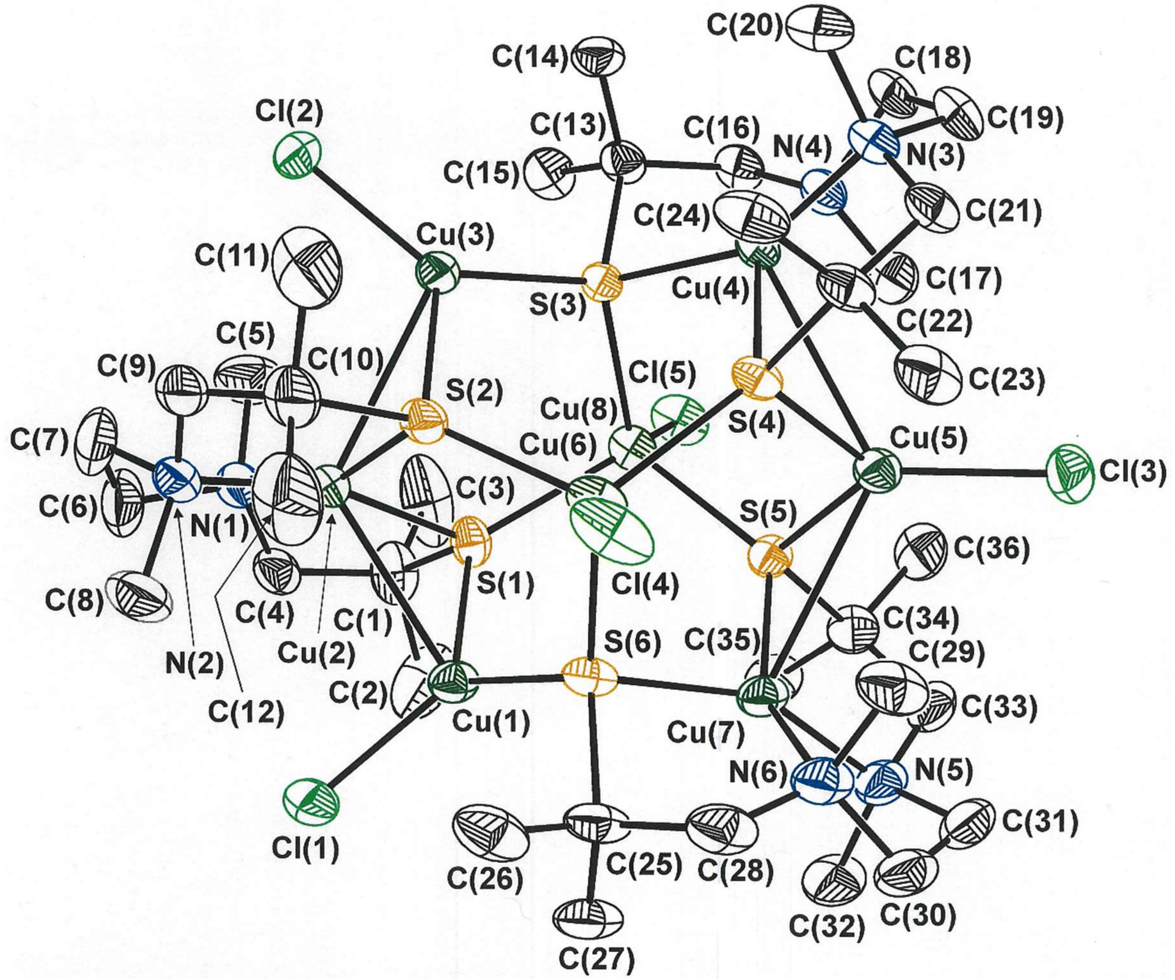

Figure S12. Thermal ellipsoid plot at the 50% probability level of [(Cu(L-$N_2S^{Me_2}{}_2$)$_3$(CuCl)$_5$], **14**, in [(Cu(L-$N_2S^{Me_2}{}_2$)$_3$(CuCl)$_5$]·3.05(C_6H_6)·0.45($C_6H_4Me_2$)·0.5(H_2O) (data set JPD1189a). All hydrogen atoms are omitted for clarity. The view is down the pseudo C_3 axis defined by Cu(6)···Cl(8).

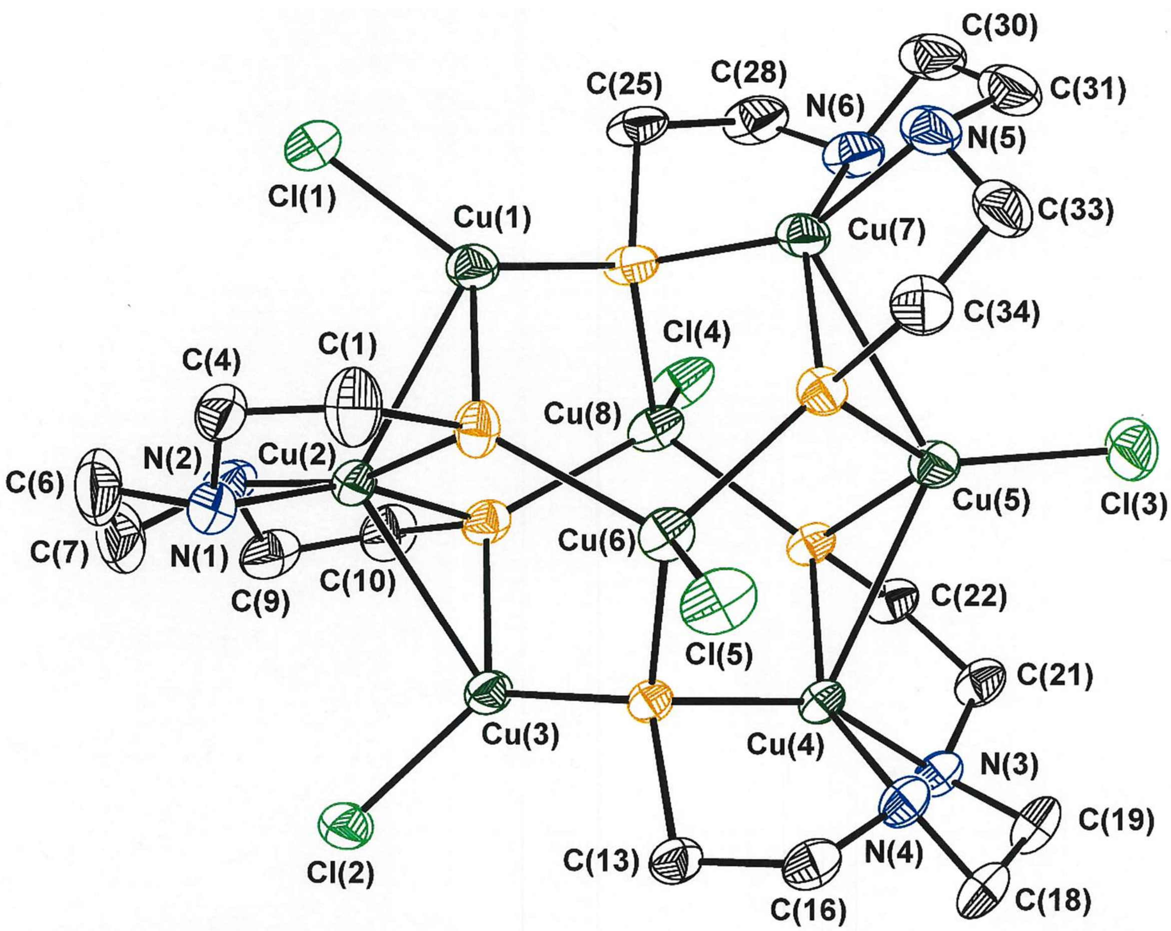

Figure S13. Thermal ellipsoid plot at the 50% probability level of $[(Cu(L\text{-}N_2S^{Me_2}{}_2)_3(CuCl)_5]$, **14**, in $[(Cu(L\text{-}N_2S^{Me_2}{}_2)_3(CuCl)_5]\cdot 3.05(C_6H_6)\cdot 0.45(C_6H_4Me_2)\cdot 0.5(H_2O)$ (data set JPD1189a). All hydrogen atoms and all Me groups are omitted for clarity. The view is down the pseudo C_3 axis defined by Cu(6)···Cl(8).

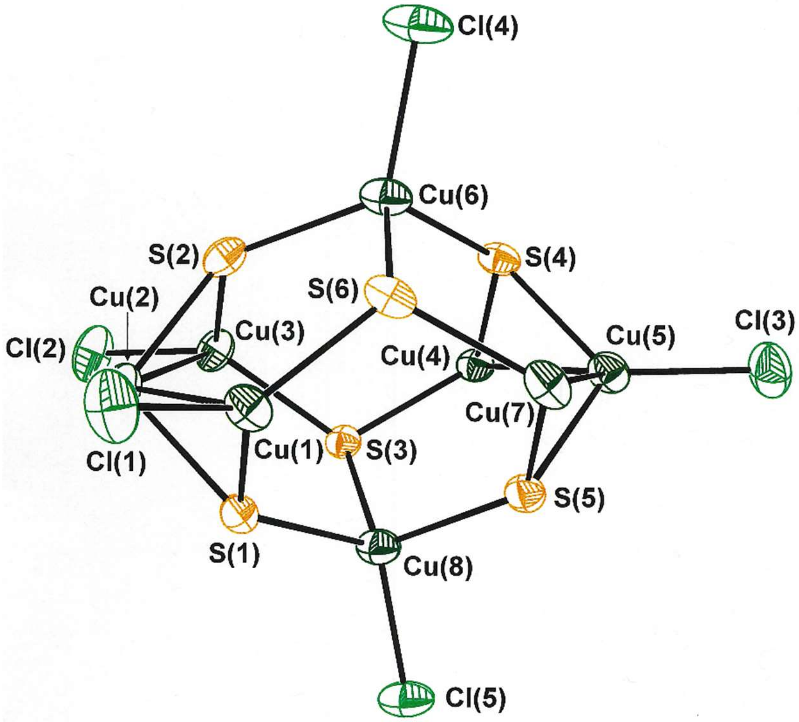

Figure S14. Core structure of $[(Cu(L\text{-}N_2S^{Me_2}{}_2))_3(CuCl)_5]$, **14**, in $[(Cu(L\text{-}N_2S^{Me_2}{}_2))_3(CuCl)_5]\cdot 3.05(C_6H_6)\cdot 0.45(C_6H_4Me_2)\cdot 0.5(H_2O)$ (data set JPD1189a). All hydrogen, carbon, and nitrogen atoms are omitted. The view is such that the pseudo C_3 axis defined by Cu(6)···Cl(8) is in the vertical direction.

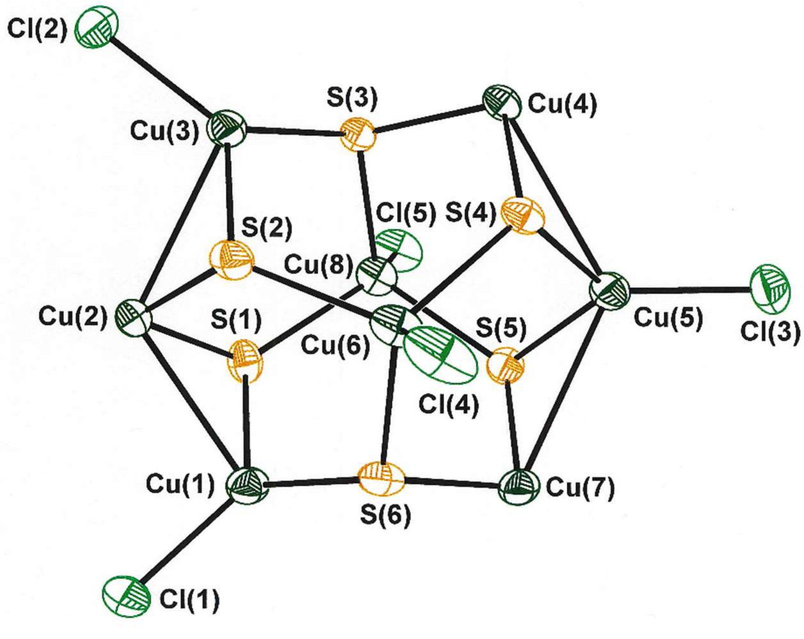

Figure S15. Core structure of $[(Cu(L\text{-}N_2S^{Me_2}{}_2))_3(CuCl)_5]$, **14**, in $[(Cu(L\text{-}N_2S^{Me_2}{}_2))_3(CuCl)_5]\cdot 3.05(C_6H_6)\cdot 0.45(C_6H_4Me_2)\cdot 0.5(H_2O)$ (data set JPD1189a). All hydrogen, carbon, and nitrogen atoms are omitted. The view is such that the pseudo C_3 axis defined by Cu(6)···Cl(8) is orthogonal to the plane of the paper.

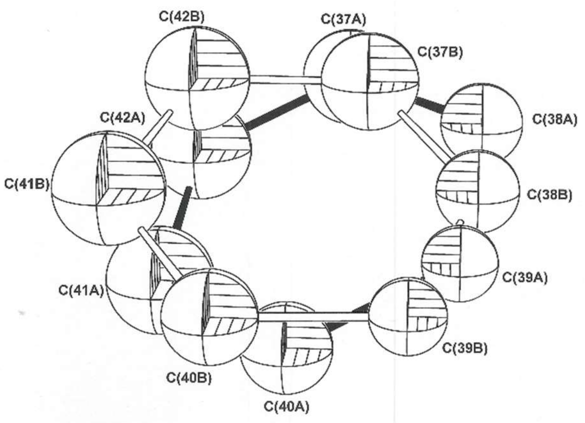

Figure S16. Atom labeling for disordered benzene molecule 1 in [(Cu(L-$N_2S^{Me_2}{}_2$)$_3$(CuCl)$_5$]·3.05(C_6H_6)·0.45($C_6H_4Me_2$)·0.5(H_2O) (data set JPD1189a). The ellipsoids are drawn at the 30% level, and all hydrogen atoms are omitted for clarity.

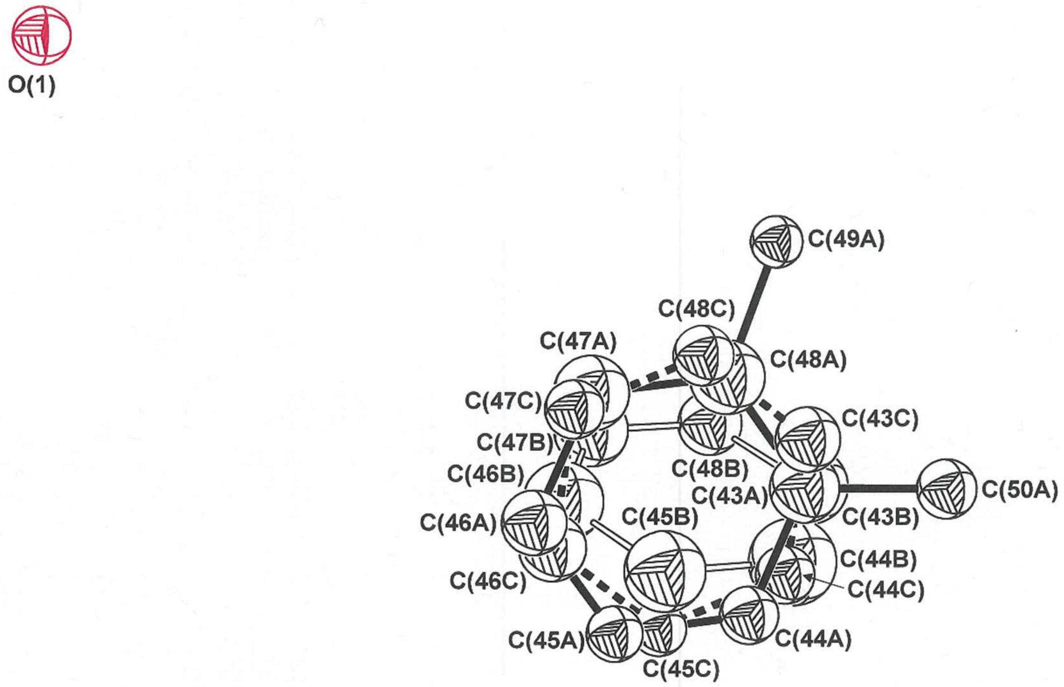

Figure S17. Atom labeling for interstitial H_2O and for disordered interstitial solvent molecule modeled as 0.45:0.55 *o*-xylene:benzene in [(Cu(L-$N_2S^{Me_2}{}_2$)$_3$(CuCl)$_5$]·3.05(C_6H_6)·0.45($C_6H_4Me_2$)·0.5(H_2O) (data set JPD1189a). The ellipsoids are drawn at the 30% level, and all hydrogen atoms are omitted for clarity.

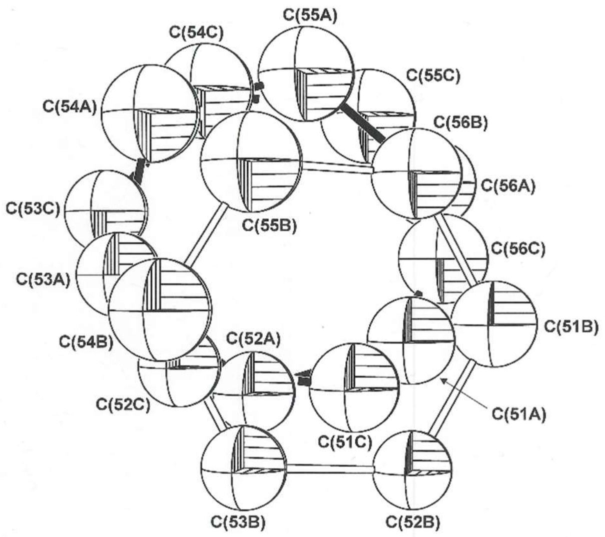

Figure S18. Atom labeling for disordered benzene molecule 3 in $[(Cu(L\text{-}N_2S^{Me_2}{}_2)_3(CuCl)_5]\cdot 3.05(C_6H_6)\cdot 0.45(C_6H_4Me_2)\cdot 0.5(H_2O)$ (data set JPD1189a). The ellipsoids are drawn at the 30% level, and all hydrogen atoms are omitted for clarity.

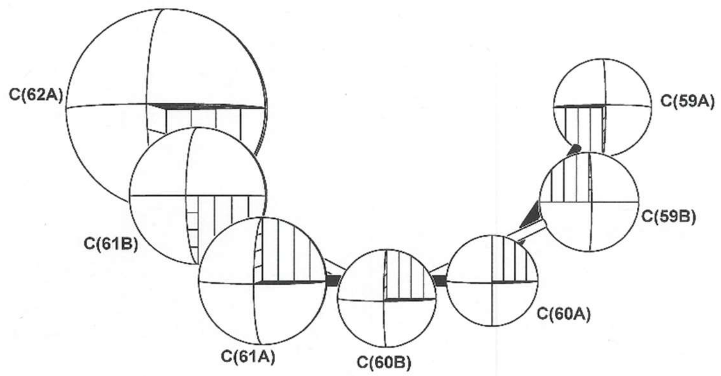

Figure S19. Atom labeling for disordered half-benzene molecule 4 in $[(Cu(L\text{-}N_2S^{Me_2}{}_2)_3(CuCl)_5]\cdot 3.05(C_6H_6)\cdot 0.45(C_6H_4Me_2)\cdot 0.5(H_2O)$ (data set JPD1189a). The ellipsoids are drawn at the 30% level, and all hydrogen atoms are omitted for clarity.

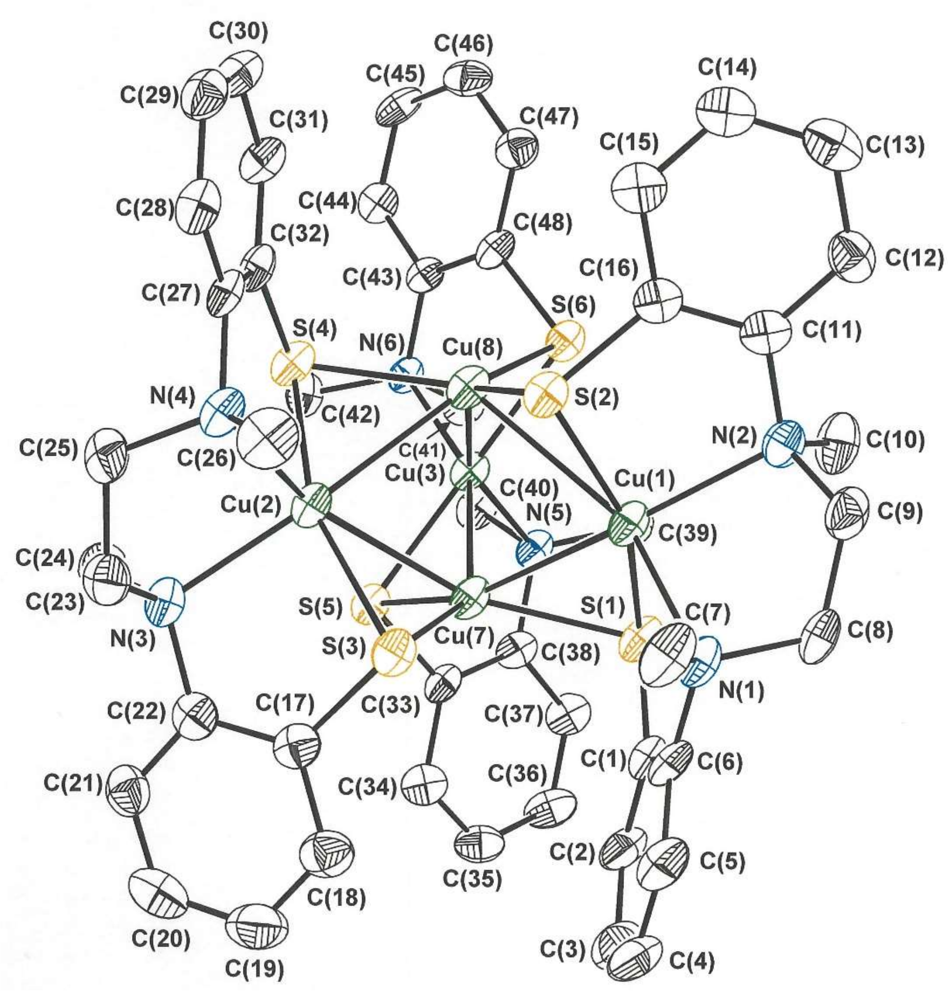

Figure S20. Thermal ellipsoid plot at the 50% probability level of $[Cu_2(Cu(N_2S^{Ar}{}_2))_3]$, **19**, molecule 1 of 2, in its *P*-1 polymorph (data set JPD513). All hydrogen atoms are omitted for clarity.

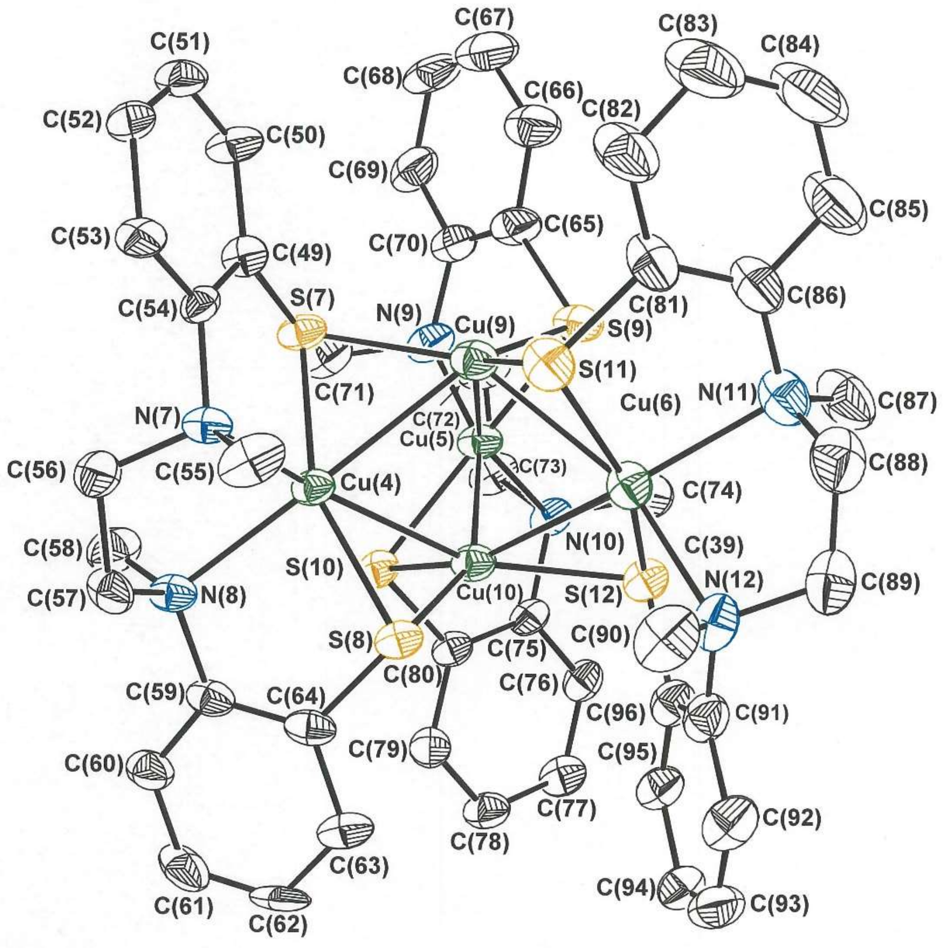

Figure S21. Thermal ellipsoid plot at the 50% probability level of $[Cu_2(Cu(N_2S^{Ar}{}_2))_3]$, **19**, molecule 2 of 2, in its *P*-1 polymorph (data set JPD513). All hydrogen atoms are omitted for clarity.

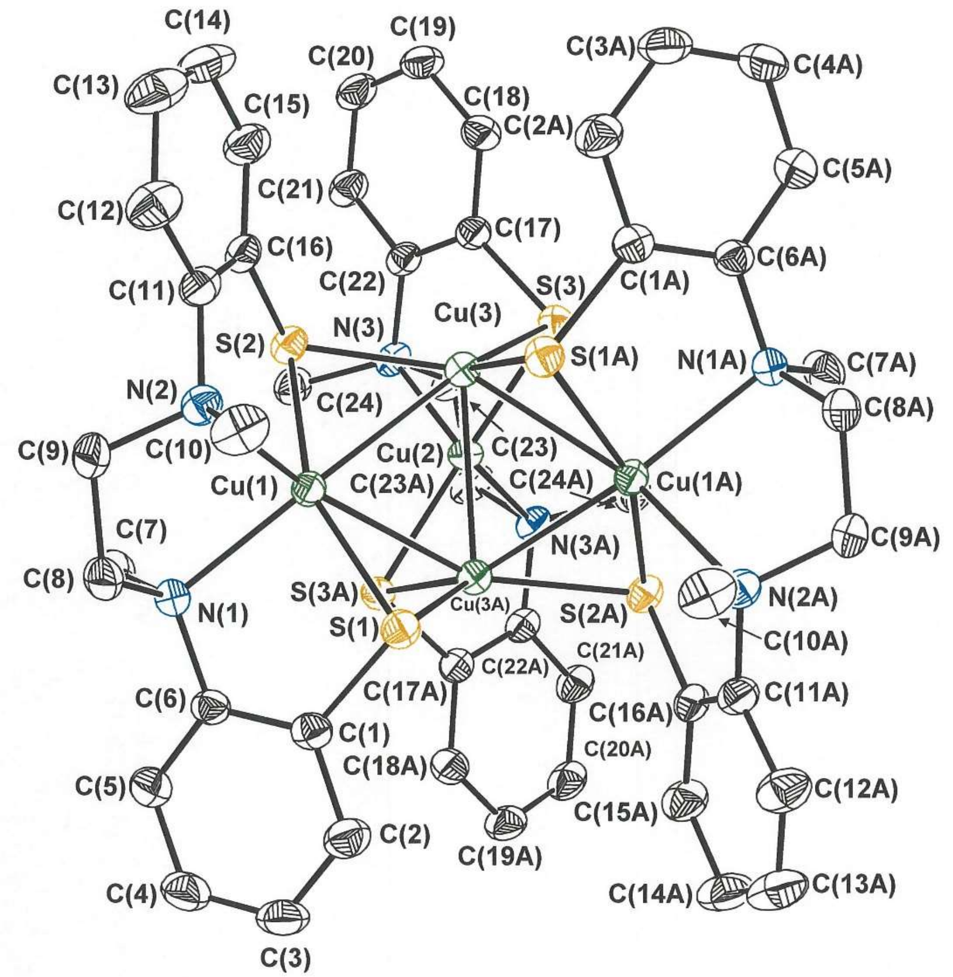

Figure S22. Thermal ellipsoid plot at the 50% probability level of [$Cu_2(Cu(N_2S^{Ar}{}_2))_3$], **19**, in its monoclinic *C*2/*c* polymorph (data set JPD718). All hydrogen atoms are omitted for clarity.

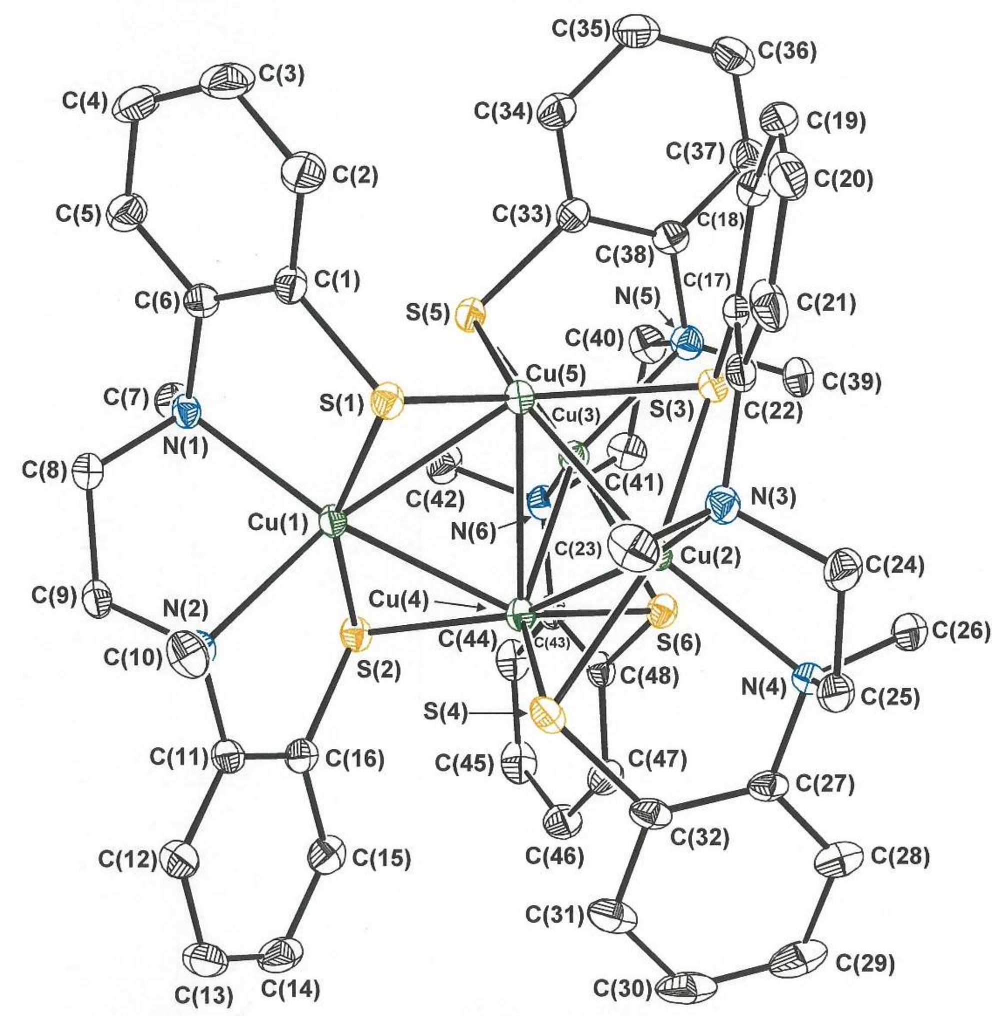

Figure S23. Thermal ellipsoid plot at the 50% probability level of $[Cu_2(Cu(N_2S^{Ar}{}_2))_3]$, **19**, in its crystal form (monoclinic $C2/c$) with 2 interstitial THF molecules (data set JPD722). All hydrogen atoms are omitted for clarity.

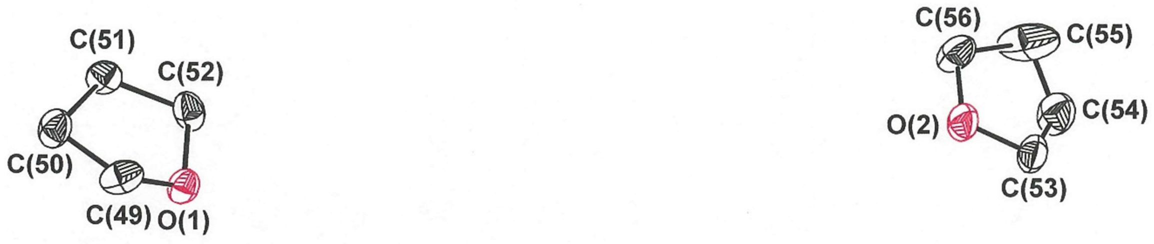

Figure S24. Thermal ellipsoid plot at the 50% probability level for the interstitial THF molecules in $[Cu_2(Cu(N_2S^{Ar}{}_2))_3]\cdot 2THF$ (data set JPD722). All hydrogen atoms are omitted for clarity.

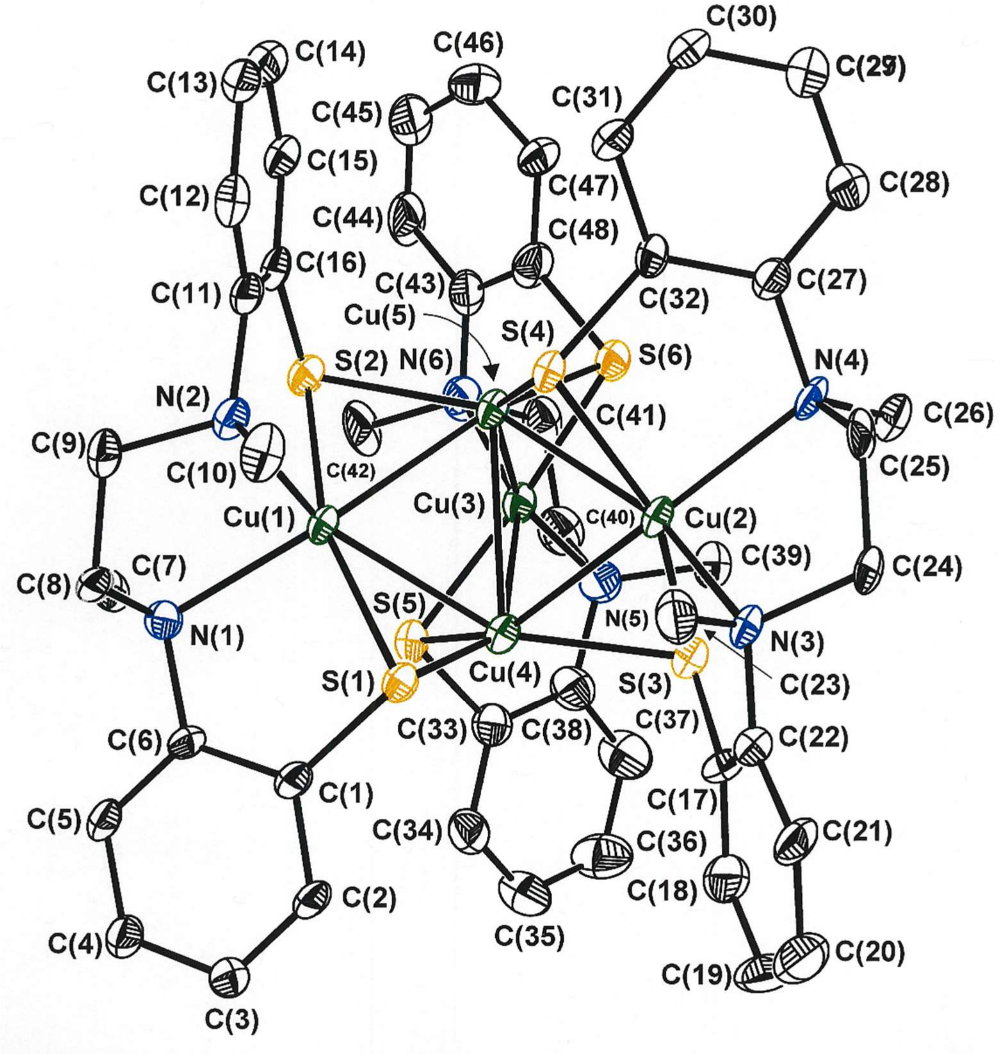

Figure S25. Thermal ellipsoid plot at the 50% probability level of [$Cu_2(Cu(N_2S^{Ar}{}_2))_3$], **19**, molecule 1 of 2, in its *P*-1 pseudopolymorph with ½Et_2O (Data set JPD838). All hydrogen atoms are omitted for clarity.

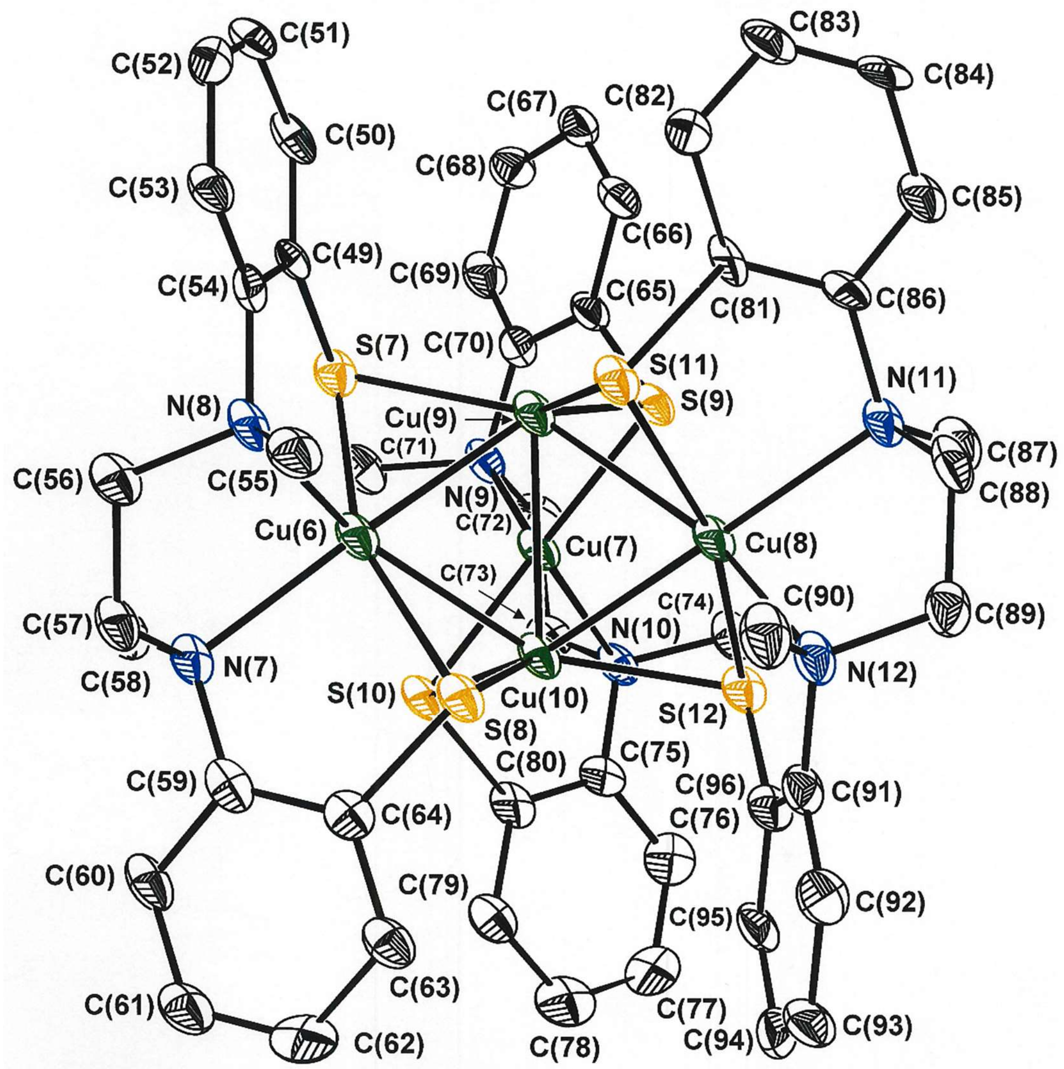

Figure S26. Thermal ellipsoid plot at the 50% probability level of $[Cu_2(Cu(N_2S^{Ar}{}_2))_3]$, **19**, molecule 2 of 2, in its *P*-1 pseudopolymorph with ½Et_2O (data set JPD838). All hydrogen atoms are omitted for clarity.

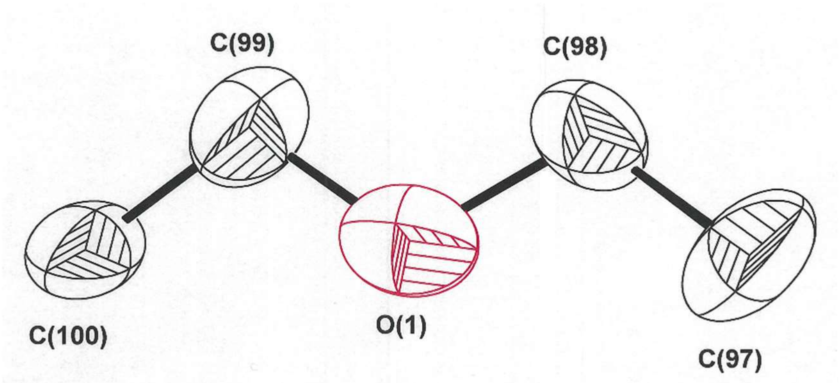

Figure S27. Atom labeling for interstitial Et_2O in $2[Cu_2(Cu(N_2S^{Ar}{}_2))_3]\cdot Et_2O$. The thermal ellipsoid plot is drawn at the 50% probability level, and all hydrogen atoms are omitted for clarity (data set JPD838).

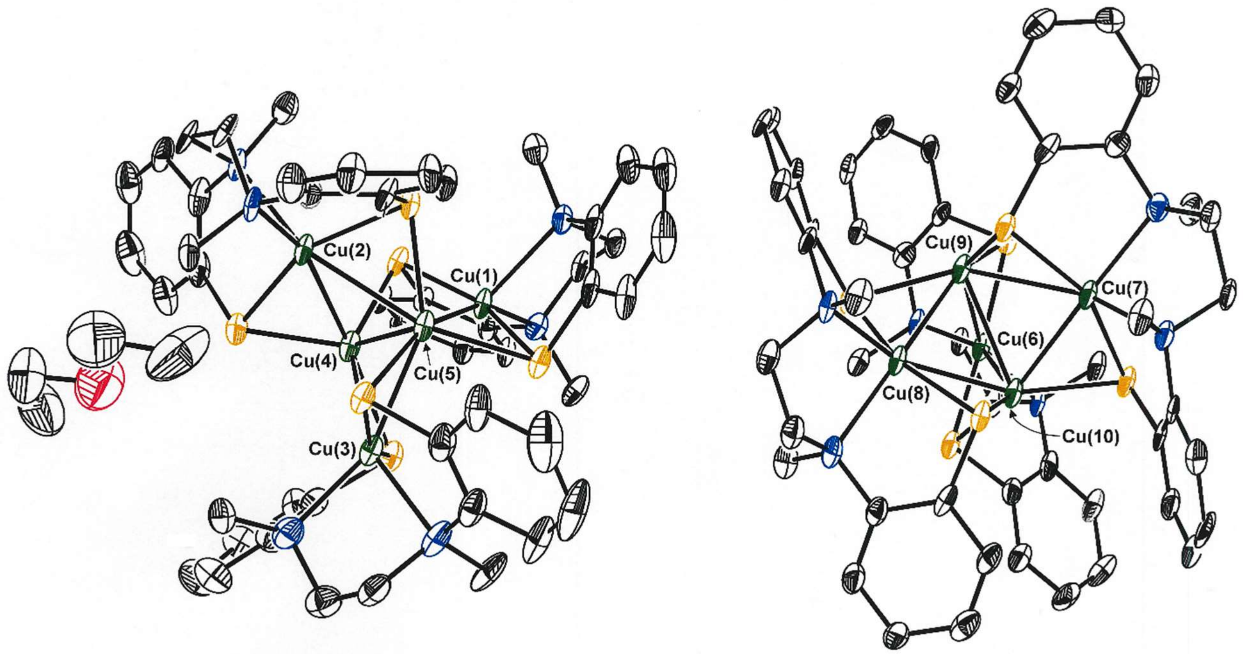

Figure S28. Relative disposition of the two molecules of $[Cu_2(Cu(N_2S^{Ar}{}_2))_3]$ in $2[Cu_2(Cu(N_2S^{Ar}{}_2))_3]\cdot Et_2O$. The thermal ellipsoid plot is drawn at the 50% probability level, and all hydrogen atoms are omitted for clarity (data set JPD838).

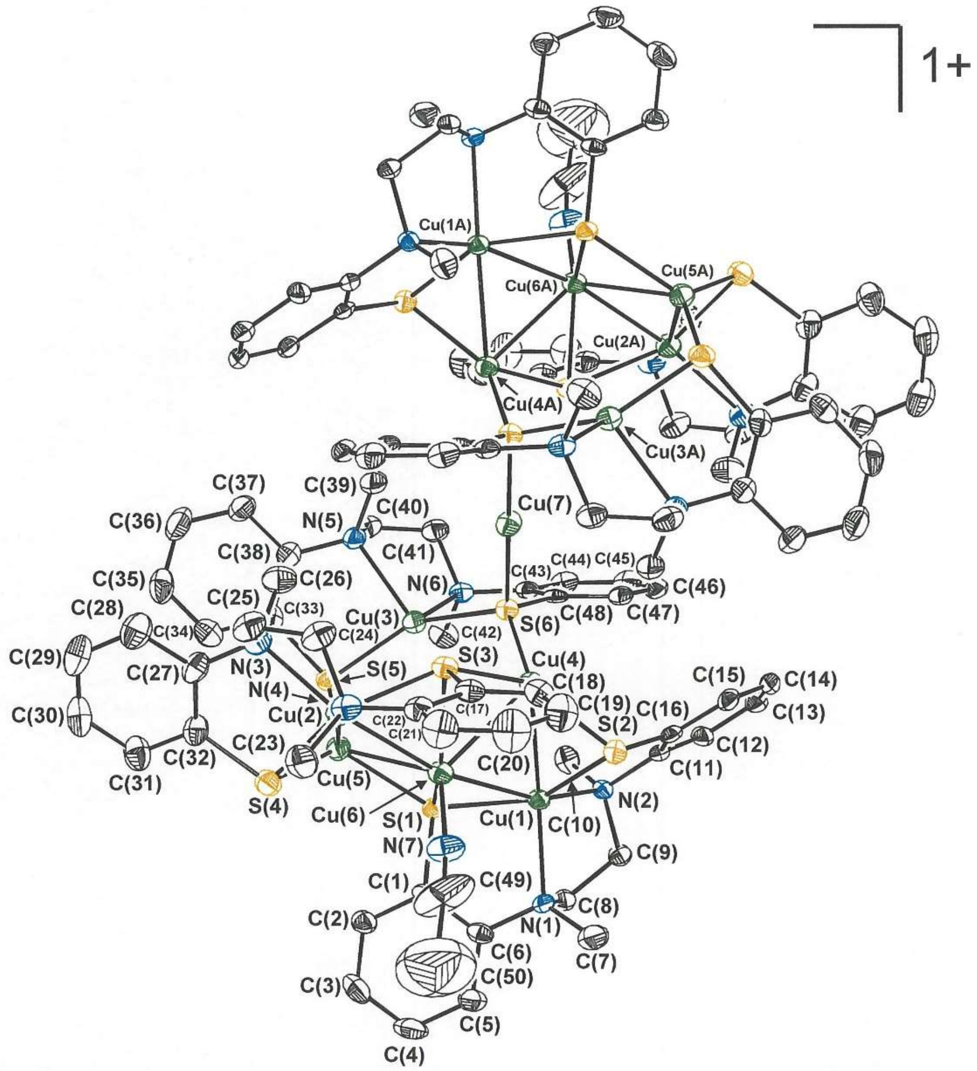

Figure S29. Thermal ellipsoid plot at the 50% probability level for half cation [[Cu_2(Cu-(L-$N_2S_2Me_2^{Ph}$))$_3$(Cu(N≡CMe))]$_2$Cu], one of two (data set JPD776). All hydrogen atoms are omitted for clarity. Cuprous ion Cu(7) coincides with an inversion center in monoclinic *C*2/*c*.

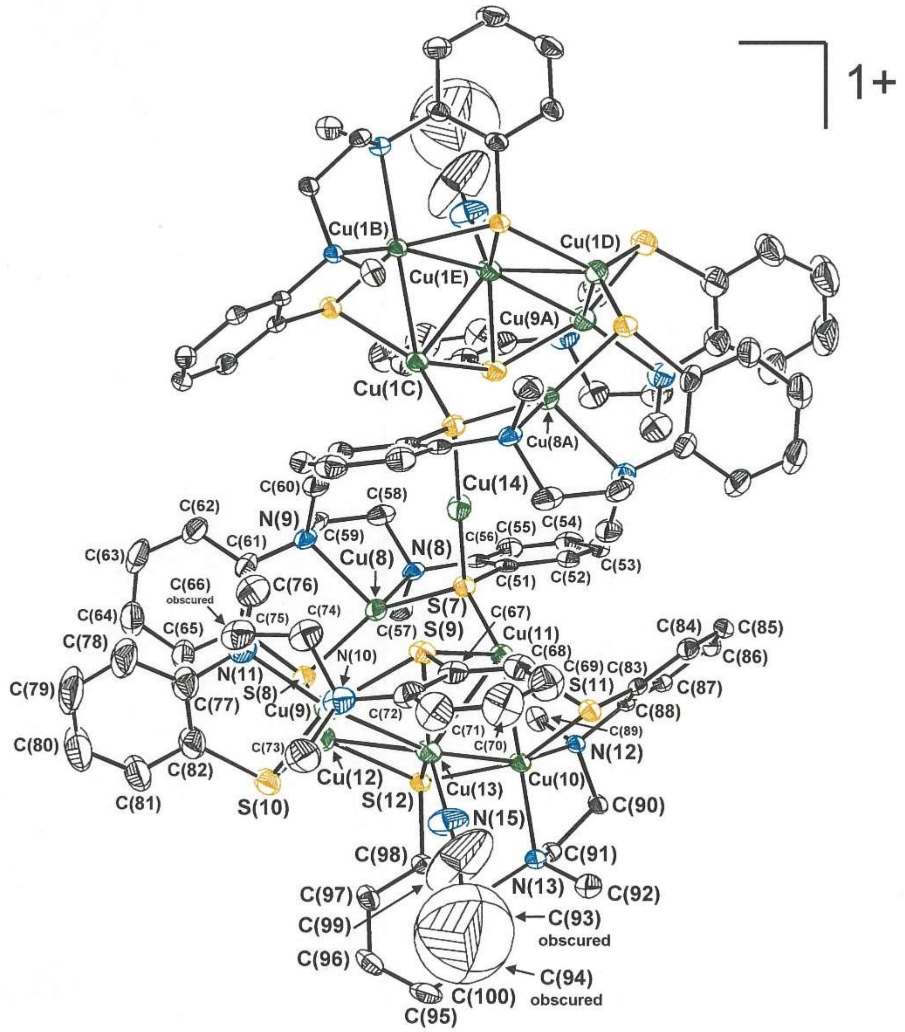

Figure S30. Thermal ellipsoid plot at the 50% probability level for half cation $[[Cu_2(Cu\text{-}(L\text{-}N_2S_2Me_2{}^{Ph}))_3(Cu(N{\equiv}CMe))]_2Cu]$, two of two (data set JPD776). All hydrogen atoms are omitted for clarity. Cuprous ion Cu(14) coincides with an inversion center in monoclinic *C*2/*c*.

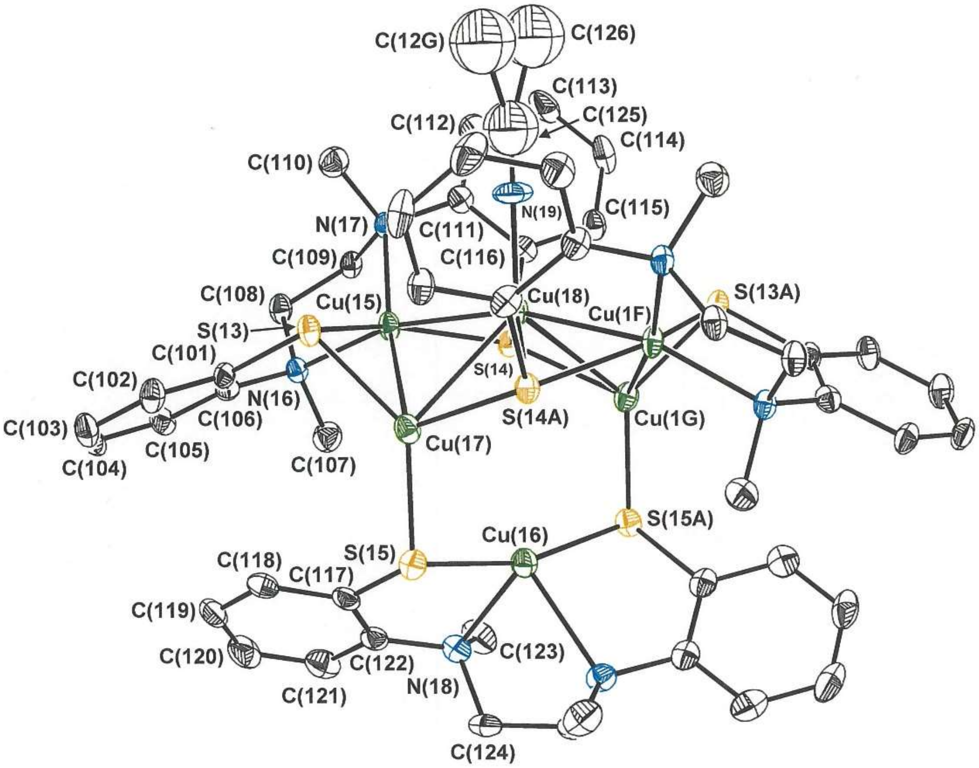

Figure S31. Thermal ellipsoid plot (50% probability level) with atom labeling for $[Cu_2(Cu\text{-}(L\text{-}N_2S_2))_3(Cu(N{\equiv}CMe))]$, **17** (data set JPD776). All hydrogen atoms are omitted for clarity. This molecule resides upon a C_2 axis in C_2/c such that only half of it is crystallographically unique.

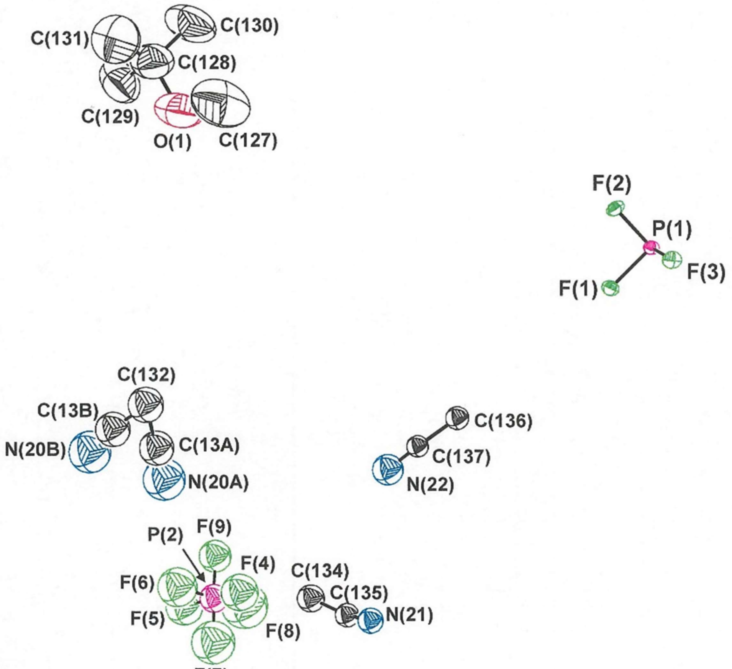

Figure S32. Thermal ellipsoid plot (50% probability level) with complete atom labeling for the $[PF_6]^{1-}$ counteranions and the interstitial solvent in $[[Cu_2(Cu\text{-}L\text{-}N_2S_2)_3(Cu(NCMe))]_2Cu][PF_6]\cdot\frac{1}{2}[Cu_2(Cu\text{-}L\text{-}N_2S_2)_3(Cu(NCMe))]\cdot{}^tBuOMe\cdot 2MeCN$ (data set JPD776). All hydrogen atoms are omitted for clarity.

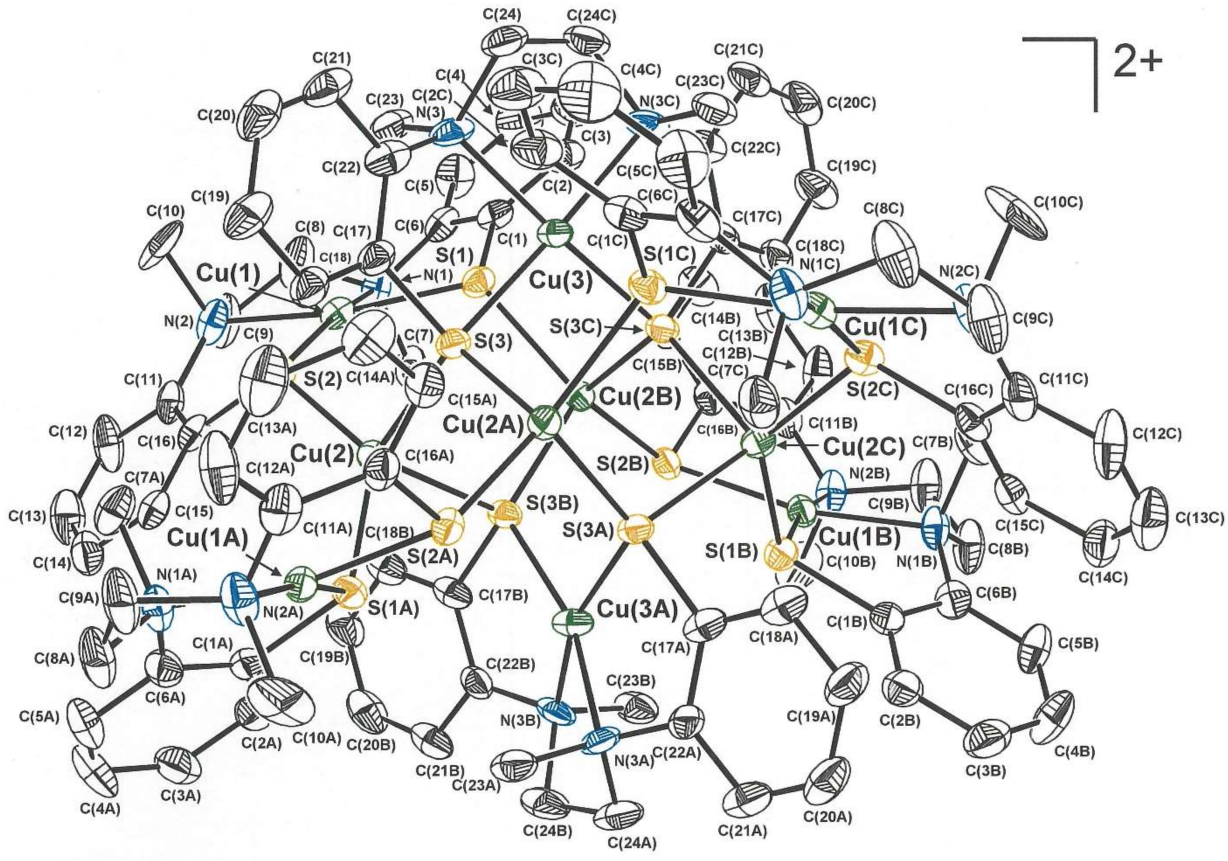

Figure S33. Thermal ellipsoid plot (50% probability level) with complete atom labeling for $[Cu_{10}(L\text{-}N_2S_2)_6]^{2+}$ in the structure of $[Cu_{10}(L\text{-}N_2S_2)_6][PF_6]_2 \cdot 2MeCN$. All hydrogen atoms are omitted for clarity. The dication resides upon an S_4 axis in $I4_1/a$ that coincides with the Cu(3)···Cu(3A) axis.

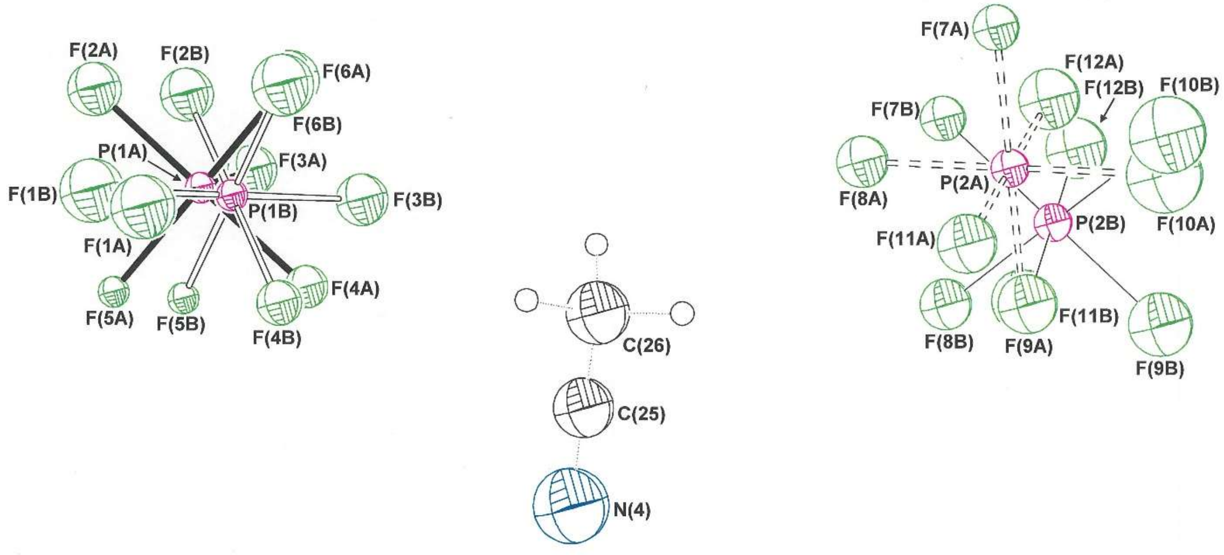

Figure S34. Thermal ellipsoid plot (50% probability level) with complete atom labeling for the $[PF_6]^{1-}$ counteranion and the interstitial solvent in the structure of $[Cu_{10}(L\text{-}N_2S_2)_6][PF_6]_2 \cdot 2MeCN$. All hydrogen atoms are omitted for clarity.

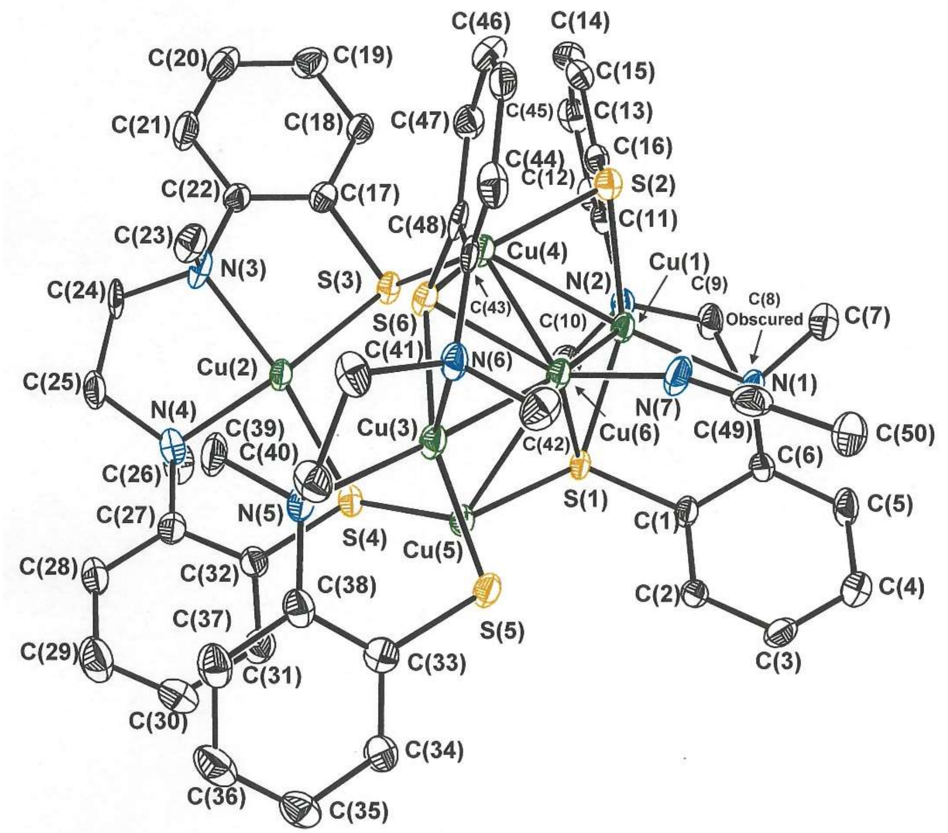

Figure S35. Thermal ellipsoid plot (50% probability level) with complete atom labeling for the cluster cation $[(Cu_2(Cu\text{-}L\text{-}N_2S_2)_3)(Cu(N{\equiv}CMe))]^+$ in $[(Cu_2(Cu\text{-}L\text{-}N_2S_2)_3)(Cu(N{\equiv}CMe))][PF_6]{\cdot}C_5H_{12}$. All hydrogen atoms are omitted for clarity.

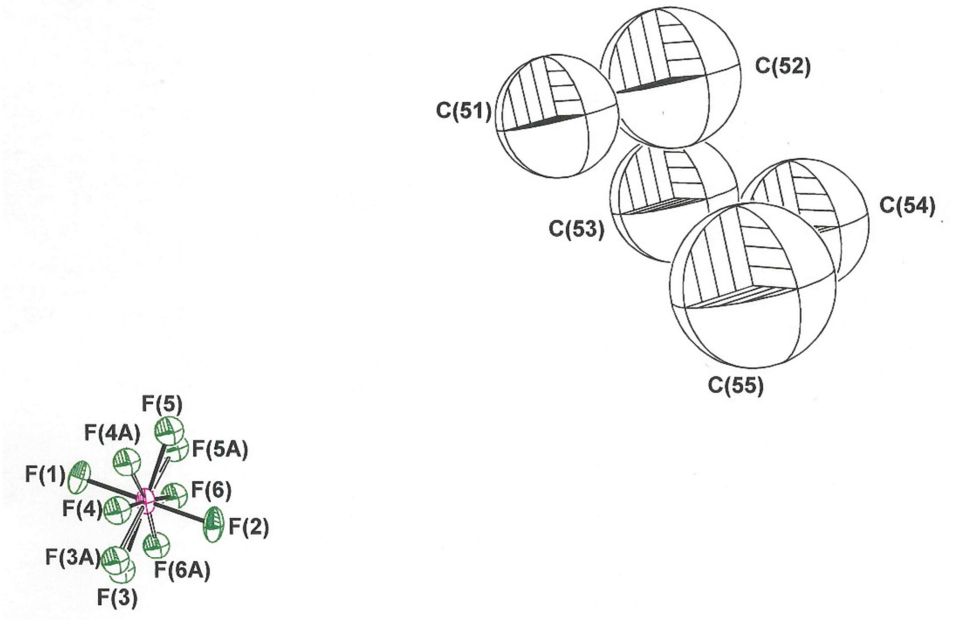

Figure S36. Thermal ellipsoid plot (50% probability level) with atom labeling for $[PF_6]^{1-}$ anion and the interstitial C_5H_{12} in $[(Cu_2(Cu\text{-}L\text{-}N_2S_2)_3)(Cu(N{\equiv}CMe))][PF_6]{\cdot}C_5H_{12}$. Hydrogen atoms are omitted. The $[PF_6]^{1-}$ anion is refined with a split atom model over two positions.

JPD-111

SAMPLE IDENTIFICATION

Name James P. Donahue
Dept. of Chemistry, Tulane University
6400 Freret Street
New Orleans, LA 70118-5698
City Zip

Date April 8, 2015

Analysis	Theory	% Found	
C	69.55%	69.80	
H	6.57%	6.80	
N	10.14%	10.28	
F	13.75%		

M.P./B.P.

Hygroscopic: Explosive:

Molecular Formula: $C_{16}H_{18}F_2N_2$

To Be Dried: No ☒ Yes ☐ at °C

ANALYZE for: C, H, N ☒ Single ☐ Duplicate

m/m Lab Established in 1986

MIDWEST MICROLAB, LLC
7212 N. SHADELAND AVE., SUITE 110 INDIANAPOLIS, IN 46250
PHONE (317) 849-6606 FAX (317) 849-8534

Analysis results may be emailed to donahue@tulane.edu .

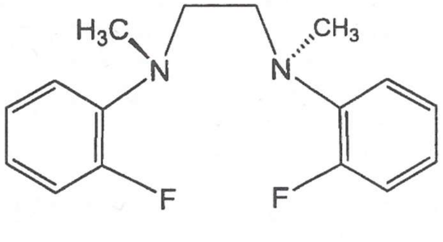

$C_{16}H_{18}N_2F_2$
MW = 276.3291 g/mol

Received: APR 13 2015 Completed:

It is suggested that at least 5 mg. of sample be supplied per determination. Liquid samples should be suitably protected by metal foil in the cap if submitted in a screw cap vial. Volatile substances would best be sealed in small ampoules. All samples will be returned for postal charges only.

Figure S37. Elemental analysis results from Midwest Microlab of Indianapolis, Indiana, for *N*[1],*N*[2]-bis(2-fluorophenyl)-*N*[1],*N*[2]-dimethylethane-1,2-diamine, **7**.

James P. Donahue
Dept. of Chemistry, Tulane University
6400 Freret Street
Name New Orleans, LA 70118-5698
City Zip

JPD-117
SAMPLE IDENTIFICATION

Date November 16, 2015

Analysis	Theory	% Found	
C	69.18%	69.42	
H	8.71%	8.68	
N	6.72%	6.85	
S	15.39%		

M.P./B.P.

Hygroscopic: Explosive:

Molecular Formula:

To Be Dried: No ☒ Yes ☐ at °C

ANALYZE for: C, H, N ☒ Single ☐ Duplicate

m/m Lab Established in 1986
MIDWEST MICROLAB, LLC
7212 N. SHADELAND AVE., SUITE 110 INDIANAPOLIS, IN 46250
PHONE (317) 849-6606 FAX (317) 849-8534

Analysis results may be emailed to donahue@tulane.edu .

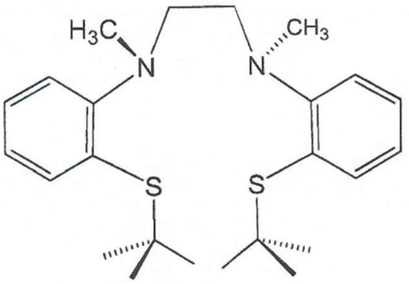

$C_{24}H_{36}N_2S_2$
MW = 416.69524 g/mol

Received: NOV 19 2015

Completed:

It is suggested that at least 5 mg. of sample be supplied per determination. Liquid samples should be suitably protected by metal foil in the cap if submitted in a screw cap vial. Volatile substances would best be sealed in small ampoules. All samples will be returned for postal charges only.

Figure S38. Elemental analysis results from Midwest Microlab of Indianapolis, Indiana, for *N*[1],*N*[2]-bis(2-([t]butylthio)phenyl)-*N*[1],*N*[2]-dimethylethane-1,2-diamine, **4b**.

James P. Donahue
Dept. of Chemistry, Tulane University
6400 Freret Street
Name New Orleans, LA 70118-5698
City Zip

JPD-133
SAMPLE IDENTIFICATION

Date June 22, 2016

Analysis	Theory	% Found	
C	47.06%	46.96	
H	4.44%	4.35	
N	6.86%	6.78	
S	15.70%		
Cu	25.94%		

M.P./B.P.

Hygroscopic: Explosive:

Molecular Formula:

To Be Dried: No ☒ Yes ☐ at °C

ANALYZE for: C, H, N ☒ Single ☐ Duplicate

m/m Lab Established in 1886

MIDWEST MICROLAB, LLC
7212 N. SHADELAND AVE., SUITE 110 INDIANAPOLIS, IN 46250
PHONE (317) 849-6606 FAX (317) 849-8534

Analysis results may be emailed to donahue@tulane.edu .

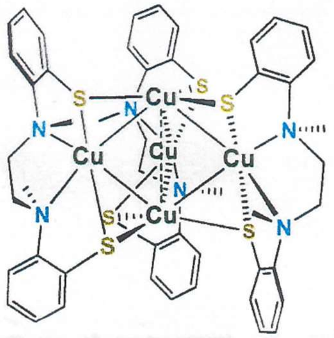

$C_{48}H_{54}N_6S_6Cu_5$
1225.12 g/mol

Received: JUN 24 2016 Completed:

It is suggested that at least 5 mg. of sample be supplied per determination. Liquid samples should be suitably protected by metal foil in the cap if submitted in a screw cap vial. Volatile substances would best be sealed in small ampoules. All samples will be returned for postal charges only.

Figure S39. Elemental analysis results from Midwest Microlab of Indianapolis, Indiana, for $[Cu_2(Cu(L\text{-}N_2(S^{Ar}{}_2)))_3]$, **19**.

MIKROLAB

Mikroanalytisches Laboratorium Kolbe

Professor James P. Donahue
Department of Chemistry
Tulane University
6400 Freret St.
New Orleans, Louisiana 70118-5698, USA

Address : Osterfelder Str. 3
D-46047 Oberhausen
Phone : +49 - (0)208 - 32502
Fax : +49 - (0)208 - 382314
Email : info@mikro-lab.de
Website : www.mikro-lab.de

Date : 05.12.2022

Sample Name		% C	% H	% N									V2O
JPD207	Found:	28,95	5,26	5,61									x
	Calculated:	29.35%	5.34%	5.71%									x

Kind regards

Patrick Springer

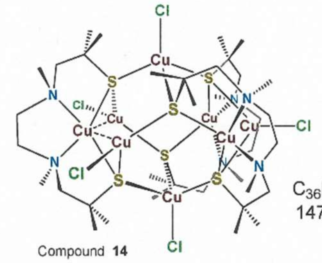

$C_{36}H_{78}N_6S_6Cu_8Cl_5$
1473.08452 g/mol

Compound **14**

Figure S40. Elemental analysis results from Kolbe Microanalytical Laboratory of Oberhaussen, Germany, for [(Cu(L-N$_2$(S^{Me_2})$_2$))$_3$(CuCl)$_5$], **14**.

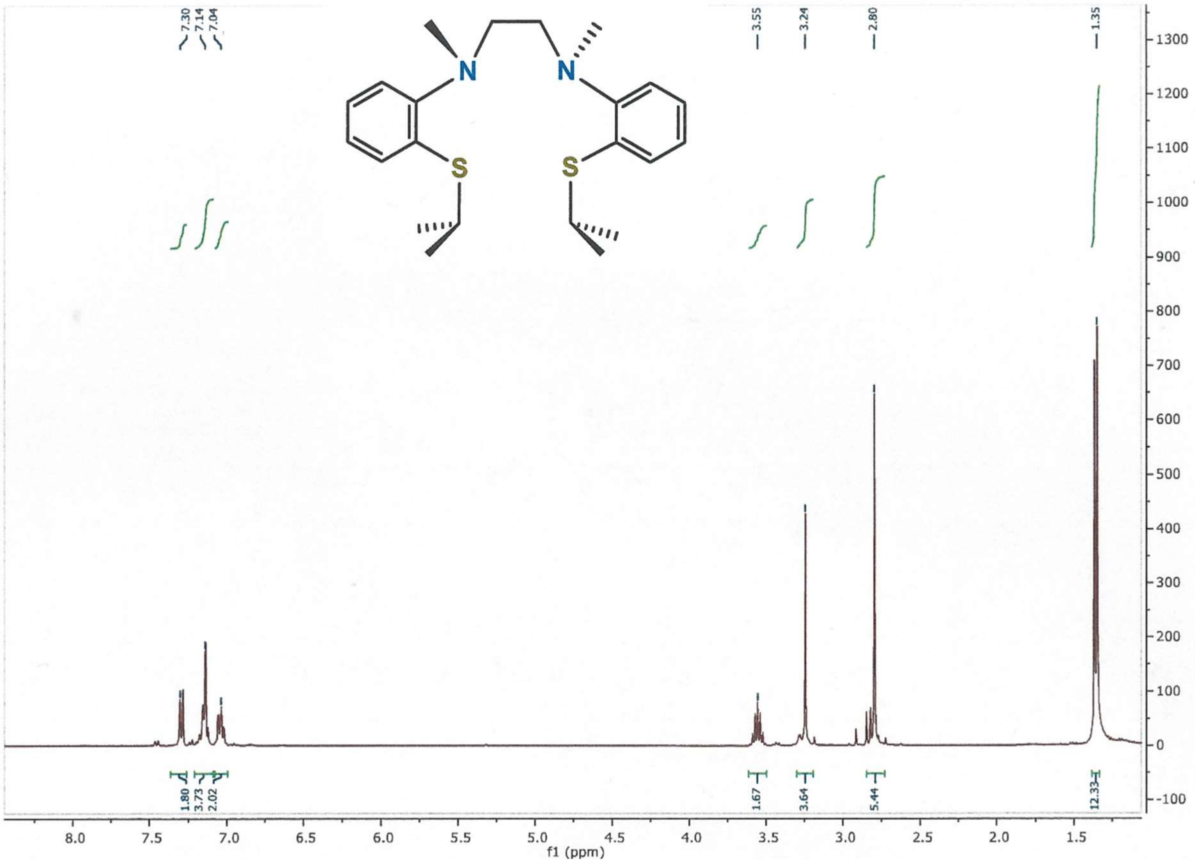

Figure S41. ^{1}H NMR spectrum (CD_2Cl_2) of *N[1]*,*N[2]*-bis(2-(^{i}propylthio)phenyl)-*N[1]*,*N[2]*-dimethylethane-1,2-diamine, **4a**.

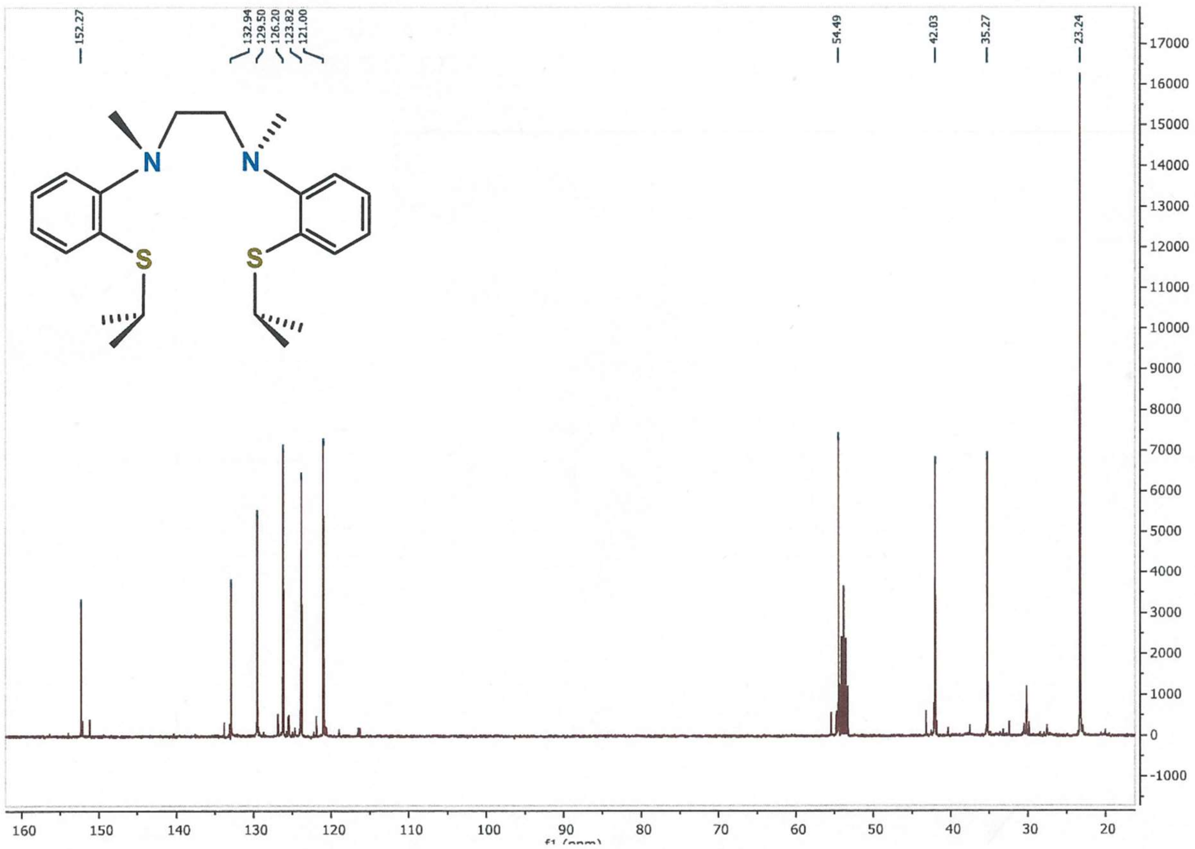

Figure S42. ^{13}C NMR spectrum (CD_2Cl_2) of N^1,N^2-bis(2-(^{i}propylthio)phenyl)-N^1,N^2-dimethylethane-1,2-diamine, **4a**.

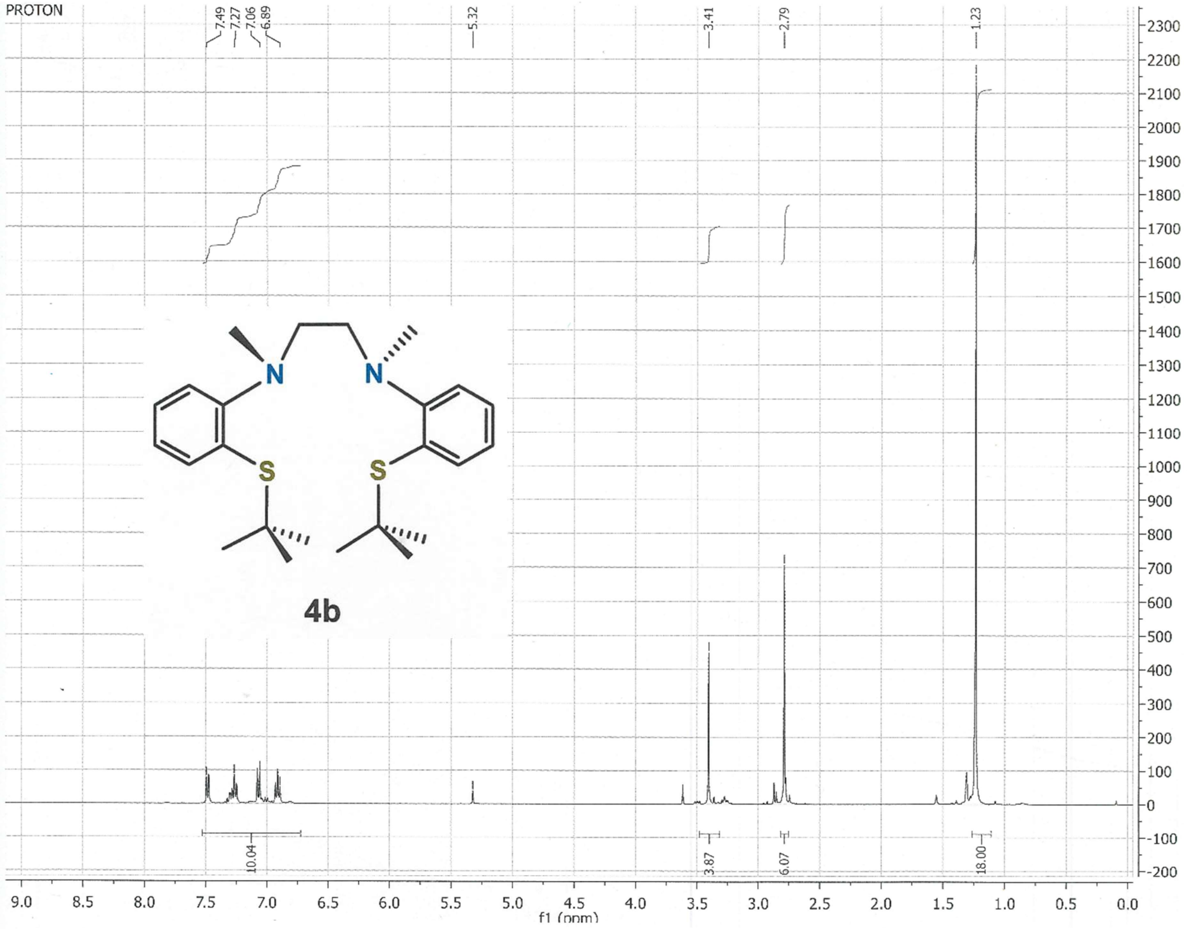

Figure S43. ^{1}H NMR spectrum (CD_2Cl_2) of *N[1]*,*N[2]*-bis(2-([t]butylthio)phenyl)-*N[1]*,*N[2]*-dimethylethane-1,2-diamine, **4b**.

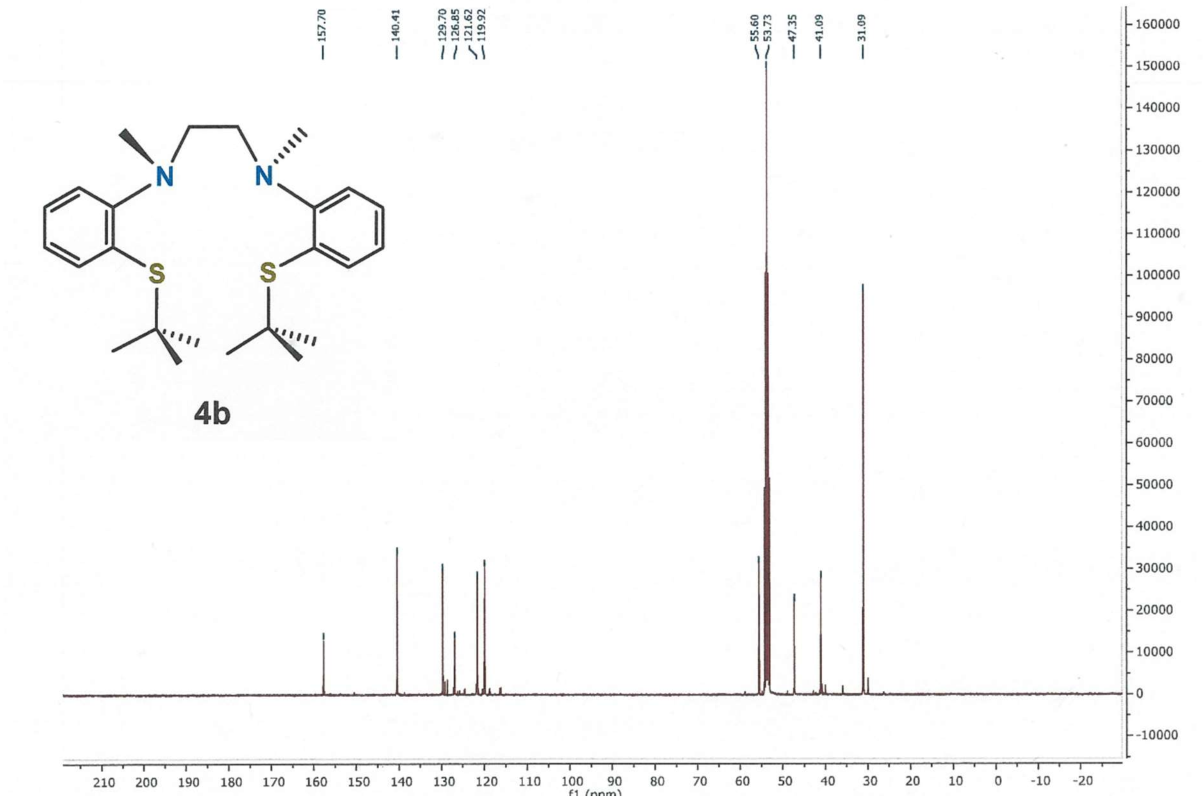

Figure S44. ^{13}C NMR spectrum (CD_2Cl_2) of *N*[1],*N*[2]-bis(2-(^{t}butylthio)phenyl)-*N*[1],*N*[2]-dimethylethane-1,2-diamine, **4b**.

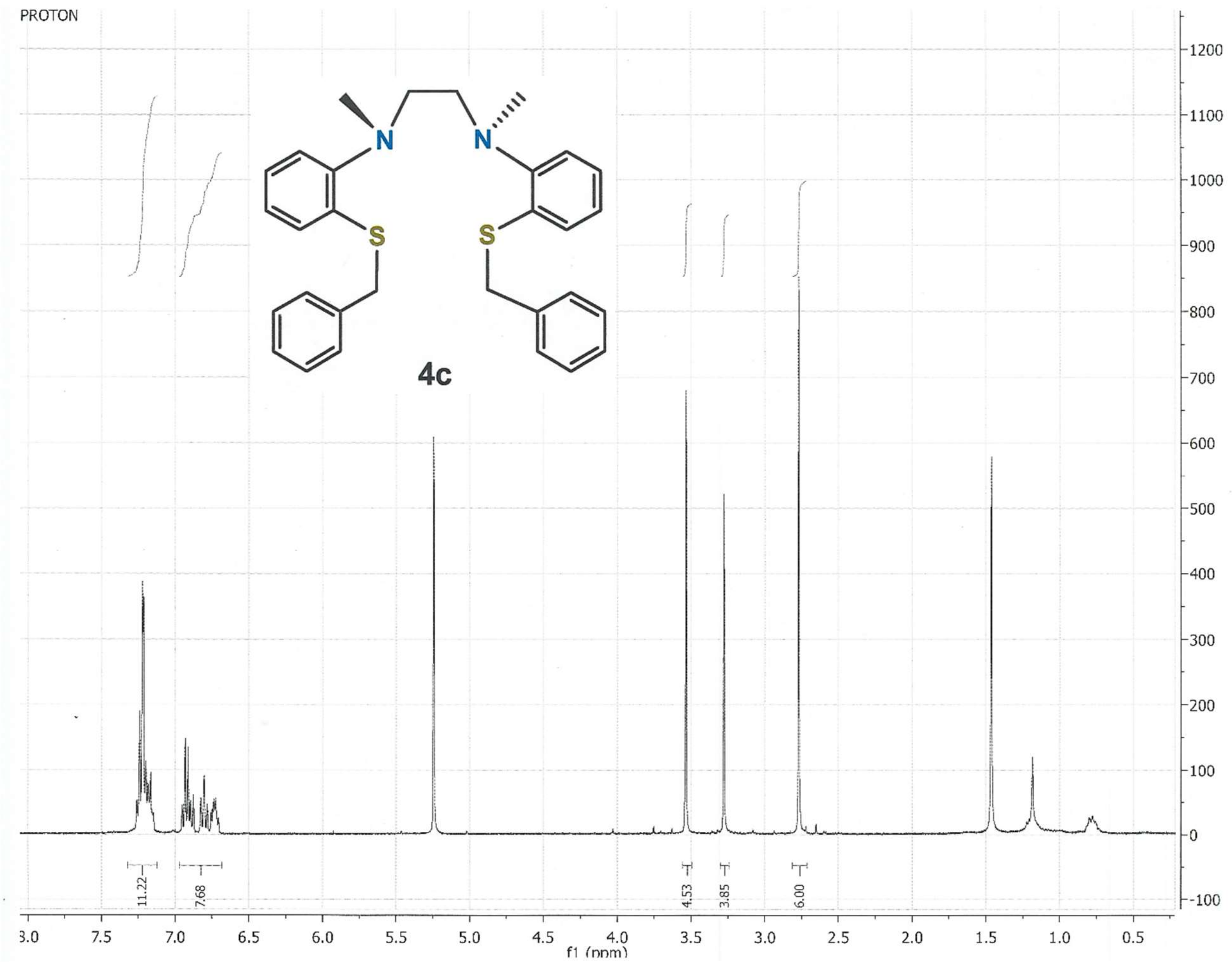

Figure S45. ^{1}H NMR spectrum (CD_2Cl_2) of *N[1]*,*N[2]*-bis(2-(benzylthio)phenyl)-*N[1]*,*N[2]*-dimethylethane-1,2-diamine, **4c**.

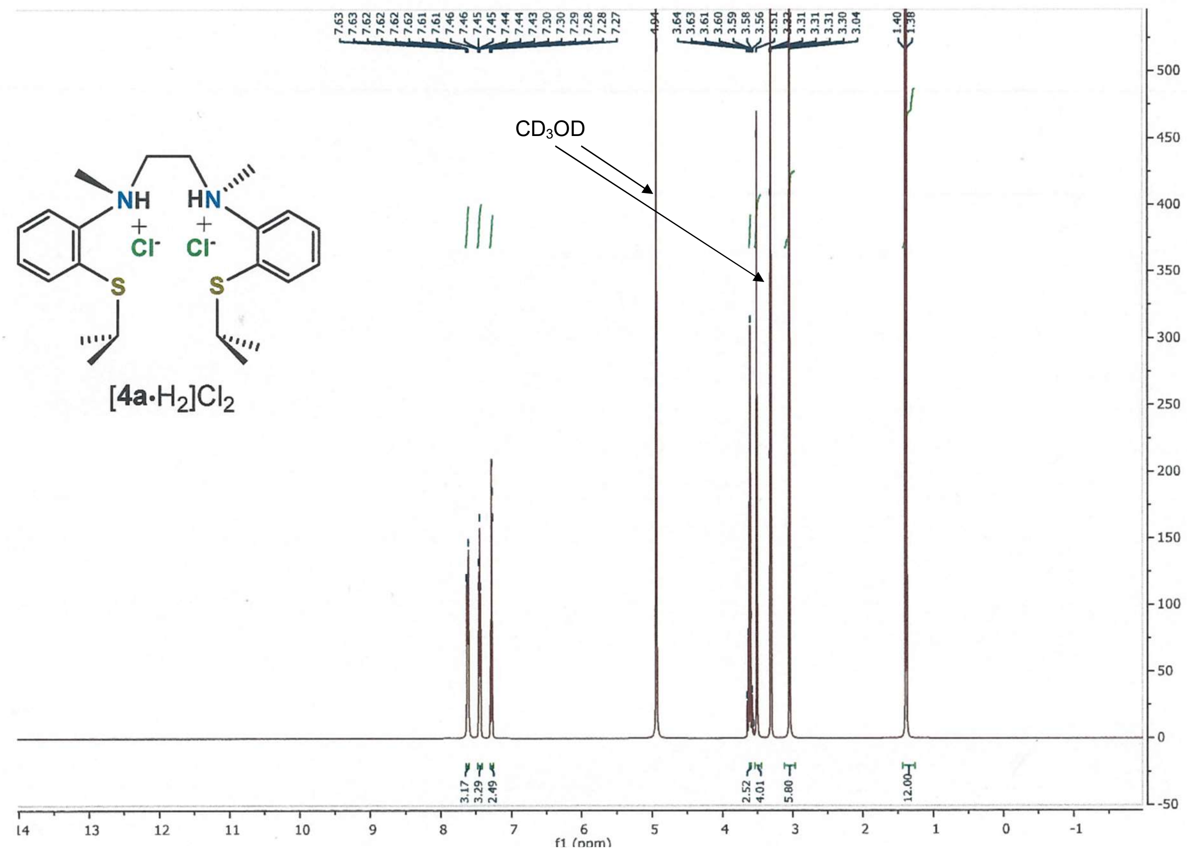

Figure S46. ^{1}H NMR spectrum (CD_3OD) of N^1,N^2-bis(2-(^{i}propylthio)phenyl)-N^1,N^2-dimethylethane-1,2-diamine bis(hydrochloride), [**4a**·H_2]Cl_2.

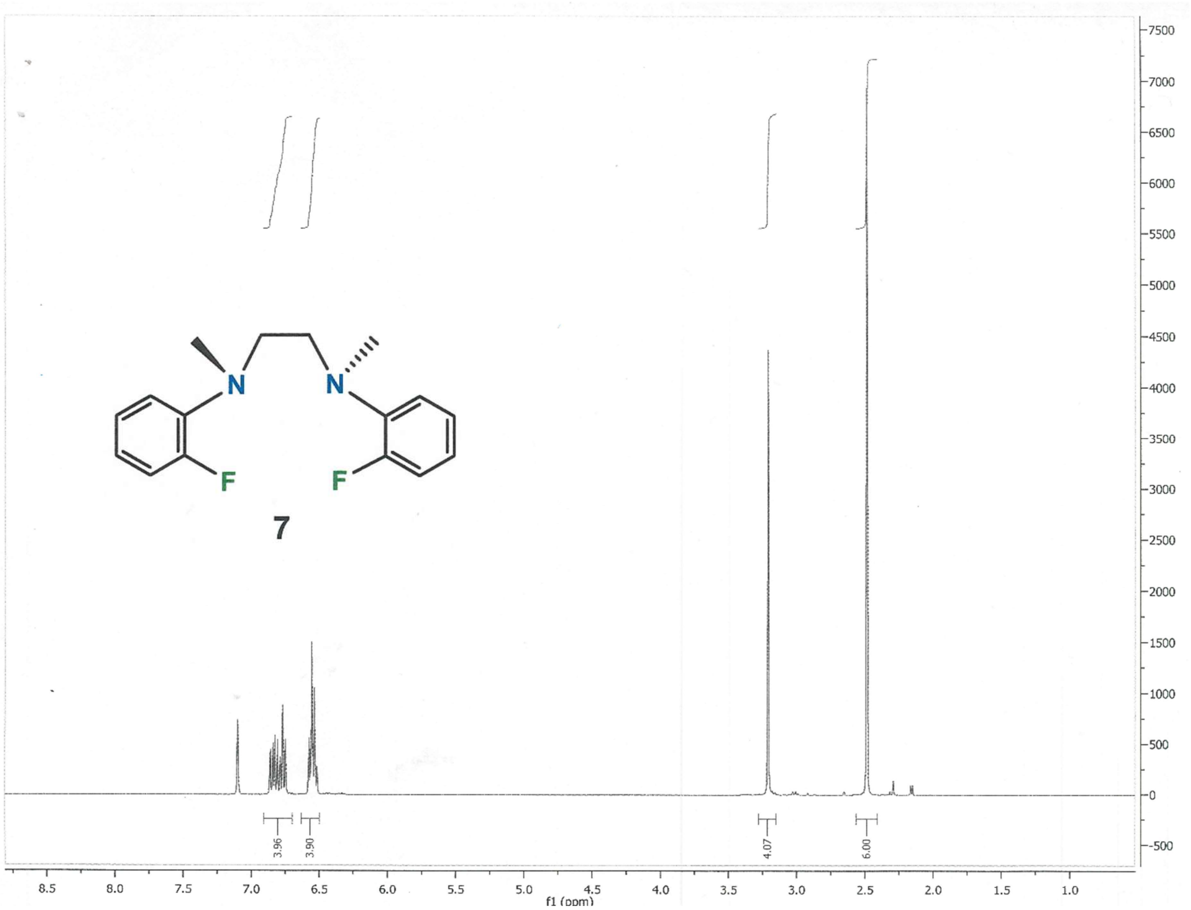

Figure S47. ^{1}H NMR spectrum (C_6D_6) of N^1,N^2-bis(2-fluorophenyl)-N^1,N^2-dimethylethane-1,2-diamine, **7**.

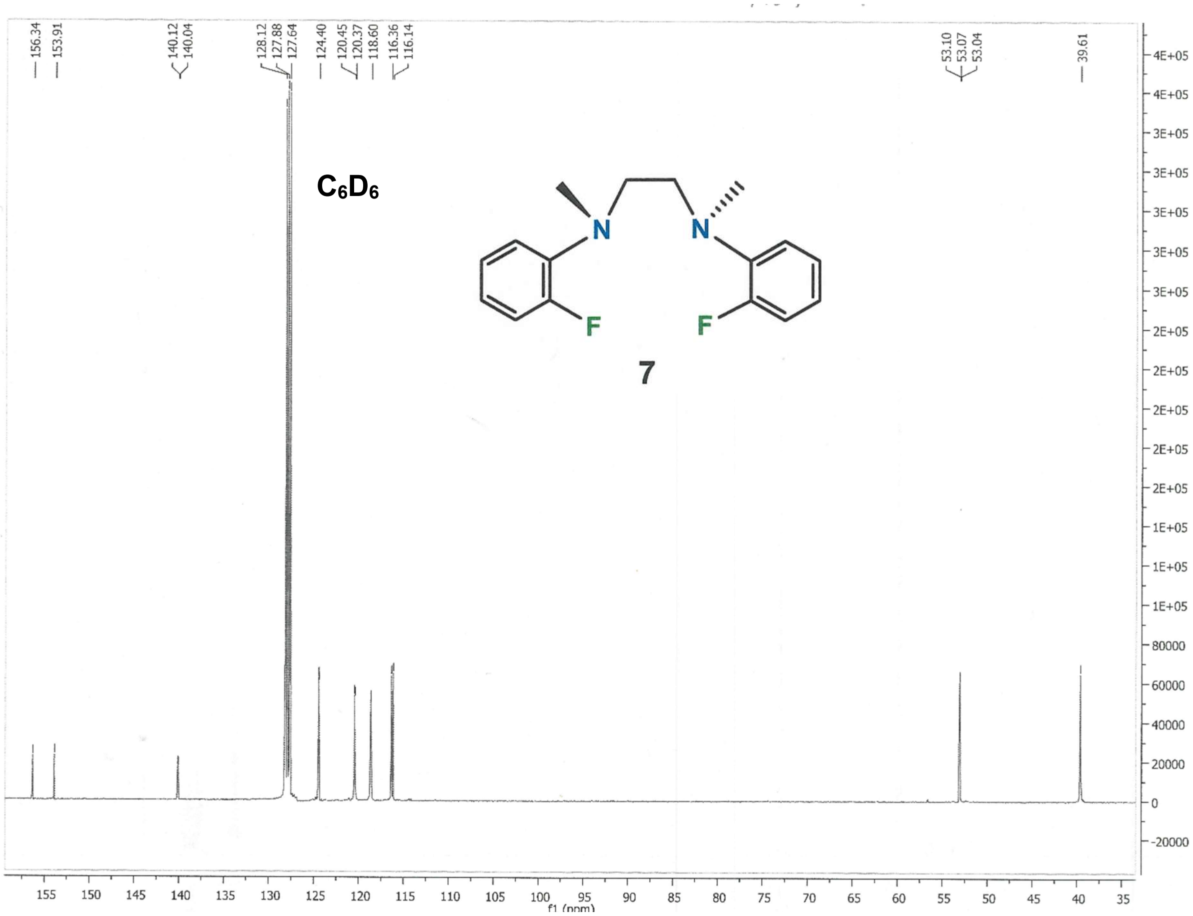

Figure S48. ^{13}C NMR spectrum (C_6D_6) of *N*[1],*N*[2]-bis(2-fluorophenyl)-*N*[1],*N*[2]-dimethylethane-1,2-diamine, **7**.

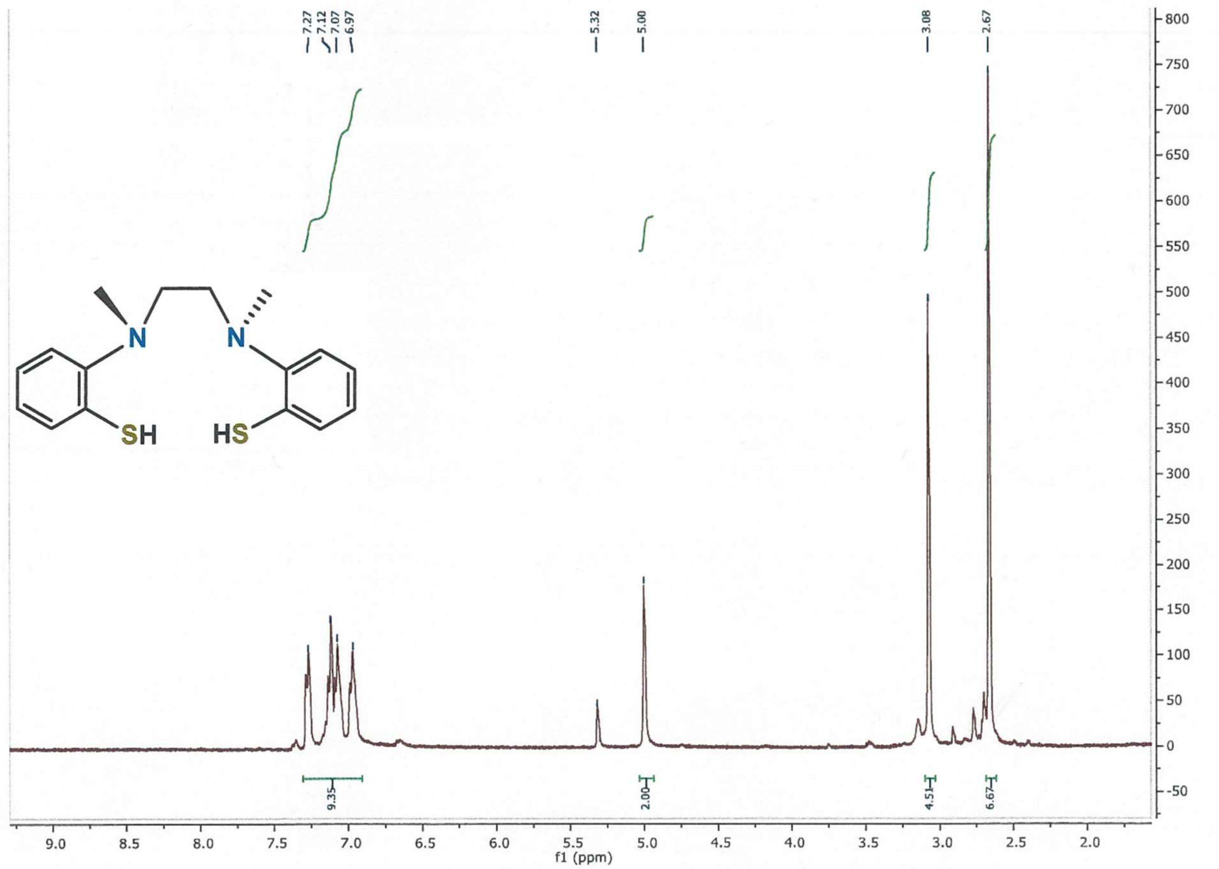

Figure S49. ^{1}H NMR spectrum (CD_2Cl_2) of *N¹*,*N²*-bis(2-mercaptophenyl)-*N¹*,*N²*-dimethylethane-1,2-diamine, **5**.

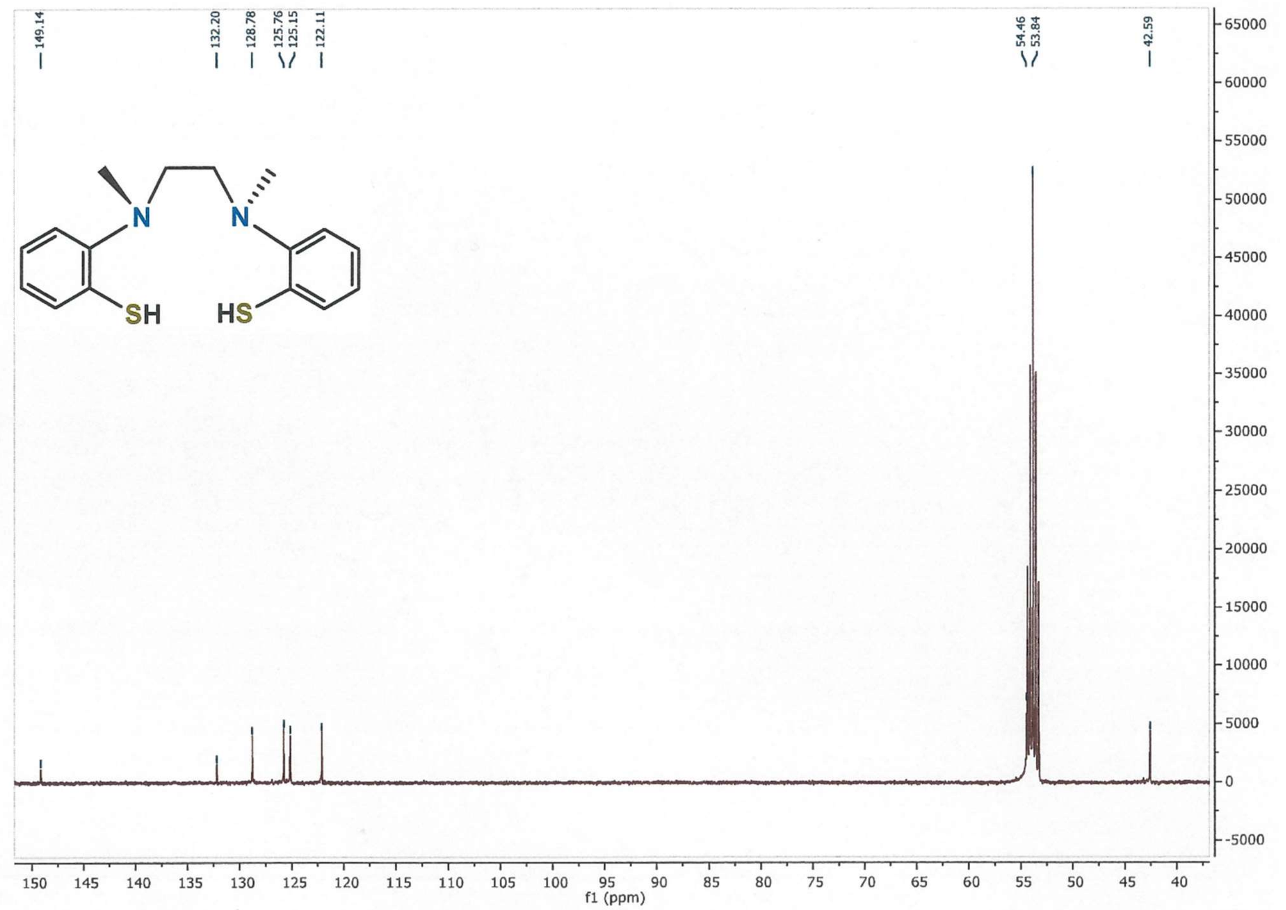

Figure S50. ^{13}C NMR spectrum (CD_2Cl_2) of *N*^{1},*N*^{2}-bis(2-mercaptophenyl)-*N*^{1},*N*^{2}-dimethylethane-1,2-diamine, **5**.

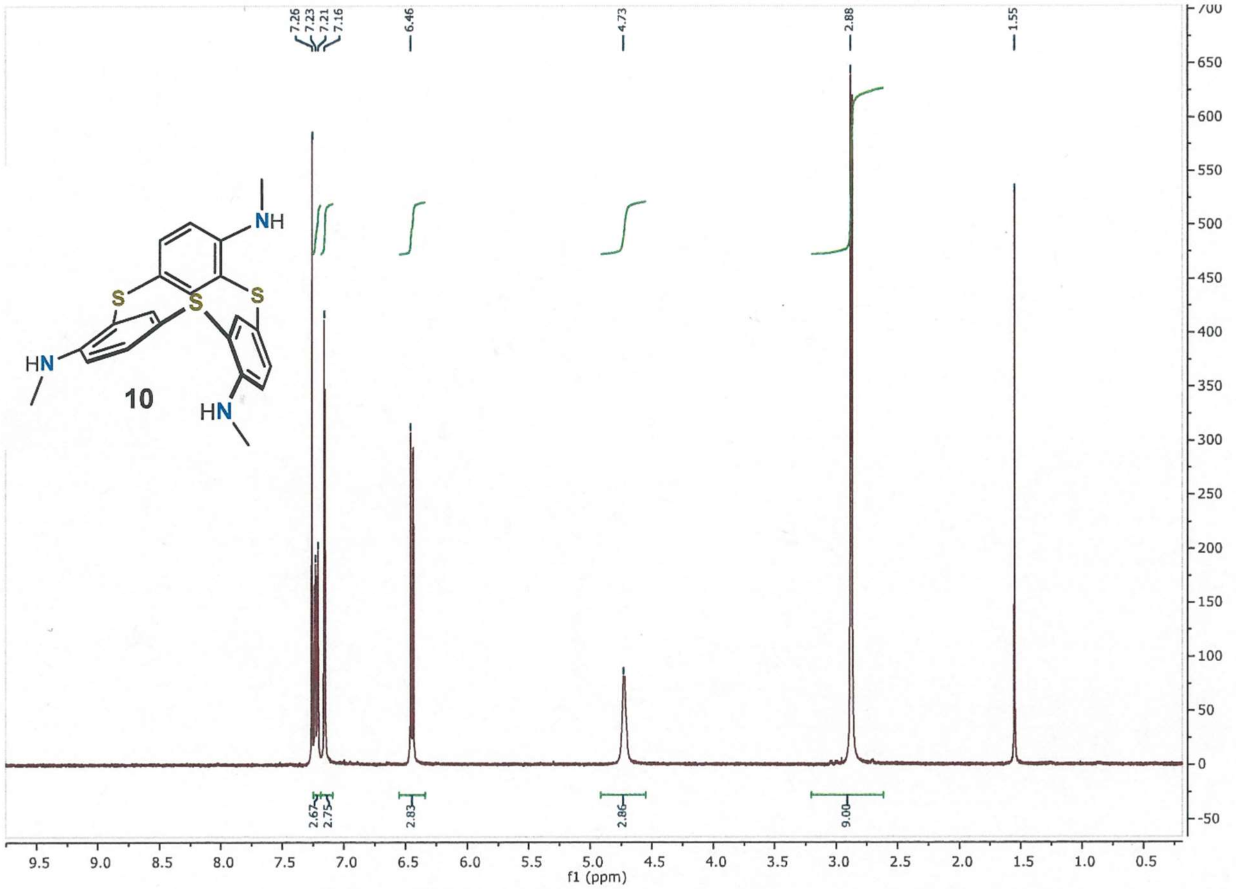

Figure S51. ^{1}H NMR spectrum ($CDCl_3$) of 2,8,14-trithiatetracyclo[13.3.1.1^{3,7}.1^{9,13}]heneicosa-1(19),3,5, 7(21),9,11,13(20),15,17-nonaene, 6,12,18-tris(*N*-methylamine), **10**.

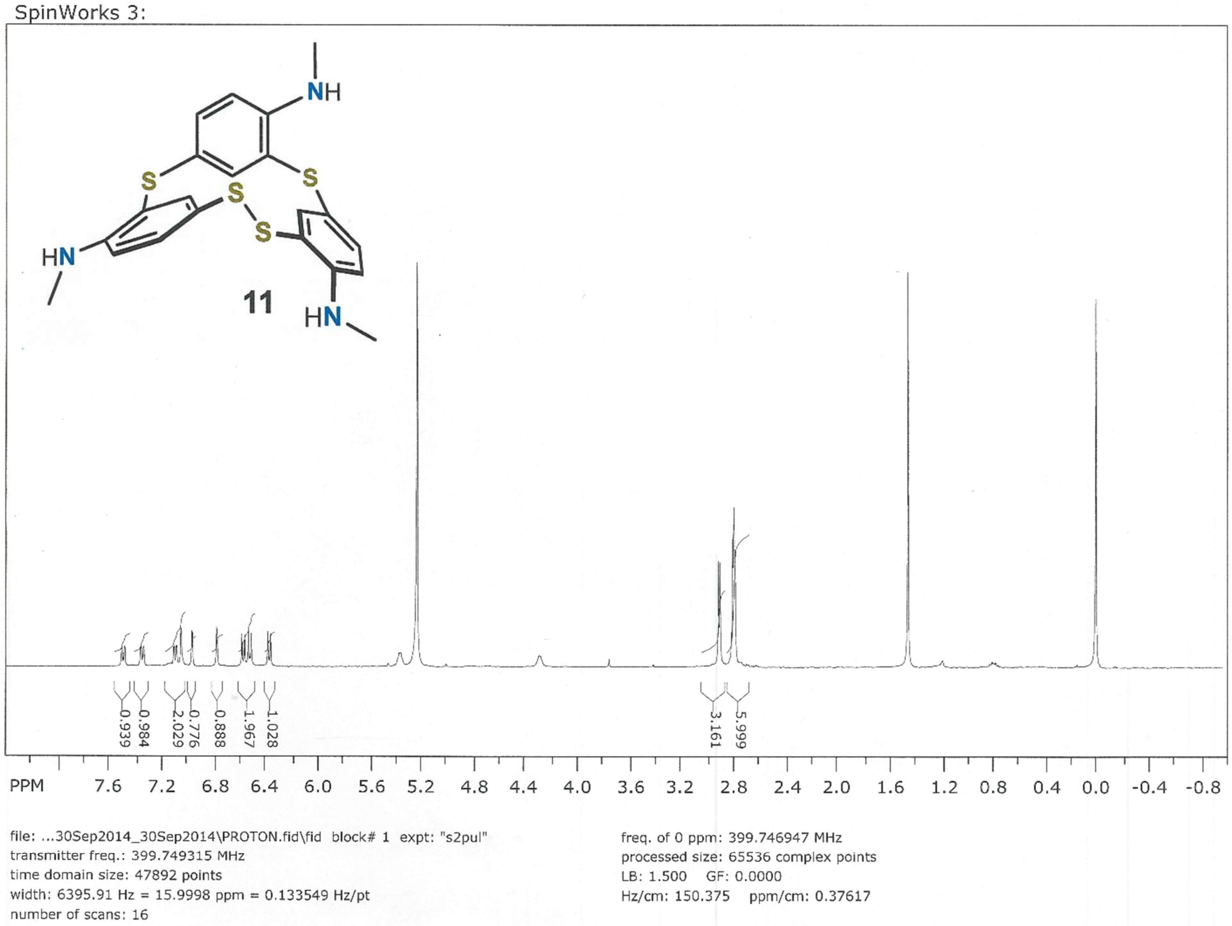

Figure S52. ^{1}H NMR spectrum (CD_2Cl_2) of 2,8,14,15-tetrathiatetracyclo[14.3.1.1^{3,7}.1^{9,13}]docosa-1(20),3,5,7(22),9,11,13(21),16,18-nonaene, 6,12,19-tri(*N*-methylamine)-, **11.**

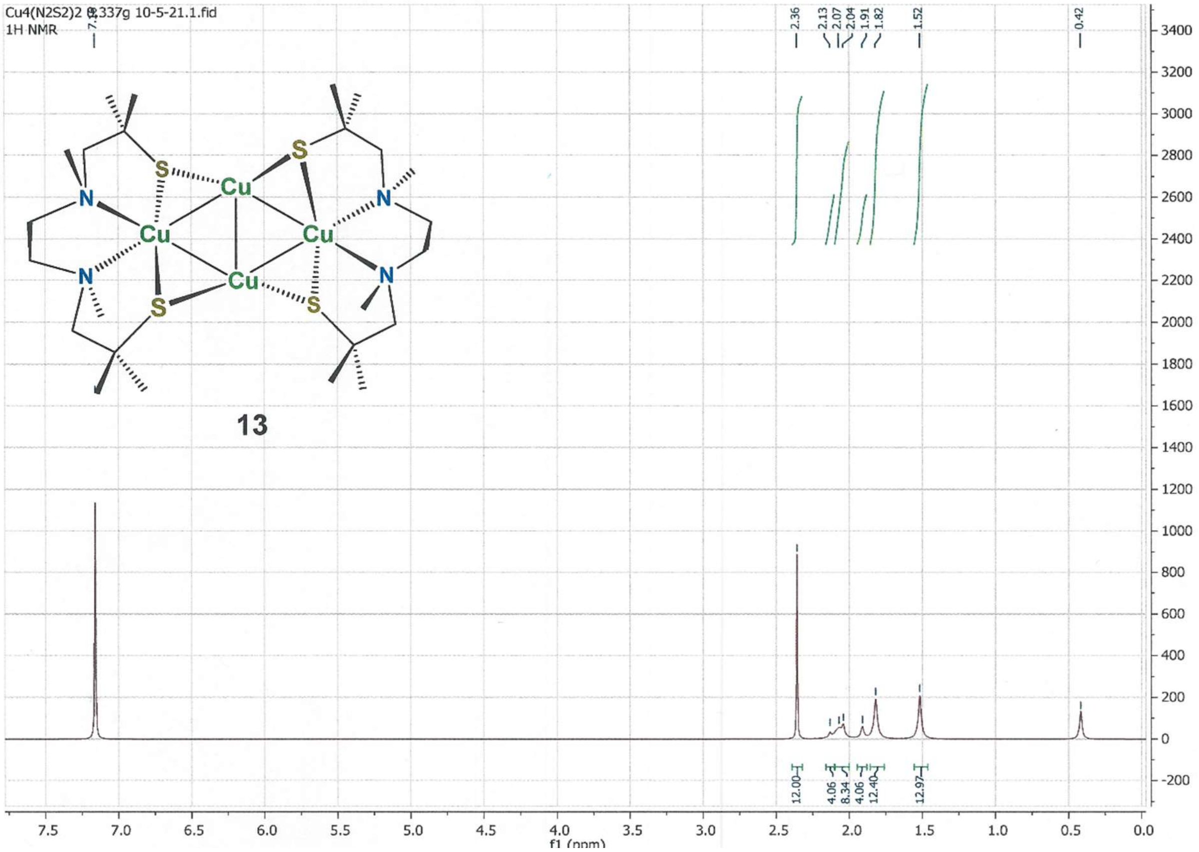

Figure S53. ^{1}H NMR spectrum (C_6D_6) of [(Cu(L-$N_2(S^{Me_2})_2$))$_2$Cu$_2$], **13**, full window.

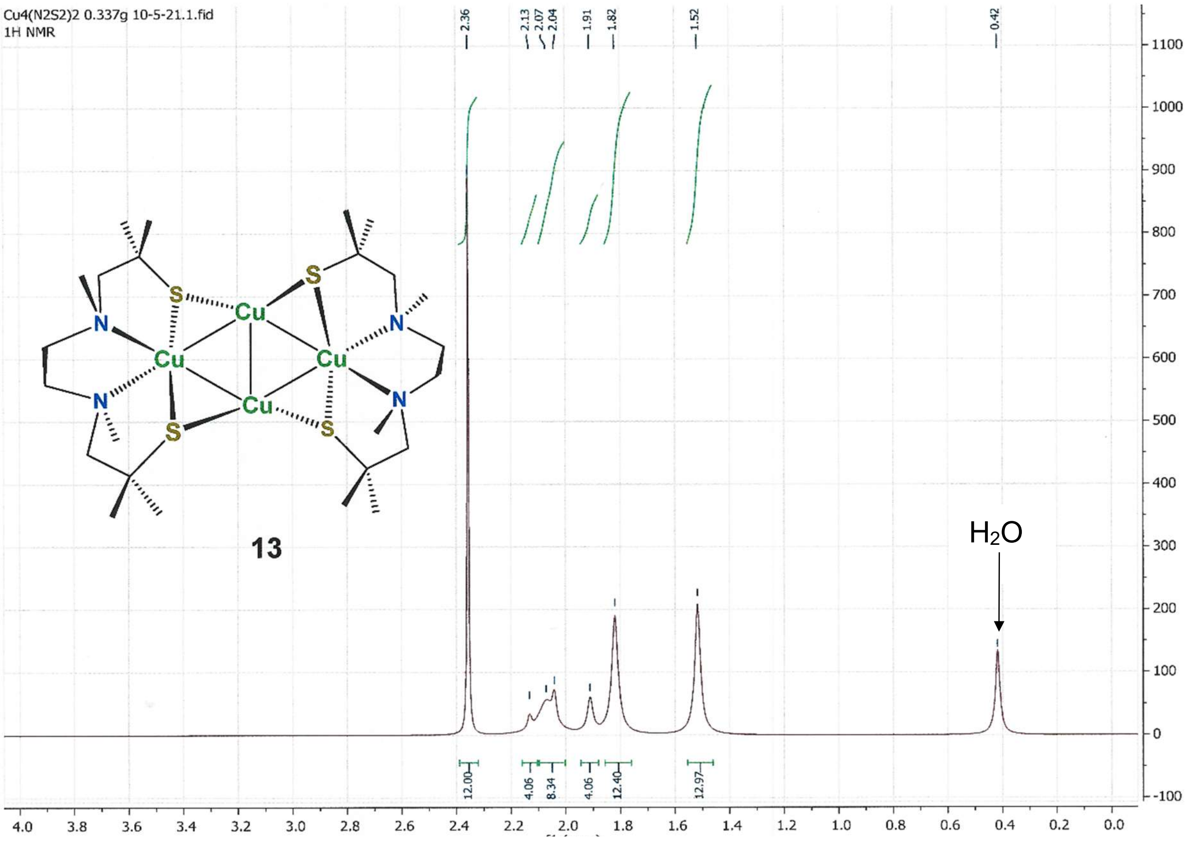

Figure S54. ^{1}H NMR spectrum (C_6D_6) of [(Cu(L-$N_2(S^{Me_2})_2$))$_2$Cu$_2$], **13**, 0.0-4.0 ppm window.

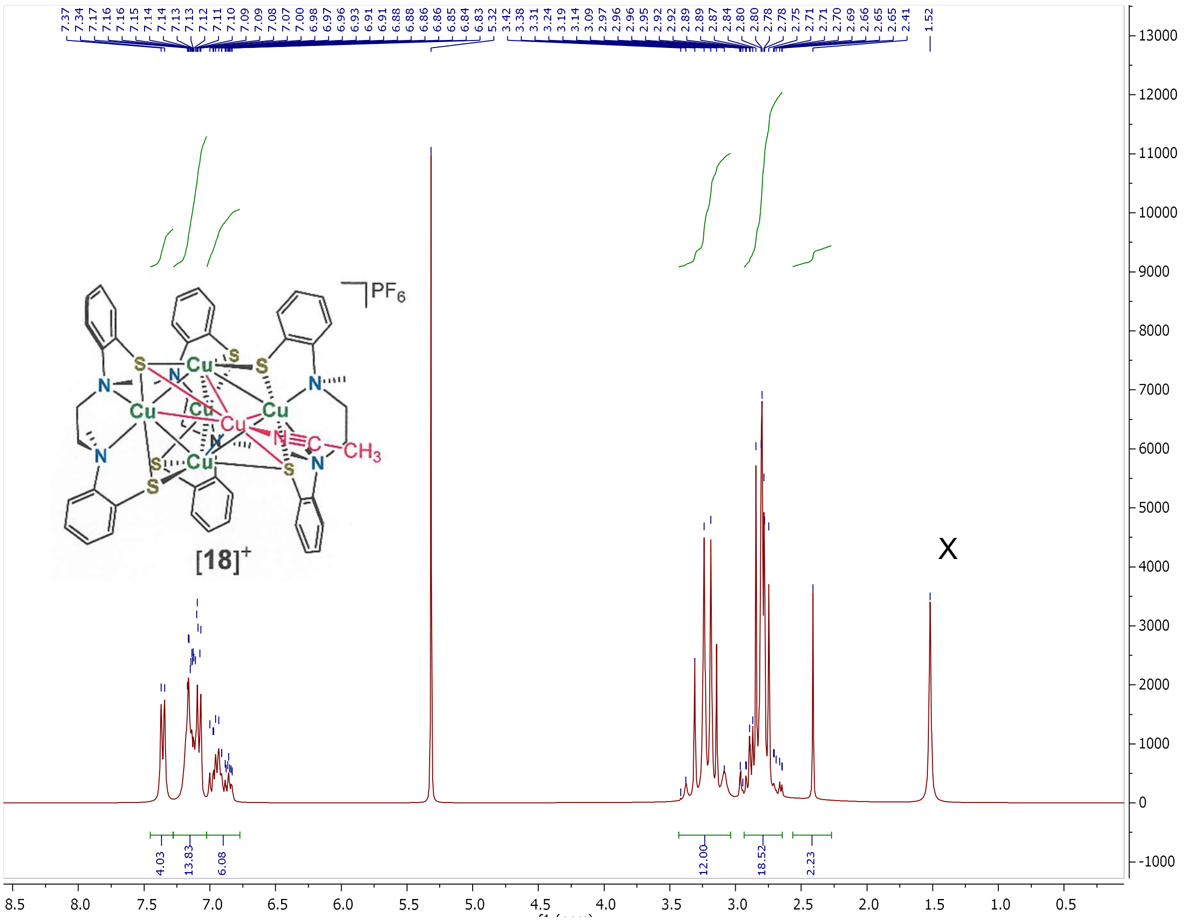

Figure S55. ^{1}H NMR spectrum in CD_2Cl_2 of $[Cu_2(Cu(L\text{-}N_2(S^{Ar}{}_2)))_3(Cu(N{\equiv}CMe))][PF_6]$, [**18**][$PF_6$].

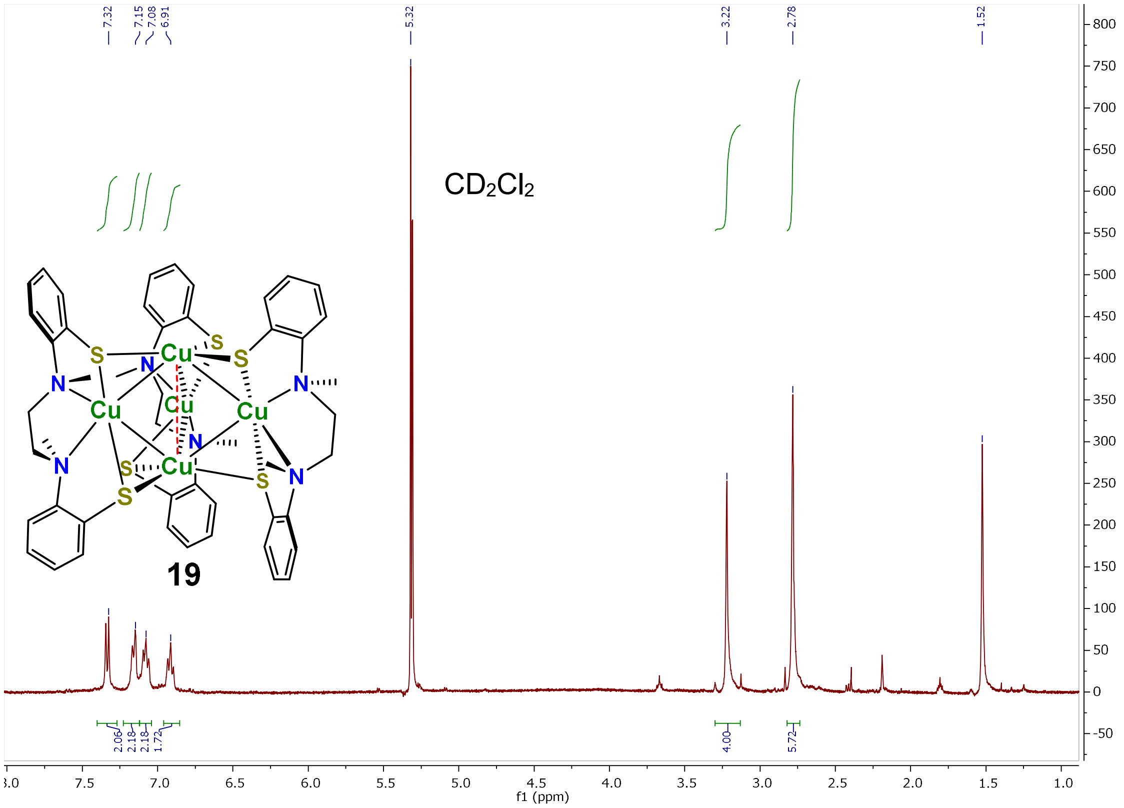

Figure S56. ^{1}H NMR spectrum of $[Cu_2(Cu(L\text{-}N_2S^{Ar}{}_2))_3]$ in CD_2Cl_2.

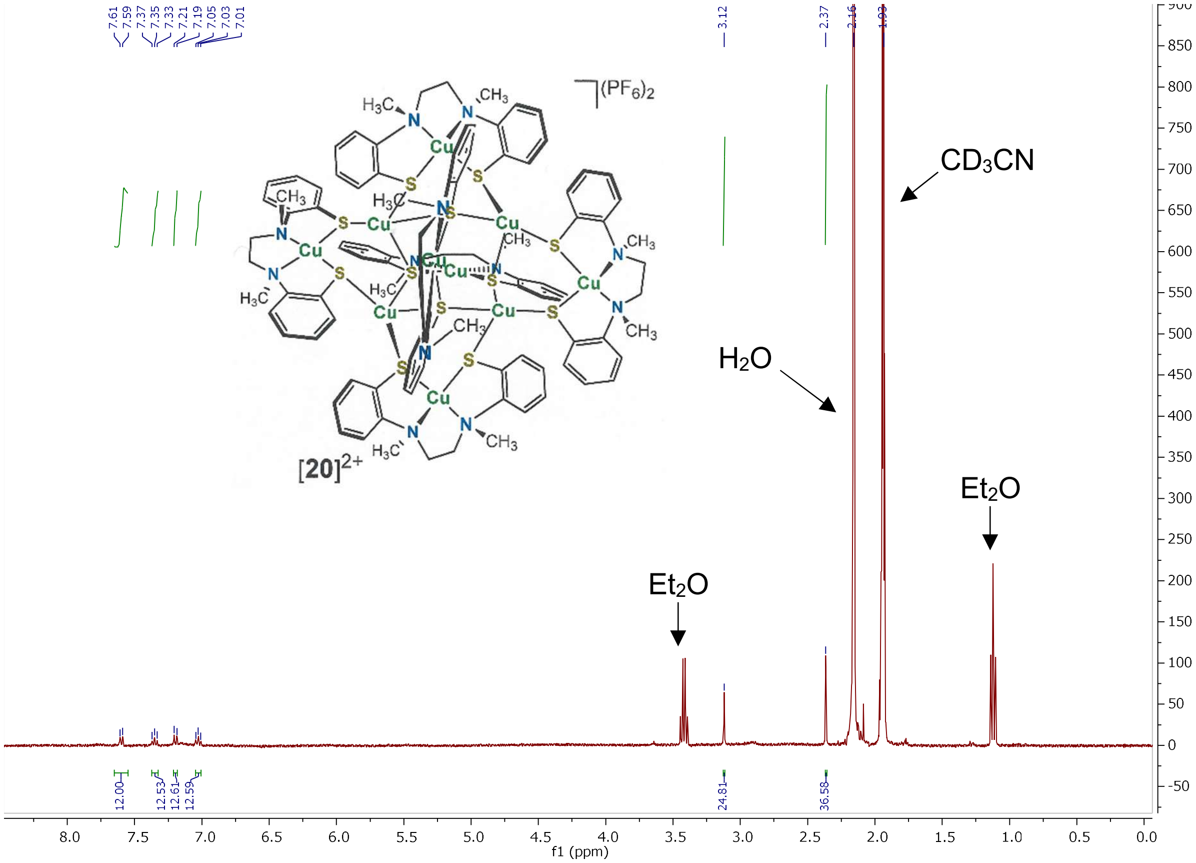

Figure S57. ^{1}H NMR spectrum of $[Cu_2(Cu(L\text{-}N_2S^{Ar}{}_2))_3][PF_6]_2$, **[20]**$[PF_6]_2$, in CD_3CN.

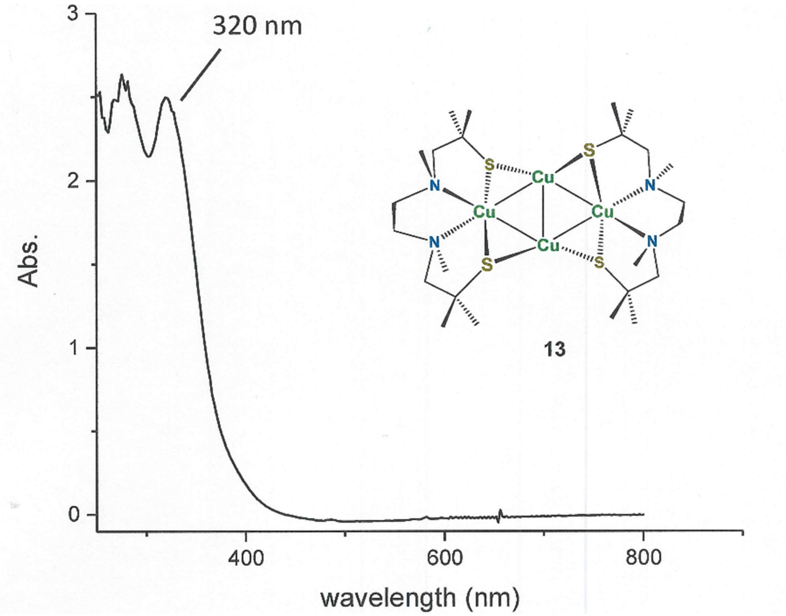

Figure S58. UV-vis spectrum of $[(Cu(L\text{-}N_2(S^{Me_2})_2))_2Cu_2]$, **13**, in CD_2Cl_2.

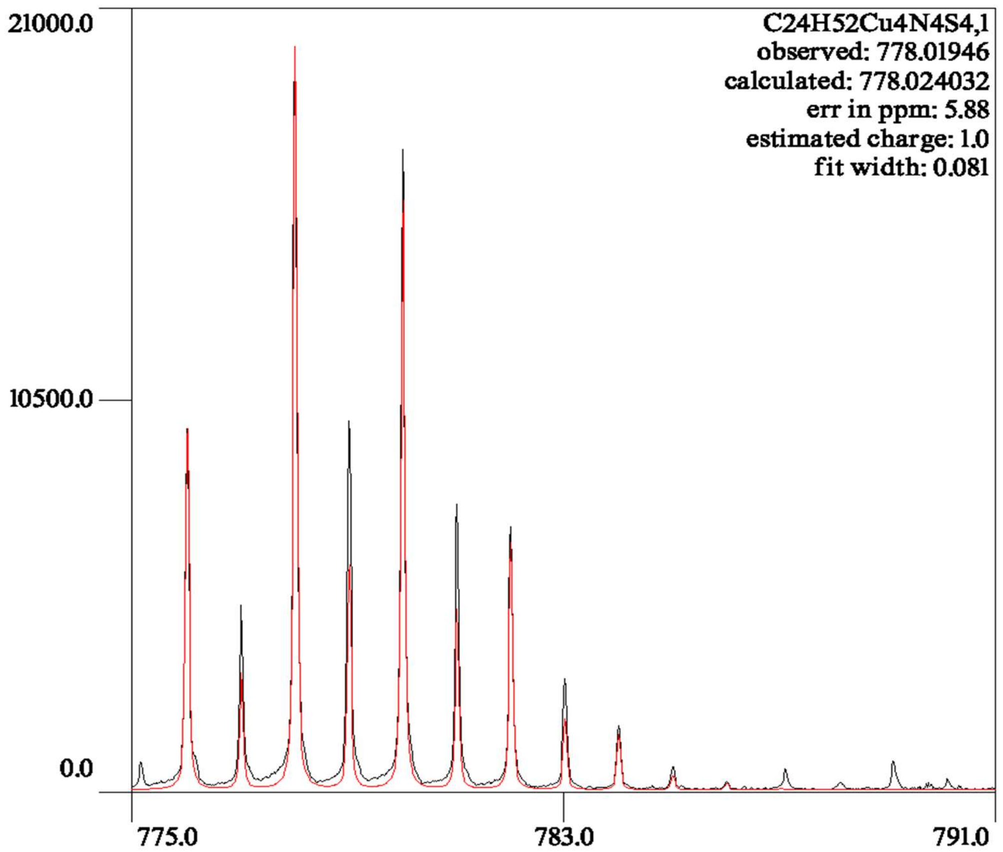

Figure S59. Mass spectrum (ESI) of $[(Cu(L\text{-}N_2(S^{Me_2})_2))_2Cu_2]$, **13**.

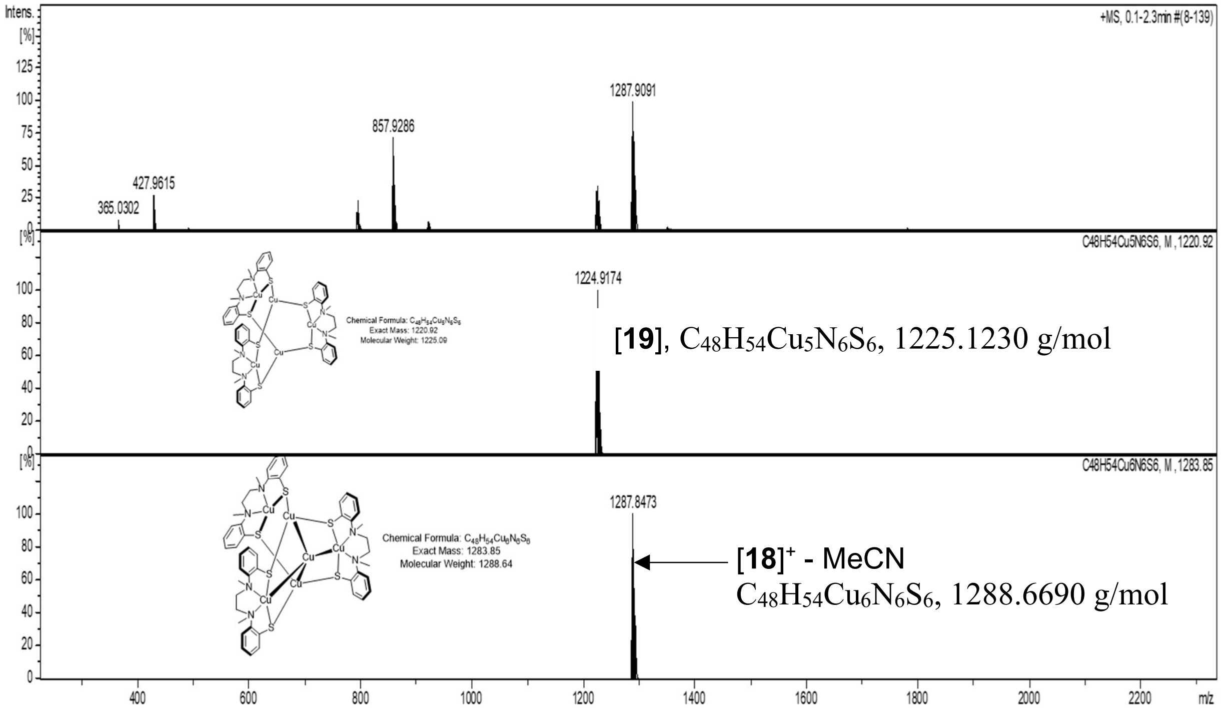

Figure S60. Mass spectra (ESI^+) of **19** and [**18**]$^+$.

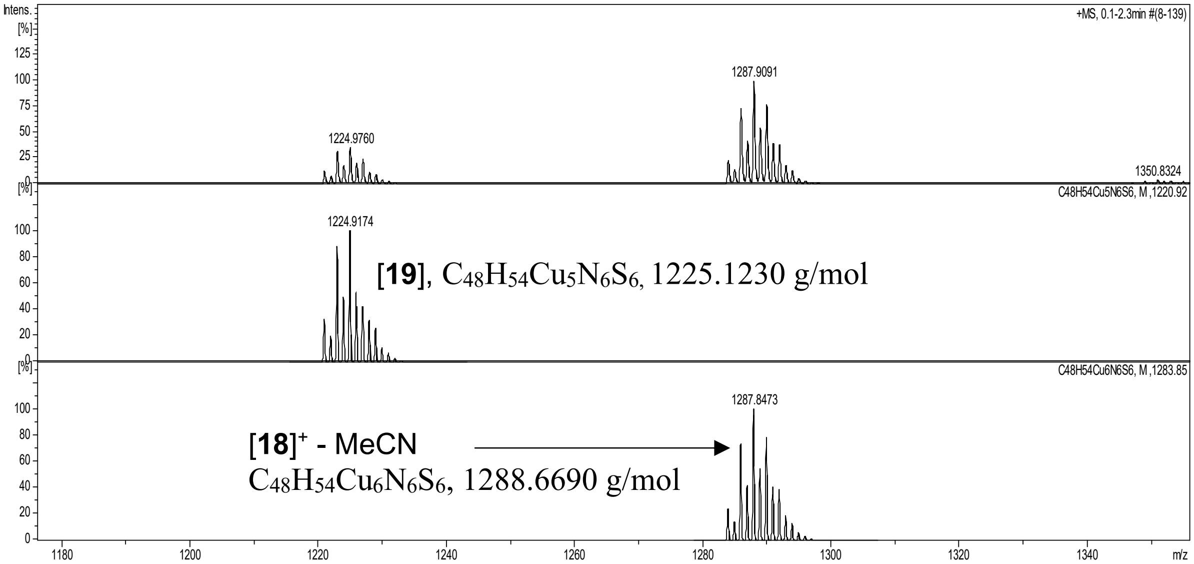

Figure S61. Zoomed in mass spectra (ESI^+) of **19** and [**18**]$^+$.

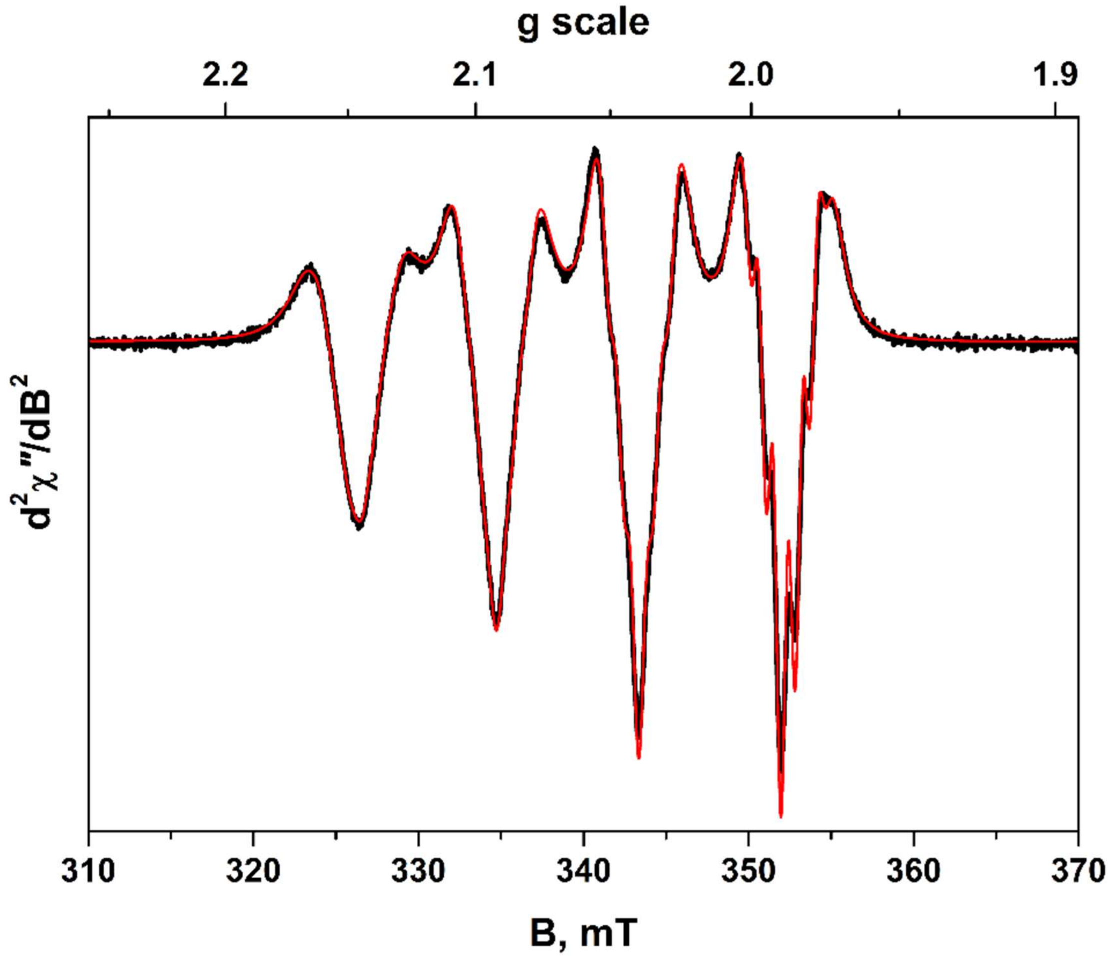

Figure S62. Second-derivative X-band EPR spectrum of **19** recorded in CH_2Cl_2 solution at 293 K (experimental conditions: frequency, 9.8017 GHz; power, 63 mW; modulation, 0.1 mT). Experimental data are represented by the black line; simulation is depicted by the red trace.

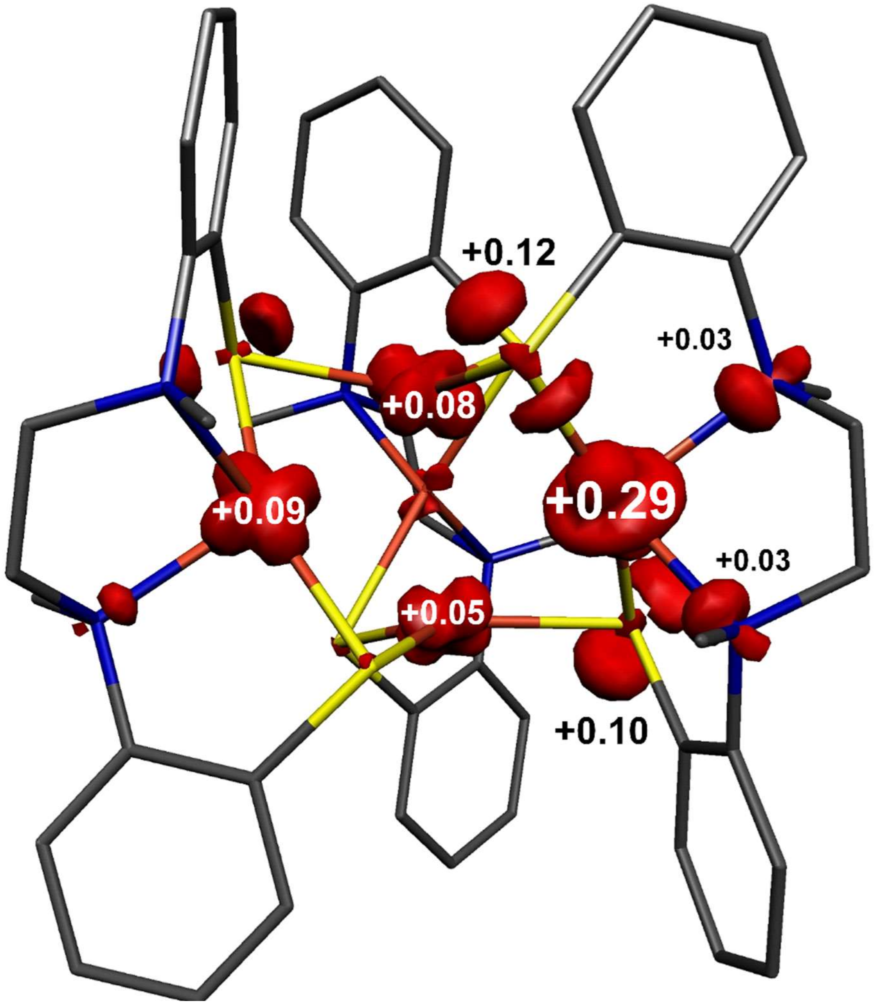

Figure S63. Mulliken spin density plot for **19** (red: α-spin).

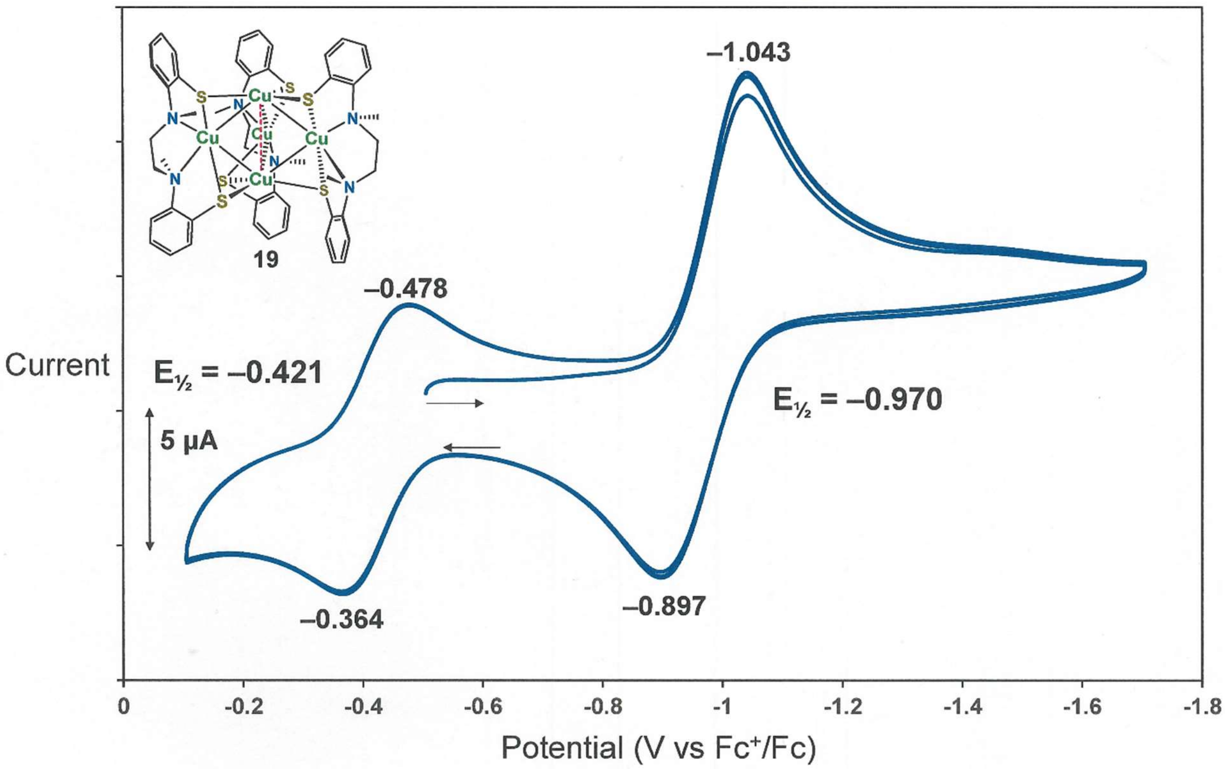

Figure S64. Cyclic voltammogram (100 mV/sec) of **19** in CH_2Cl_2 with scanning initiated in the cathodic direction. The supporting electrolyte was [$^{n}Bu_4N$][PF_6].

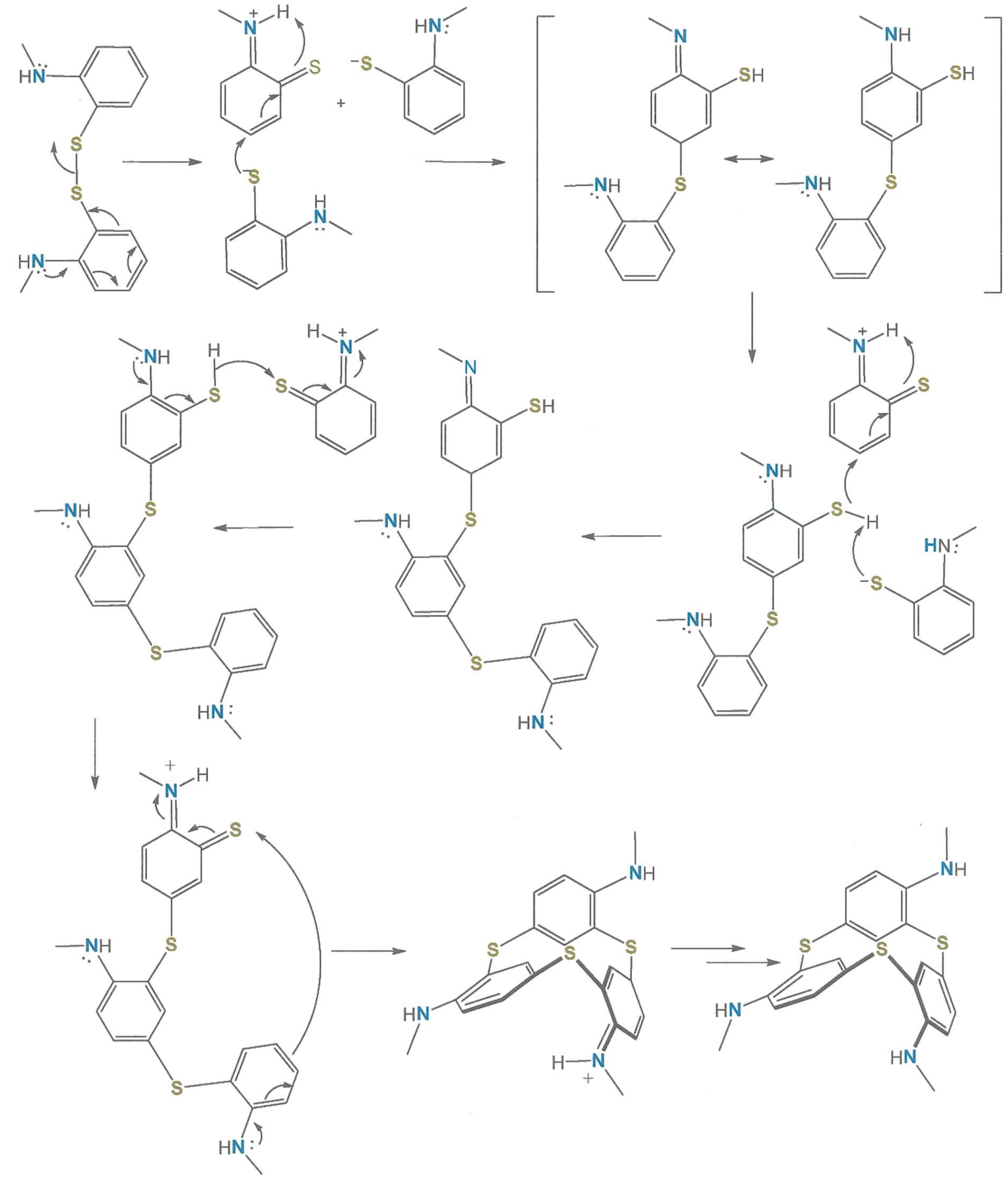

Scheme S1. A possible mechanism whereby compound **10** forms from disulfide **9**. A similar sequence would produce **11**.

References

(**1**) (**a**) *APEX2*, Version 2010.11-3, Bruker-AXS, Madison, Wisconsin, 2010. (**b**) *APEX2*, Bruker-AXS, Inc., Madison, Wisconsin, USA, 2013. (**c**) *APEX2*, Bruker-AXS, Inc., Madison, Wisconsin, USA, 2014. (**d**) *APEX2*, Bruker-AXS, Inc., Madison, Wisconsin, USA, 2015. (**e**) *APEX3*, Bruker-AXS, Inc., Madison, Wisconsin, USA, 2016. (**f**) *APEX3*, Bruker-AXS, LLC, Madison, Wisconsin, USA, 2020.

(**2**) (**a**) *SAINT*, Version 7.68A, Bruker AXS, Inc., Madison, Wisconsin, 2009. (**b**) *SAINT*, Bruker AXS, Inc., Madison, Wisconsin, 2013. (**c**) *SAINT*, Bruker AXS, Inc., Madison, Wisconsin, 2014. (**d**) *SAINT*, Bruker AXS, Inc., Madison, Wisconsin, 2015. (**e**) *SAINT*, Bruker AXS, Inc., Madison, Wisconsin, 2016. (**f**) *SAINT*, Bruker AXS, LLC, Madison, Wisconsin, 2020.

(**3**) Sheldrick, G, M. *CELL_NOW*, University of Göttingen, Göttingen, Germany, 2008.

(**4**) Sheldrick, G, M. *TWINABS*, University of Göttingen, Göttingen, Germany, 2009.

(**5**) (**a**) Sheldrick, G. M. *SADABS*, Version 2009/1, Universität Göttingen, Göttingen, Germany, 2009. (**b**) *SADABS*, Bruker AXS, Inc., Madison, Wisconsin, 2013. (**c**) *SADABS*, Bruker AXS, Inc., Madison, Wisconsin, 2014. (**d**) *SADABS*, Bruker AXS, Inc., Madison, Wisconsin, 2015. (**e**) *SADABS*, Bruker AXS, Inc., Madison, Wisconsin, 2016.

(**6**) Krause, L.; Herbst-Irmer, R.; Sheldrick, G.M.; Stalke; D. Comparison of Silver and Molybdenum Microfocus X-ray Sources for Single-Crystal Structure Determination. *J. Appl. Cryst.* **2015**, *48*, 3-10.

(**7**) (**a**) Sheldrick, G. M. *SHELXS-97*, University of Göttingen, Göttingen, Germany, 2008. (**b**) Sheldrick, G. M. A Short History of SHELX. *Acta Crystallogr., Sect. A: Foundations Adv.* **2008**, *64*, 112-122.

(**8**) (**a**) *SHELXT*, Bruker AXS, Inc., Madison, Wisconsin, 2008. (**b**) *SHELXT*, Bruker AXS, Inc., Madison, Wisconsin, 2013. (**c**) *SHELXT*, Bruker AXS, Inc., Madison, Wisconsin, 2014. (**d**) *SHELXT*, Bruker AXS, Inc., Madison, Wisconsin, 2015. (**e**) Sheldrick, G. M. *SHELXT* – Integrated Space-Group and Crystal-Structure Determination. *Acta Crystallogr., Sect. A: Foundations Adv.* **2015**, *71*, 3-8.

(**9**) (**a**) Sheldrick, G. M. A Short History of SHELX. *Acta Crystallogr., Sect. A* **2008**, *64*, 112-122. (**b**) Sheldrick, G. M. *SHELXL-2013*. University of Göttingen, Göttingen, Germany, 2013. (**c**) Sheldrick, G. M. *SHELXL-2014*. University of Göttingen, Göttingen, Germany, 2014. (**d**) Sheldrick, G. M. *SHELXL-2014/6*. University of Göttingen, Göttingen, Germany, 2014. (**e**) Sheldrick, G. M. *SHELXL-2014/7*. University of Göttingen, Göttingen, Germany, 2015. (**f**) Sheldrick, G. M. *SHELXL*. University of Göttingen, Göttingen, Germany, 2015. (**g**) Sheldrick, G. M. *SHELXL-2016/6*. University of Göttingen, Göttingen, Germany, 2015. (**h**) Sheldrick, G. M. *SHELXL-2018/1*. University of Göttingen, Göttingen, Germany, 2015.

(**10**) (**a**) *SHELXTL*, Version 2008/4, Bruker-AXS, Madison, Wisconsin, 2008. (**b**) *SHELXTL*, Bruker-AXS, Madison, Wisconsin, 2013. (**c**) *SHELXTL*, Bruker-AXS, Madison, Wisconsin, 2014. (**d**) *SHELXTL*, Bruker-AXS, Madison, Wisconsin, 2015. (**e**) *SHELXTL*, Bruker-AXS, Madison, Wisconsin, 2016. (**f**) *SHELXTL*, Bruker-AXS, Madison, Wisconsin, 2020.

(**11**) Spek, A.L. *PLATON SQUEEZE*: A Tool for the Calculation of the Disordered Solvent Contribution to the Calculated Structure Factors. *Acta Crystallogr., Sect. C: Struct. Chem.* **2015**, *71*, 9-18.

(**12**) See http://checkcif.iucr.org/

(**13**) Neese, F. Software Update: the ORCA Program System, Version 4.0 *WIREs Comput. Molec. Sci.*, 2018, 8, e1327.

(**14**) (**a**) Perdew, J. P.; Burke, K.; Ernzerhof, M. Generalized Gradient Approximation Made Simple. *Phys. Rev. Lett.* **1996**, *77*, 3865-3868. (**b**) Adamo, C.; Barone, V. Toward Reliable Density Functional Methods Without Adjustable Parameters: The PBE0 Model. *J. Chem. Phys.* **1999**, *110*(13), 6158-6170.

(**15**) (**a**) Weigend, F.; Ahlrichs, R. Balanced Basis Sets of Split Valence, Triple Zeta Valence and Quadruple Zeta Valence Quality for H to Rn: Design and Assessment of Accuracy. *Phys. Chem. Chem. Phys.* **2005**, *7*, 3297-3305. (**b**) Pantazis, D. A.; Chen, X.-Y.; Landis, C. R.; Neese, F. All-Electron Scalar Relativistic Basis Sets for Third-Row Transition Metal Atoms. *J. Chem. Theory Comput.* **2008**, *4*, 908-919.

(**16**) Neese, F.; Wennmohs, F.; Hansen, A. Efficient and Accurate Local Approximations to Coupled-Electron Pair Approaches: An Attempt to Revive the Pair Natural Orbital Method. *J. Chem. Phys.* **2009**, *130*, 114108. (b) Izsák, R.; Neese, F. An Overlap Fitted Chain of Spheres Exchange Method. *J. Chem. Phys.* **2011**, *135*, 144105.

(**17**) (**a**) van Lenthe, E.; Snijders, J. G.; Baerends, E. J. The Zero-Order Regular Approximation for Relativistic Effects: The Effect of Spin-Orbit Coupling in Closed Shell Molecules. *J. Chem. Phys.* **1996**, *105*, 6505-6516. (**b**) van Lenthe, J. H.; Faas, S.; Snijders, J. G. Gradients in the ab initio Scalar Zeroth-Order Regular Approximation (ZORA) Approach. *Chem. Phys. Lett.* **2000**, *328*, 107-112. (**c**) van Lenthe, E.; van der Avoird, A.; Wormer, P. E. S. Density Functional Calculations of Molecular Hyperfine Interactions in the Zero Order Regular Approximation for Relativistic Effects. *J. Chem. Phys.* **1998**, *108*, 4783-4796.

(**18**) van Wüllen, C. Molecular Density Functional Calculations in the Regular Relativistic Approximation: Method, Application to Coinage Metal Diatomics, Hydrides, Fluorides and Chlorides, and Comparison with First-Order Relativistic Calculations. *J. Chem. Phys.* **1998**, *109*, 392-399.

(**19**) Klamt, A.; Schüürmann, G. COSMO: A New Approach to Dielectric Screening in Solvents with Explicit Expressions for the Screening Energy and Its Gradient. *J. Chem. Soc., Perkin Trans. 2* **1993**, 799-805.

(**20**) (**a**) Pulay, P. Convergence Acceleration of Iterative Sequences. The Case of SCF Iteration. *Chem. Phys. Lett.* **1980**, *73*(2), 393-398. (**b**) Pulay, P. Improved SCF Convergence Acceleration. *J. Comput. Chem.* **1982**, *3*(4), 556-560.

(**21**) *Molekel*, Advanced Interactive 3D-Graphics for Molecular Sciences, Swiss National Supercomputing Centre. https://ugovaretto.github.io/molekel/
